# Supplementary material for: Peer-assisted HIV partner notification services to strengthen index partner testing for newly diagnosed men who have sex with men in coastal Kenya
Source: PLoS One. 2025 Oct 7;20(10):e0333707. doi: 10.1371/journal.pone.0333707 (PMC12503256; doi:10.1371/journal.pone.0333707)
Supplement: S3 Appendix — (ZIP) [file pone.0333707.s003.zip › Deidentified IDI Transcript_1012.docx]

**Participant characteristics:**

Age: 30-34

Sexuality: Bisexual

Education level: Primary

Days between enrollment and IDI: 97 days

Mobilization strategy: OST

Final PNS Strategy: HCP/PM

**Partners identified: 1**

**[INTERVIEWER]**: The recorder is now on. We are carrying out this interview on [DATE] at [CLINIC_A], your study number is 1012. We will not be using your names anywhere during the recording for confidentiality purposes.

**[PARTICIPANT]:** Ok

**[INTERVIEWER]:** I would like to refresh you on what the study entails so that we can be at per with today's discussion.

**[PARTICIPANT]**: Ok

**[INTERVIEWER]**: So, what we are saying is that PNS study involves notifying partners of index patient on their potential exposure to HIV infection, therefore in this study it is important for us to discuss about sexual partners with the one who is HIV infected. The index patient is linked to care and for those who are HIV negative there is a prevention measure which we offer and that is "PREP". In regard to notifying partners we have different methods we can use to conduct PNS. One of the ways is giving contact information of the partners to the health provider so that he/she can make an anonymous telephone call, we can also make a contract between the health provider and the index patient where we agree to give you time and bring your partner/s for testing. When the agreed time elapses then we will have to come in as health provider and help you notify the partner. We can also give you the OST so that you can give your partner/s to test for HIV. Are we together?

**[PARTICIPANT]:** Yes

**[INTERVIEWER]**: While doing the above we make sure to maintain confidentiality always.

**[PARTICIPANT]**: OK

**[INTERVIEWER]**: Therefore, as it is PNS has been going on for a while now for the general population, but we don't know how PNS can be conducted among MSM, Transgender women, and that is why we are carrying out this study. We would like to learn from the horses mouth themselves on how we can better PNS services on this group.

**[PARTICIPANT]**: OK

**[INTERVIEWER]**: In this discussion today, we would like to understand your experiences with PNS since you enrolled to the study, whether you are aware that your partner was notified and if there is any change in your relationships so far.

**[PARTICIPANT]**: OK. Thank you

**[INTERVIEWER]**: You are welcome. How have you been ever since, it's been a while since we last saw each other

**[PARTICIPANT]:** I have been doing fine

**[INTERVIEWER]:** Sometime back we were informed that you were not feeling well

**[PARTICIPANT]**: No, I have been fine

**[INTERVIEWER]:** Ok, how have you been so far ever since you knew your status.

**[PARTICIPANT]**: I have been ok

**[INTERVIEWER]:** How has ART been so far,

**[PARTICIPANT]**: The ARV's have been ok, no challenges whatsoever

**[INTERVIEWER]**: OK, that's nice to hear. Let me take you back to the last time you came hear for testing, what made you want to know your HIV status at that moment

**[PARTICIPANT]:** My health was deteriorating, and I started suspecting that I might be infected that's why I came here for testing. There is also a friend of mine who came to visit me at [NEIGHBORHOOD_A] where I stay. He talked to me about HIV and probably saw the condition I was in and advised me to test and know my exact status. I did an OST back then, but I was not so sure with the results at the time and therefore came to the clinic for confirmation.

**[INTERVIEWER]**: Ok, how frequent did you test for HIV back then

**[PARTICIPANT]:** I was not testing as frequent as I should

**[INTERVIEWER]**: So how long was it since you tested at that time you did an OST test

**[PARTICIPANT]**: It was almost a year before I tested again. I remember I had tested at my work place and the results were ok, I was HIV negative at the time when health providers came and offered HIV testing services at my work place.

**[INTERVIEWER]**: What risks do you think exposed you to HIV infection?

**[PARTICIPANT]**: I never used condoms before at all

**[INTERVIEWER]:** How is that so?

**[PARTICIPANT]**: I trusted my partners and did not have any reasons at all to use protection

**[INTERVIEWER]**: OK, you just said that there is someone who talked to you about HIV testing, did you understand everything he talked to you about?

**[PARTICIPANT]**: Yes, I understood him pretty much and even asked me of my sexual partners. As you know some one like me with a family is not easy to come out in the open but with the kind of conversation, we had I developed trust in him and opened about my sexual partners.

**[INTERVIEWER]:** I believe you used an OST kit for HIV testing at first, did he explain to you what to expect when you come to the clinic?

**[PARTICIPANT]**: Yes, when I came here, we did the rapids test, but results were discrepant and therefore had to do another third test and waited for almost two hours for my results.

**[INTERVIEWER]**: Do you understand why we had to do the third test

**[PARTICIPANT]:** Not really, but the rapids result was discrepant and therefore the health provider mentioned that there is a third test that will provide my exact status.

**[INTERVIEWER]:** Ok, the third test is called an RNA test. This test identifies HIV virus earlier compared to the rapid test. It can identify HIV virus as early as two weeks of infection.

**[PARTICIPANT]**: OK

**[INTERVIEWER]**: Is there any possibility that you were given any learning materials about HIV or anything that gave you an idea of what to expect when you come to the clinic?

**[PARTICIPANT]**: What I remember is that he advised me on testing and gave me the OST after getting the results from OST he told me that its important for me to come to the clinic for confirmation, that's all. I don't remember getting any learning materials from him except for information and advice he gave me.

**[INTERVIEWER]**: Ok, so what can you say of the advice and information that he gave you?

**[PARTICIPANT]**: What I can say is that the information was so helpful, moreover am so grateful because I was in a bad condition back then and I needed that kind of advice

**[INTERVIEWER]**: Ok, so what do you think can be done so that more MSM can come out and get tested.

**[PARTICIPANT]**: Ok, when he asked me of my sexual partners, I told him that I had Two male sexual partners. One of them I was seeing much often but the other it has been a while since I last saw him. For someone with a family its not easy to know if they have other relations especially with a fellow man but with the advice he gave me what I did is that I showed him my sexual partner and even showed him where he lived, that way he will have to use his own means to convince him to test without getting involved myself

**[INTERVIEWER]**: Was it the first time for you to use an OST kit?

**[PARTICIPANT]**: Yes, it was

**[INTERVIEWER]**: I remember you started ART immediately after you knew your HIV status, do you believe that helped you?

**[PARTICIPANT]:** ART helped me a lot, I am glad I started it as soon as possible

**[INTERVIEWER]:** Ok, what about the counselling you received. What can you say about it?

**[PARTICIPANT]**: The counselling helped me because before then I did not know what to expect and how to go about with life, but after the counselling I learnt a lot and I was able to make sound decision regarding my life and now I am fine.

**[INTERVIEWER]**: OK, now I would like us to discuss about the sexual partner that you mentioned the first-time during enrollment.

**[PARTICIPANT]:** Mmmmh

**[INTERVIEWER]**: You mentioned to me only one sexual partner who is your wife.

**[PARTICIPANT]:** Yes

**[INTERVIEWER]**: During that time was PNS explained to you in a way that you understood what it entails?

**[PARTICIPANT]**: According to what the health provider explained to me I can say I did understand about PNS. It was all about my partner also knowing her HIV status so that she can also start ART if necessary, but then it turned out that my wife was already on medication, she knew her status before I did.

**[INTERVIEWER]**: Ok, so your wife is already on medication as well. As of now can you tell whether all your partners have been notified and possibly tests for HIV?

**[PARTICIPANT]**: Yes, we come from the same area with my other male partner, he has tested for HIV and right now on ART as well.

**[INTERVIEWER]:** How is your wife fairing?

**[PARTICIPANT]**: She is doing fine. She knows her status and living positively.

**[INTERVIEWER]**: That is good. What do you think of the methods that were used to notify your partners, is there any change in any of your relationships so far?

**[PARTICIPANT]:** As of now I am still in my relationships and I have no reason to separate from my wife

**[INTERVIEWER]:** what of the other relationship with the male partner

**[PARTICIPANT]**: I am still in contact with him and our relationship is just fine but discreet

**[INTERVIEWER]**: Ok, so as it is PNS has not in any way change your relationship with either of your partners

**[PARTICIPANT]**: No, it hasn't, my relationships are fine with both my partners

**[INTERVIEWER]**: What you have said is that both your partners know their status and that you were able to disclose your status as well

**[PARTICIPANT]:** Yes, and they are all on ART

**[INTERVIEWER]**: Ok that is a good thing. You have told me that you disclosed to your partners right, what about at your workplace, could there be someone who possibly knows of your condition?

**[PARTICIPANT]**: No, I have not talked to anyone about my status at work

**[INTERVIEWER]:** Ok, therefore PNS now is part and parcel of the services given to anyone who tests HIV positive. Notification is done for all the sexual partners of the index within one year, this means even the sexual partner who they had sex only once.

**[PARTICIPANT]**: Mmmm

**[INTERVIEWER]:** As previously discussed during enrollment, what was going through your mind as we talked about your partners

**[PARTICIPANT]:** At first, I thought it was going to be difficult to notify my partners, I didn't know how to go about it. Afterwards when we had a discussion with one of the health providers, I got to know different methods and it turned out to be a success. I described my partner to the health provider and showed him where to find him and the rest was easy.

**[INTERVIEWER]:** Ooooh OK, all this you discussed it probably with my colleague because what I remember you mentioning to me was only the wife right?

**[PARTICIPANT]**: Yes,

**[INTERVIEWER]**: What made you not talk about your male partner with me instead, I would like to understand you better

**[PARTICIPANT]**: You know people are always discreet with this kind of things but afterwards I thought its good to be open so that I can get help

**[INTERVIEWER]:** OK, that's quite normal. It's a good thing that you finally opened up at least to someone who could be of help to you

**[PARTICIPANT]:** yes

**[INTERVIEWER]:** Okay, therefore how many people can we say you have had sexual encounters with in the last one year apart from the ones you have mentioned?

**[PARTICIPANT]:** Its just the two partners my wife and the male partner ie. 2 partners

**[INTERVIEWER]:** Ok, could you please share some of the ways that can make one comfortable to open up and discuss about their sexual partners

**[PARTICIPANT]**: Its not easy but I can take a good example of your colleague who came at [NEIGHBORHOOD_A], I was with my friends, but he advised us and gave us a lot of information. Through that those who are interested can come forth. What I can say is that the counselling was very important, and he was also very patient with me not to say that I didn't like your services but rather I needed more time for me to open up.

**[INTERVIEWER]:** Its ok, that's good. Do you think PNS can be effective for gay, MSM and even transgender women

**[PARTICIPANT]:** I don't think I understand your question

**[INTERVIEWER]**: Ok, as you are aware PNS is notifying partners of index so that they can also get tested and know their status. What better ways do you think PNS can be more effective to MSM, gay and even Transgender women.

**[PARTICIPANT]**: It just like the way I know my status, through counselling that can be workable because you just can't follow anyone out of the blue. As I said this kind of things are usually discreet.

**[INTERVIEWER]:** What you are saying is that there should be trust for someone to open up.

**[PARTICIPANT]:** Yes

**[INTERVIEWER]:** Ok, as mentioned earlier PNS is a service that is given to anyone who tests HIV positive. This has been going on for some time now especially for the general population. We would like the same to be done for gay, MSM or even transgender, do you think this will work?

**[PARTICIPANT]**: I believe it will work. Most people think that a man having sex with fellow man is so strange, yet it is normal, and this thing happen. Its high time people accept that such relationships do exist and PNS would be just as effective with MSM's as well.

**[INTERVIEWER]**: Therefore, do you think there will be any challenges notifying partners of gay, MSM or transgender

**[PARTICIPANT]**: you know when people take being an MSM or gay as immoral or a bad thing they shy off from it then this way its going to be difficult.

**[INTERVIEWER]:** So, its upon us health providers to talk about being gay, an MSM as something that is normal so that our clients can not shy off.

**[PARTICIPANT]**: Yes

**[INTERVIEWER]**: Therefore, what is the importance for partner notification?

**[PARTICIPANT]:** its very important as those partners will be tested and for those that are HIV positive can start ART early enough compared to when they are out there not knowing what is happening in their life

**[INTERVIEWER]**: What of those that are HIV negative?

**[PARTICIPANT]:** The ones who are HIV negative can also learn a thing or two because you never know what your tomorrow holds

**[INTERVIEWER]:** That's true, there are so many ways of preventions and one of them is PREP for those who are HIV negative. PREP are drugs that is taken daily and when it reaches a time someone wishes they no longer need PREP then they can stop taking it at any given point.

**[PARTICIPANT]**: Does the drug have any side effect

**[INTERVIEWER]:** The side effects are quite minimal

**[PARTICIPANT]**: Ok

**[INTERVIEWER]:** Like we said earlier we have different methods of carrying out PNS. First, we can use the health provider to make anonymous phone call to partner, second the peer mobiliser can give partner an OST kit, Third the peer mobiliser can give you an OST kit to take to your partner for testing, another way is that the health provider can help the index patient to disclose to his partner e.g. the index can come to the clinic with their partner for couple testing. The peer mobiliser can also help the index patient to invite their partner for testing. The index can also give OST kit to their partner to test for HIV.

**[PARTICIPANT]:** ok

**[INTERVIEWER]:** What do you think of the above methods of partner notification

**[PARTICIPANT]**: The methods are just fine

**[INTERVIEWER]**: In your own opinion which method do you think works better than the other

**[PARTICIPANT]**: The one where I can give my partner an OST works better

**[INTERVIEWER]**: Is there any other method that you believe is effective for Partner notification other than the ones above?

**[PARTICIPANT]**: Not really, the one for OST is much favorable on my side and the peer mobilisers can come to places where we hang out mostly and probably issue OST kits to everyone keeping in mind that there may be someone they are targeting, doing so the targeted person will not suspect a thing.

**[INTERVIEWER]**: What you are saying is that we can use different methods for each partner

**[PARTICIPANT]:** yes of course.

**[INTERVIEWER]:** What period should we wait before we discuss about sexual partners with the index

**[PARTICIPANT]**: In my opinion immediately one tests positive for HIV then they should be able to discuss about their sexual partners because when one is given time, they can even be reluctant about it. You could even give the index an option, something like, 'now that you know you are HIV infected, we would like to discuss about your partners, are you comfortable we talk about this now or we can schedule it for another time?' As for me its better to discuss about my partners immediately

**[INTERVIEWER]**: Okay, there is one of the methods of PNS where the health provider makes anonymous phone call to the partner and invite them for testing, if it were you making the phone call what would you say to the partner that can convince them to come for testing?

**[PARTICIPANT]**: The partner may be having questions like, 'where did you get my phone number from?' but it depends with an index who is sure that his lover could be having multiple partners therefore it will not be easy for the partner to actually tell how and who probably is involved with the entire situation.

**[INTERVIEWER]:** When we make the phone call, we can say something like, "my name is ....and I am calling you from [RESEARCH_INSTITUTION], is this [PERSON_A] (not actual name). I am calling you in regards of a health matter and its important for you to come to the [HOSPITAL_C] for more information. Please give me a call once you get to the hospital'. Or maybe something like, 'My name is ... I am making this phone call from the [HOSPITAL_C], is this [PERSON_A]. We are currently conducting a study called PNS and you have been mentioned among the partners that are needed to come to the clinic for HIV testing, kindly find time and let us know'. Do you think those words are good enough to use while inviting the partner to the clinic or you have better words that we could possibly use instead?

**[PARTICIPANT]:** You know according to the different methods that we discussed earlier it won't be easy for the partner to know who gave out their number but out of curiosity one would always ask themselves about who could be that person who gave out my number.

**[INTERVIEWER]**: That's true, this kind of question can come up and it's something that probably anyone making that phone call expects and at other times the question may not even pop out.

**[PARTICIPANT]:** oooh OK, then there is no problem at all to make the phone call because people come to hospital for various reasons and that can make one come to clinic to find out more about the health concern that the health care provider is referring to.

**[INTERVIEWER]**: Do you have any opinion on how to make the index open up and be comfortable to discuss about their partner

**[PARTICIPANT]**: What I can say is that for the advice and counselling is important just like myself I didn't know whether my partners know about their status, it is my responsibility to make sure that my partner/s are also tested, if its difficult for me then probably the health provider can come in with different ways that we can use to get my partners tested as well.

**[INTERVIEWER]:** This way can be the same as we discussed earlier ie different methods of partner notification, right?

**[PARTICIPANT]:** Yes

**[INTERVIEWER]:** We are almost done with our discussion what I would like to know is whether there is a better way to make PNS effective and a success to gay, MSM or even Transgender women.

**[PARTICIPANT]:** What I can say is that the services that are being offered at the clinic are very good

**[INTERVIEWER]:** Ok, this is point we are now done with today's discussion, is there anything that you would like to add or say about PNS

**[PARTICIPANT]**: According to the advice and our discussion up to this point I am Ok.

**[INTERVIEWER]:** Ok, welcome and thank you for your opinions and everything that we discussed today. Thank you also for being brave enough and openly discuss about some of the things that we could not talk about before. We have now concluded our interview and thanks again for your time. Much appreciation.
