# Supplementary material for: Peer-assisted HIV partner notification services to strengthen index partner testing for newly diagnosed men who have sex with men in coastal Kenya
Source: PLoS One. 2025 Oct 7;20(10):e0333707. doi: 10.1371/journal.pone.0333707 (PMC12503256; doi:10.1371/journal.pone.0333707)
Supplement: S3 Appendix — (ZIP) [file pone.0333707.s003.zip › Deidentified IDI Transcript_1018.docx]

**Participant characteristics:**

Age: 30-34

Sexuality: Bisexual

Education level: Primary

Days between enrollment and IDI: 46 days

Mobilization strategy: OST

Final PNS Strategy: HCP/PM

**Partners identified: 8**

**[INTERVIEWER]**: Okay, the recorder is now on. I kindly ask you to be audible for easy recording. I hope you remember me, I was the one who attended to you before .

**[PARTICIPANT]**: Yes, I do, but I did lose your phone number.

**[INTERVIEWER]**: You did? No problem I will give it you afterwards. Today is [DATE]. We are doing this interview in [CITY_A] and the number you were assigned was 1018. How are you today?

**[PARTICIPANT]**: I'm good! Very good!

**[INTERVIEWER]**: You seem excited indeed! Just to remind you about the study, Partner Notification involves notifying the sexual partners of someone who has tested HIV Positive of the importance of getting tested without revealing the identity of the participant. There's a procedure we follow that ensures the partners will not know how we got their contact information or who gave it to us, we value our participants' confidentiality.

The participant who has been tested and turned out positive is called an index patient, just like yourself we can say you are an index patient. Participation in the study is voluntary, do not feel any pressure to participate from anyone.

**[PARTICIPANT]**: Ok.

**[INTERVIEWER]**: When carrying out PNS, there might be a few negative outcomes and that's why we choose not to reveal one's identity to the partners to avoid putting them at risk.

PNS has been happening for a while to all clients who go to the VCT and test HIV positive, but its Only lately that a study has been developed to know how it can be done among MSM. I hope that recap reminds you what the study is about so you can understand what exactly we want to discuss today.

**[PARTICIPANT]:** Ok

**[INTERVIEWER]:** How have you been since learning about your status?

**[PARTICIPANT]:** I have been doing fine.

**[INTERVIEWER]:** Okay. Have you had any challenges with ART?

**[PARTICIPANT]**: No, just feelings of pain around my waist.

**[INTERVIEWER]:** Your waist has been aching? Okay, and have you had any problems or challenges since you started using the drugs?

**[PARTICIPANT]:** No. Things are normal

**[INTERVIEWER]:** Okay. Last time you got tested, what made you come for an HIV test?

**[PARTICIPANT]:** I get tested yearly so it was only a routine and decide test again this year.

**[INTERVIEWER]:** So, you get tested regularly?

**[PARTICIPANT]:** Yes, I do.

**[INTERVIEWER]:** How many times do you get tested in a year?

**[PARTICIPANT]:** Twice

**[INTERVIEWER]:** Okay. And among risks of HIV infection, what do you think could have possibly exposed you to infection.

**[PARTICIPANT]:** Maybe a condom burst.

**[INTERVIEWER]:** A condom burst? So, what you trying to say is that you use a condom every time you have sex....

**[PARTICIPANT]:** Mmmmh...But at times condom did burst,

**[INTERVIEWER]:** so, what did you do when the condom burst?

**[PARTICIPANT]:** I did not know.

**[INTERVIEWER]:** You did not know when the condom bursts?

**[PARTICIPANT]**: I did not know, I was drunk, so I was not fully aware.

**[INTERVIEWER]:** Ooh! So, you did not realize when the condom burst because you were drunk, now I understand when a condom bursts, one can get help by visiting the clinic and get drugs to prevent them from acquiring the virus.

**[PARTICIPANT]:** Right there and then?

**[INTERVIEWER]:** Yes, that is if one comes within 72 hours since the incident happened. One will then be tested for HIV, when they are HIV negative, they are given drugs to prevent them from getting infected.

**[PARTICIPANT]:** I did not know about all this. If I did, I would have come very quickly!

**[INTERVIEWER]:** So that's what was supposed to happen.

**[PARTICIPANT]:** when one gets infected is there a way of reversing it?

**[INTERVIEWER]:** No, it becomes like spilt water, you cannot collect it back. So tell me have you had any other sexual partner ever since?

**[PARTICIPANT]:** Male or female?

**[INTERVIEWER]:** Either

**[PARTICIPANT]:** I do not have...(pause) a partner.

**[INTERVIEWER]:** So, you have not been on any new sexual relations?

**[PARTICIPANT]:** I do not have a partner (laughs)...you are scaring me.

**[INTERVIEWER]:** What I am trying to ask is, ever since you learnt your HIV status, have you had any new sexual relationship with anyone?

**[PARTICIPANT]:** Yes

**[INTERVIEWER]:** I understand that you have had a new partner after learning your HIV status. Have you guys tested for HIV together or even disclose your status to your partner/s?

**[PARTICIPANT]:** Mmh mmh.... Not yet, we have not tested together

**[INTERVIEWER]:** And do you perhaps know their HIV status?

**[PARTICIPANT]:** No, I do not

**[INTERVIEWER]:** Wouldn't you like to know their status? Have you not discussed about testing?

**[PARTICIPANT]:** No. not yet.

**[INTERVIEWER]:** Ok, so what are your plans on the same?

**[PARTICIPANT]:** We do not live together. We have had sex only once and I used a condom.

**[INTERVIEWER]:** Ok, have you had any other sexual relation apart from this one?

**[PARTICIPANT]:** There has been another one, an Arab. We were together only once as well.

**[INTERVIEWER]:** And you used a condom every time?

**[PARTICIPANT]:** Yes, I did... (coughs)

**[INTERVIEWER]:** What made you come here for testing the very first time?

**[PARTICIPANT]:** I was approached by a peer educator.

**[INTERVIEWER]:** What did the two of you discuss. How did he manage to convince you to come at the clinic?

**[PARTICIPANT]:** He came to the beach and talked to me, so I agreed with him and followed him here where I got tested with that device...

**[INTERVIEWER]:** The self-test device?

**[PARTICIPANT]:** Yes, then you later took out my blood.

**[INTERVIEWER]:** What exactly did you two talk about that made you agree to come get tested?

**[PARTICIPANT]:** He said that I will benefit from counselling, be given a self-test device for HIV and that I would receive some allowance afterwards.

**[INTERVIEWER]:** So, he told you about the self-test kit and reimbursement.

**[PARTICIPANT]:** Yes

**[INTERVIEWER]:** Is there a possibility that he told you of different types of kits that we will be using to carry out HIV test.

**[PARTICIPANT]:** Yeah! He told me that there was that of the fingerpick and another one where you would draw some blood from the vein on my arm.

**[INTERVIEWER]:** What did he tell you about drawing blood? Did he tell you why we do that?

**[PARTICIPANT]:** He only told me that once I am tested and turn to be HIV positive, several tests would be done on my blood.

**[INTERVIEWER]:** What other tests did he talk to you about apart from the self-test kit.

**[PARTICIPANT]:** No other.

**[INTERVIEWER]:** ok, what I would like to find out is whether he mentioned of RNA test.

**[PARTICIPANT]:** No. he did not.

**[INTERVIEWER]:** There is another test that we would have used if you tested negative. This test can show if one had been infected as early as 2 weeks.

**[PARTICIPANT]:** No, not yet!

**[INTERVIEWER]:** On your part this test did not happen because you already tested positive from the oral self-test kit.

**[PARTICIPANT]:** Mmmmh...

**[INTERVIEWER]:** But like you said, blood was taken for further test to find out things like the amount of virus in the blood...

**[PARTICIPANT]:** But I have not gotten the results!

**[INTERVIEWER]:** Yes, that because the results are not out yet, but when they do we will explain them to you.

**[PARTICIPANT]**: Okay.

**[INTERVIEWER]:** So, did you understand what the peer educator talked to you about?

**[PARTICIPANT]:** Yes, I understood.

**[INTERVIEWER]:** So, you know how the oral self-test kit works and how long did it take to show the results?

**[PARTICIPANT]:** Yes.

**[INTERVIEWER]:** How much time did you wait to interpret the results?

**[PARTICIPANT]:** Like 5 to 7 minutes.

**[INTERVIEWER]:** Was it your first-time t0 use the oral self-test kit?

**[PARTICIPANT]:** Yes, it was.

**[INTERVIEWER]**: Was there any material he gave you that contained information about the study or HIV testing? A card or any other material with explanation?

**[PARTICIPANT]**: After testing?

**[INTERVIEWER]**: No, before testing.

**[PARTICIPANT]:** A card? No, I was not given a card or any other material with explanation about HIV Testing. I once went to [ORGANIZATION_B] and was given a card thou'. The mobiliser only talked to me about the OST

**[INTERVIEWER]:** What was your experience meeting with the mobilizer and being brought here?

**[PARTICIPANT]**: I had already planned to get tested around the next day so when he came to tell me about it I chose to do it there and then.

**[INTERVIEWER]:** Ok, you already had plans to get tested, I see. That was quite a coincidence him coming to invite you for testing?

**[PARTICIPANT]**: Yeah, it was.

**[INTERVIEWER]**: Ok, as it is men who have sex with men are among those who are most at risk to get HIV. What do you think can be done to encourage them to get tested more?

**[PARTICIPANT]:** I think you should see them, know them then get them to test...

**[INTERVIEWER]:** What can be done to encourage them to get tested?

**[PARTICIPANT]:** (Mumbles)

**[INTERVIEWER]:** Okay, let me put it this way, what can we do make more homosexual men to come out in large numbers to get tested.

**[PARTICIPANT]**: Maybe you could have meetings.

**[INTERVIEWER]:** Meetings...What kind of meetings?

**[PARTICIPANT]**: (Laughs) you know its hard to know an MSM, so maybe if you knew them you could tell them about the meeting discussing HIV. So maybe if you did that they would come out in large numbers.

**[INTERVIEWER]**: Sorry, Do what?

**[PARTICIPANT]:** Knew them. because.... (pauses)

**[INTERVIEWER]:** Yes, go on don't hold back, feel free to speak up

**[PARTICIPANT]**: Because If you knew them and where they lived you could follow them and tell them the importance of getting tested.

**[INTERVIEWER]:** And what if we do not know where they live?

**[PARTICIPANT]**: That will now be hard.

**[INTERVIEWER]:** And what do you propose to be discussed in those meetings?

**[PARTICIPANT]**: In that meeting? ... (pauses)

**[INTERVIEWER]**: You mean a meeting where they would be educated on the importance of testing or what do you mean?

**[PARTICIPANT]**: Yeah! Such a meeting.

**[INTERVIEWER]:** Ok let's talk about yourself. How did you take the results when it turned out that you are infected?

**[PARTICIPANT]:** I panicked. I panicked about my life.

**[INTERVIEWER]**: What about your life were you thinking of?

**[PARTICIPANT]**: I remember thinking that it was the end of me! I was hopeless.

**[INTERVIEWER]:** You thought all was over, your plans were ruined. What about now? How are you since then? Do you still feel hopeless?

**[PARTICIPANT]**: Now I think things are going okay.

**[INTERVIEWER]**: How did you overcome such thoughts?

**[PARTICIPANT]**: I was thinking about committing suicide, but... (pauses) I was advised to keep taking the drugs and continue living.

**[INTERVIEWER]**: Who did you talk? A counsellor?

**[PARTICIPANT]**: No, myself but from the way they saw me I think they knew I wanted to kill myself and talked to me to take drugs and it would be okay and that why I followed through.

**[INTERVIEWER]**: Who was this that gave you the morale to take drugs and encouraged you? Did you discuss your status with anyone?

**[PARTICIPANT]**: Yeah...I opened up to the peer mobilisers ( meant the community team)

**[INTERVIEWER]:** They encouraged you, been by your side...

**[PARTICIPANT]**: Yes, they did. You also helped me too.

(Both laugh)

**[INTERVIEWER]**: Yes! Me too. we have been talking constantly. When one turns out positive, it is not the end of life. The most important thing is to take your drugs. And you were lucky to discover early while you are still strong and can take your drugs. When one takes the medication early, they avoid a lot of illnesses. When one is infected with HIV the body becomes less immune to diseases but taking your drugs prevents that!

**[PARTICIPANT]**: Mmmh!

**[INTERVIEWER]**: And nowadays you cannot tell the difference between someone who is taking ARV's and a non-infected person they are all the same. This is because such a person has accepted themselves and started their drugs early. If you wait till you are weak and brought to the hospital, those taking care of you will have to be notified of your status. But if you take your drugs early, you will remain healthy and only you will decide when to reveal your status and to whom. Right?

**[PARTICIPANT]**: Mmmh!

**[INTERVIEWER]**: Did you start taking medication immediately after knowing your status?

**[PARTICIPANT]**: No, it took me some time before I did.

**[INTERVIEWER]**: Oooooh, I remember you talked of the counsellor advising you on ART and trying to make you understand the importance of starting your medication early enough. The counselling you received until you finally accepted your condition.

**[PARTICIPANT]:** Yeah

**[INTERVIEWER]**: So, how has it been since you started ART?

**[PARTICIPANT]**: Mmmh!

**[INTERVIEWER]**: How do you feel about the drugs? How are they so far?

**[PARTICIPANT]:** They are okay.

**[INTERVIEWER]**: Have you experienced any challenges ever since you started ART

**[PARTICIPANT]**: No.

**[INTERVIEWER]:** Ok, please tell me, what are your opinions on the counselling you received ever since.

**[PARTICIPANT]**: Counselling about... (pauses) about taking the drugs? Or?

**[INTERVIEWER]:** All the counselling you have been getting since you got tested?

**[PARTICIPANT]:** The counselling has been about me taking my drugs and to stop thinking too much.

**[INTERVIEWER]**: Was it helpful?

**[PARTICIPANT]**: Yes, it was helpful.

**[INTERVIEWER]**: Has it been of any importance to you since getting tested?

**[PARTICIPANT]**: Yes, it has been very helpful.

**[INTERVIEWER]**: Ok fine. Now I would like us to discuss about the sexual partners you mentioned to me the last time you were in the clinic.

**[PARTICIPANT]**: Ok

**[INTERVIEWER]**: How was our study introduced to you, did you understand what PNS entails

**[PARTICIPANT]**: Which one

**[INTERVIEWER]:** I mean about notifying partners of their possible risk of HIV infection.

**[PARTICIPANT]:** Yes.....

**[INTERVIEWER]**: Kindly please elaborate briefly what you understood about PNS

**[PARTICIPANT]**: I understood that my sexual partners should also come and get tested so that they can know their current HIV status and benefit with ART whenever necessary.

**[INTERVIEWER]:** OK, please allow me to take you back a little bit, we also discussed of different ways of notifying your partners that you could choose from. We talked of anonymously making a telephone call to your partners and other methods as well, how do you feel about them? Do you remember some of the methods we discussed we can use to notify your partners?

**[PARTICIPANT]**: Mmmm.... maybe something like giving the health provider contact information of the partner, I could also come with the partner/ s myself for testing.

**[INTERVIEWER]**: Ok, what you choose among the different methods is that the health provider should anonymously make a telephone call to your partners to notify them of the possible risk of infection, do you believe that was on of the ways that could work best for you and why

**[PARTICIPANT]**: Yes, that was the way, but as it is now, I don't have a phone and lost all their contacts

**[INTERVIEWER]:** How else do you think we can be able to reach them

**[PARTICIPANT]**: Now the other way I think is through the meetings I mentioned earlier. Those meetings can attract them as they would want to know the importance of the discussions going on. They maybe passing by while the meeting is going on and it could be a an amazing coincidence.so through that you could get some of them. As of now you never know, there is a possibility that they have shifted from where they once lived. They could have shifted to [CITY_G] or [CITY_F] , Therefore having the phone numbers could have been much easier.

**[INTERVIEWER]**: What if we were having the discussion about your partners all over again, would you choose the same method you did before for notification.

**[PARTICIPANT]**: Yes, that is the best, what I would do is if I get to see them again, I would take their phone contacts and share them with you

**[INTERVIEWER]:** Ok fine. Is there a possibility that you would know any of your partners was notified and asked to come for HIV testing?

**[PARTICIPANT]**: There is one who stays in [NEIGHBORHOOD_B] (name mentioned in audio) who came here for testing.

**[INTERVIEWER]:** So, you know of him being notified for HIV testing

**[PARTICIPANT]**: Yes

**[INTERVIEWER]:** Is there any other who you possibly know that he was notified and came for HIV testing?

**[PARTICIPANT]:** No, I only know of the one in [NEIGHBORHOOD_B]. There is this other one .... (name mentioned in audio)

**[INTERVIEWER]:** Oooh Yes. We are yet to get that one since you didn't give us any contact information. Could you possibly have the phone number today or know of a way we can reach him?

**[PARTICIPANT]**: (silence...).

**[INTERVIEWER]**: We can also give you time to go talk to him and bring him here for testing, or even give you OST kits to go give him instead.

**[PARTICIPANT]:** I may go tell him and he would start thinking I am the one who infected him, this one should come here himself

**[INTERVIEWER]:** How can we make that happen if we don't even know his phone number?

**[PARTICIPANT]**: He is the kind of person who has multiple sexual partners. He may think that I am the one who infected him, yet he could have been infected by other people. He can even send snippers to finish me off. He has a lot of money.

**[INTERVIEWER]**: What you trying to say is that you fear for your life and that's why you can't go and talk to him about it? How else do you think we can get to him?

**[PARTICIPANT]:** You can only get through to him if we get his phone number

**[INTERVIEWER]:** That seems could be quite a challenge for us right now, could you possibly describe where we can find him and possibly where he hungs out most?

**[PARTICIPANT]**: He normally likes hunging out at [LOCATION_A]. When you go there and ask for him you would find him and possibly get his phone number as well.

**[INTERVIEWER]**: Ok, so we have talked of the one from [NEIGHBORHOOD_B] and this other one who hungs out at [LOCATION_A]...

**[PARTICIPANT]**: Yes, those are the only ones that I know live in [CITY_A].

**[INTERVIEWER]**: Ok, now that you have an idea of some of the partner/s that have been notified, has there been any change with your relationship with them?

**[PARTICIPANT]:** I have not met any of them yet, so nothing has changed. Things are just the same.

**[INTERVIEWER]**: Could there be still a relationship with them. I.e between you and them

**[PARTICIPANT]**: Maybe just only when they call me if they want to have sex nothing else. They pay me for it.

**[INTERVIEWER]:** OK, have you possibly disclosed your status to anyone else?

**[PARTICIPANT]**: No, I haven't

**[INTERVIEWER]**: Do you have plans of telling anyone about your status

**[PARTICIPANT]**: No, I don't

**[INTERVIEWER]**: So, you are not ready to disclose to any one yet

**[PARTICIPANT]**: No, I can't dare disclose to anyone

**[INTERVIEWER]**: I remember the last time we were discussing about your partners you mentioned that you have a wife, don't you think it's important for her to know as well?

**[PARTICIPANT]**: OoooSoh that one, she lives in [NEIGHBORHOOD_C]

**[INTERVIEWER]:** Don't the two of you visit each other?

**[PARTICIPANT]:** No, we don't, we are separated

**[INTERVIEWER]:** Ok, so the two of you separated. For how long now?

**[PARTICIPANT]**: Its been three years now

**[INTERVIEWER]**: As it is you are not ready to disclose your status yet, what difference do you think it will make when people get to know your HIV status?

**[PARTICIPANT]:** It would be like exposing myself if I ever disclose my status to anyone.

**[INTERVIEWER]**: If I understand you clearly what you mean is that you don't trust anyone in your life at this point whom you would disclose your status to?

**[PARTICIPANT]**: Yes

**[INTERVIEWER]**: Has there been any challenges after PNS was done

**[PARTICIPANT]**: No, I have not experienced any difficulties after notification of my partners

**[INTERVIEWER]**: Ok that's good, I recall that when you first came here you were not able to trust me enough to admit that you are an MSM, how was it like discussing your sexual partners with me yet you didn't know me at all.

**[PARTICIPANT]**: You know, its not easy to tell a woman that you have sex with your fellow men

**[INTERVIEWER]:** I totally understand, you later opened though, what made you trust me enough and opened up to me about your sexual life?

**[PARTICIPANT]**: I know you are a health provider and in one way or another I would learn something from you and advise me better

**[INTERVIEWER]**: So that made you gain trust in me. Ok if I take you back a little bit, other than the sexual partners we talked about last time is there any other that you forgot to mention before?

**[PARTICIPANT]**: Yes, there is but I don't even know where they stay and it's also a long time since I have been with them

**[INTERVIEWER]:** In a period of one year is there any other sexual partner other than the ones we discussed before

**[PARTICIPANT]**: No, it's been quite a long time probably five to six years ago.

**[INTERVIEWER]**: Oooh ok. So, when we talk of PNS, this is something that is currently done for everyone in general who tests HIV positive in every facility. What do you think of it?

**[PARTICIPANT]**: I think it's a good thing

**[INTERVIEWER]**: Would you recommend PNS to others?

**[PARTICIPANT]:** Yes of course.

**[INTERVIEWER]:** Why do you think its important to carry out PNS to everyone

**[PARTICIPANT]**: They will fall sick if they are not notified early. Its important for partners of the one infected to be notified so that they can get care if necessary, at the right time.

**[INTERVIEWER]**: Do you believe PNS would be effective if it was done to MSM

**[PARTICIPANT]**: This would be amazing.

**[INTERVIEWER]**: Really, what challenges do you think would accrue due to PNS.

**[PARTICIPANT]:** No, it would have just been OK.

**[INTERVIEWER]:** In the beginning we discussed of different methods of PNS. I would like us to go through them again one after the other. First there is the one that a health provider will make an anonymous phone call to the partner, Secondly, we can use a peer mobiliser to distribute OST kits the same way you were recruited, Thirdly, the peer educator can go and distribute OST kits at hotspot. Fourth is that a health provider can help you invite you and your partner for testing, the fifth one is that a peer mobiliser can help you invite your partner for testing. There is also another one that you as an index we can give you an OST to give your partner. Which among the above discussed would have worked better for you?

**[PARTICIPANT]**: The one that I would prefer is getting an OST to give my partner/s

**[INTERVIEWER]:** Ok, so why do you prefer that one over the others?

**[PARTICIPANT]**: To me having the OST to give my partner is much better because whenever I meet them again then I would give them the kit to test themselves.

**[INTERVIEWER]:** How long did it take for PNS to be Introduced to you.

**[PARTICIPANT]**: It was introduced the same day

**[INTERVIEWER]**: Do you feel it was ok for it to happen as it did or you have a different thought on the same

**[PARTICIPANT]**: No, that was the best for me.

**[INTERVIEWER]**: ok, do you have a way that you would like us to talk to the partners when we make that phone call. Maybe certain words that you would have loved us to use instead.

**[PARTICIPANT]**: For me taking the OST is the best way that can make PNS effective

**[INTERVIEWER]**: Do you think that there is something that I could have done differently to make PNS for your partners much effective

**[PARTICIPANT]:** No, the way you carried out the discussion the first time is fine with me

**[INTERVIEWER]**: Is there any other thing or even thoughts that you want to add that you believe will make PNS successful

**[PARTICIPANT]:** No

**[INTERVIEWER]**: Is there any question that you would like to ask me or anything that you would like to know from me?

**[PARTICIPANT]**: No, I don't have any questions, Thank you.

**[INTERVIEWER]**: Ok, so we have come to the end of our discussion, thank you for your time and we are glad to have you here with us today. Asante sana
