# Supplementary material for: Peer-assisted HIV partner notification services to strengthen index partner testing for newly diagnosed men who have sex with men in coastal Kenya
Source: PLoS One. 2025 Oct 7;20(10):e0333707. doi: 10.1371/journal.pone.0333707 (PMC12503256; doi:10.1371/journal.pone.0333707)
Supplement: S3 Appendix — (ZIP) [file pone.0333707.s003.zip › Deidentified IDI Transcript_1049.docx]

**Participant characteristics:**

Age: 25-29

Sexuality: Gay

Education level: Vocation

Days between enrollment and IDI: 23 days

Mobilization strategy: OST

Final PNS Strategy: HCP/PM

**Partners identified: 3**

**[INTERVIEWER]:** Thank you very much for coming for the interview and agreeing to participate in this interview. As I had mentioned earlier, we will tape record this interview so that we will be able to capture every opinion, thoughts and ideas when we write a report. Are you still comfortable with tape recording?

**[PARTICIPANT]:** Yes am very much comfortable.

**[INTERVIEWER]:** How have you been doing since the last time we met?

**[PARTICIPANT]:** Am glad am doing okay, since I started treatment I feel am doing great, am thankful.

**[INTERVIEWER]:** How did you get through it?

**[PARTICIPANT]:** I joined [CBO_A] and there I received a lot of encouragement and support and that is why I gathered confidence and came for an HIV test. Some of the members even disclosed to me about their HIV status and they just look okay, so this also made it possible for me to get through it and become positive. Others even went ahead and showed me their pill bottles, initially I had perception that when one is HIV positive the next thing they will get into is a coffin. But after receiving support, information on HIV and even seeing people who have been living with HIV for many years and their health is just great now am viewing things differently.

**[INTERVIEWER]:** When did you learn that you are HIV positive?

**[PARTICIPANT]:** It's like a month ago.

**[INTERVIEWER]:** when did you join [CBO_A]?

**[PARTICIPANT]:** like 3 months ago

**[INTERVIEWER]:** Okay, you said that you received support, encouragement and information about HIV and that inspired you to go for an HIV test, apart from that is there any other reason that made you to decide and have an HIV test?

**[PARTICIPANT]:** Uuuh, I had some signs that were not there and I shared it with some members of [CBO_A] and I was told it better if I go for testing because it might be one of opportunistic infections, I also googled and saw same information on the net.

**[INTERVIEWER]:** can you please tell me more about the symptoms?

**[PARTICIPANT]:** yeah, they were like small rashes that appeared too close and were a bit painful at the beginning, I had them on the right side of my chest after I googled I saw that they looked similar like Herpes zoster and when I joined here in the research the doctor told me the same it was Herpes zoster and treated me.

**[INTERVIEWER]:** Let me take you back on your testing experience, you mentioned that you last tested a month ago, can you tell me more about your testing experience, were you a regular tester? How often were you used to test for HIV?

**[PARTICIPANT]:** The last time I tested before the one month ago was a year ago when I was going to [OVERSEAS_LOCATION] for work, am glad that I tested HIV negative. So I travelled to [OVERSEAS_LOCATION] but things didn't go so right there so I had to terminate my contract with the employer and came back within 3 months.

**[INTERVIEWER]:** When you say, things didn't go right what do you exactly mean?

**[PARTICIPANT]:** we had misunderstanding on salary and the work load, initially I knew I was going to work as a driver but when I reached there I was posted as a garden boy.

**[INTERVIEWER]:** Okay, so you joined [CBO_A] and received all the information on HIV, and how did you came to know about [RESEARCH_INSTITUTION]?

**[PARTICIPANT]:** I met a peer educator at [CBO_A] and he told me about [RESEARCH_INSTITUTION] and oral self-testing. I also showed him the rashes and he empowered me to test for HIV, he gave me a lot of information and support.

**[INTERVIEWER]:** you mentioned that he gave you a lot of information....can you please share with me the information that you received from him?

**[PARTICIPANT]:** we talked about many things concerning health, STI, HIV,

**[INTERVIEWER]:** what do you remember about risk behaviours from the talk you had with the peer educator?

**[PARTICIPANT]:** like group sex, unprotected group sex is very risky, and even if a one condoms will be used for two bottoms it's also risky. Even the people who inject drugs are at risk of HIV infection, we also talked about multiple partners as a risky behaviour too.

**[INTERVIEWER]:** what information did you received from the peer educator on oral self-testing?

**[PARTICIPANT]:** yeah, he gave me instructions of how to conduct the test as well as the benefits of the test especially the privacy bit. It is very confidential I will see my results alone.

**[INTERVIEWER]:** what information did you receive about acute HIV infection?

**[PARTICIPANT]:** he told me that Acute HIV infection is when you just got infected, and some symptoms will appear like malaria like symptoms fever, vomiting, body pains, sore throat. And at many times people self-treat themselves with pain killers or antibiotic because they assume it is malaria.

**[INTERVIEWER]:** when do these symptoms occur after HIV infection?

**[PARTICIPANT]:** they occur 2 weeks after HIV infection that what the peer educator told me.

**[INTERVIEWER]:** let me take you back to your testing experience, you mentioned that before you tested here the last time you did an HIV test was when you were going to [OVERSEAS_LOCATION] which was a year ago and that you came back within three months, did you experience the symptoms between the period of [RESEARCH_INSTITUTION]'s test and the test you had when going to [OVERSEAS_LOCATION]?

**[PARTICIPANT]:** Yes I did, I had similar symptoms around October/November

**[INTERVIEWER]:** when you had them, what did you do? Did you visit a health facility?

**[PARTICIPANT]:** I just took Panadol I bought them over the counter from a shop, I dint had the information about Acute HIV infection so I wasn't aware by then.

**[INTERVIEWER]:** you have shared that you didn't know about AHI until you met the peer educator, think about other information that you received from the peer educator, to what extent did you know about these before?

**[PARTICIPANT]:** before I met him I wasn't aware, I just knew about drug users and the risk of HIV infection.

**[INTERVIEWER]:** what can you say about the information you got from peer educator in terms of understanding?

**[PARTICIPANT]:** It was very clear and simple to understand, I understood everything that is why am able to remember up to now.

**[INTERVIEWER]:** yeah, I can see that (*both chuckling*)

**[INTERVIEWER]:** Did you receive any online material or resources?

**[PARTICIPANT]:** How, I don't understand

**[INTERVIEWER]:** I mean did they share any link that you can refer to for more information later, or did he gave you any information leaflet?

**[PARTICIPANT]:** Ooh, he didn't share any online material because I suspect they figured out I understood everything, but he gave me a leaflet on AHI.

**[INTERVIEWER]**: what positive thing can you say about the mobiliser and how he mobilised you?

**[PARTICIPANT]:** he gave me a lot of information he really took his time with me, he is very confidential I showed him the herpes zoster and have not heard that from anyone, since I joined here he has given me my free space he is not making curious follow ups like....did you test, what was your results, did you confirm it was herpes zoster, he hasn't asked me anything related to HIV after brining me to [RESEARCH_INSTITUTION].

**[INTERVIEWER]:** what negative thing can you say about the mobilizer and how they mobilized you?

**[PARTICIPANT]:** I don't want to lie am fasting I swear there is nothing negative from him he was just good.

**[INTERVIEWER]:** How do you think we can motivate GBT to take an HIV test?

**[PARTICIPANT]:** in my opinion I think RNA test can really encourage people to test, because most of us don't like to test regularly just to confirm if we are actually negative, so this RNA test might boost testing uptake.

**[INTERVIEWER]:** where did you learn about RNA test machine?

**[PARTICIPANT]:** I learnt it from the mobilizer too.

**[INTERVIEWER]:** seems like the mobilizer didn't leave anything (*both laughing*) so, let me take you back to oral self-testing, how was your experience like?

**[PARTICIPANT]:** I dint have any challenges using the kit the instructions were clear, but imagine I didn't view my result (*chuckling*)

**[INTERVIEWER]:** what were your reasons for not wanting to see the results, and what did you do with the kit that you had just used?

**[PARTICIPANT]**: I was very anxious and a little bit scared I think, I just put it back in the pack and waited for the counsellor to attend to me, so I went with it to him and told him that I haven't checked my results, so he empowered me to take it out and check the results together.

**[INTERVIEWER]:** what can you tell me about the counselling you received from the counsellor who tested you for HIV?

**[PARTICIPANT]:** the counselling was okay, before we viewed the results he asked me about possibilities of any results and we talked about it. When we took it out and found that it had two red bars, he counselled me and gave me information on HIV, it went on well.

**[INTERVIEWER]:** When did you start treatment after being diagnosed with HIV?

**[PARTICIPANT]:** I started after some days, I needed time to talk with myself on the long term treatment, taking daily pills without deciding from within is not easy.

**[INTERVIEWER]:** what can you tell me about the counselling you received about starting ARVs?

**[PARTICIPANT]:** Well, the counsellor told me about HIV and how it affects the body, importance of ARVs and benefits of early treatment, he said that early treatment has advantages of living a long healthy life and also lowers viral load and if they are low then I won't have any health challenges and also I won't be able to infect people.

**[INTERVIEWER]:** okay that was all about your testing experience and mobilization, now we will talk about your sex partners and partner notification services.

How was it introduced to you?

**[PARTICIPANT]:** After being tested I was introduced to him and we talked about my results and what they mean to me, he told me the importance of notifying partners and he gave me options on different ways of notifying them. The first one was issuing me an OST to give my partners, giving my partners contact detail so that they can be notified anonymously, involving the mobilizers and ask them to mobilize my partners I only remember those but one or two I have forgotten them.

**[INTERVIEWER]:** you mentioned that you discussed on the importance of notifying them, can you tell more about the importance?

**[PARTICIPANT]:** yeah, the importance is that when they are notified they will be linked to care depending on their HIV status i.e. the ones that will be positive like me will also start ARVs and this will reduce the chance of them infecting other people and living HIV positive without their knowledge and for those who will test negative they will be offered PrEP if willing and will be counselled on risk reduction.

**[INTERVIEWER]:** In your opinion, do you feel the same or have same views?

**[PARTICIPANT]:** Yes I feel the same, if only the one who infected me knew their HIV status before they infected me and I was notified and conducted an HIV test and have negative test result, perhaps I would be more careful and protected myself, or assuming that I was notified and founded to be HIV positive then I would have started ARVs and this herpes zoster won't have developed. But am not that late I thank God and the mobilizer and doctor, the doctor told me that it is on the beginning stage, it hasn't formed blisters it was only rashes he gave me drugs and rashes are beginning to dry up, I thank God.

**[INTERVIEWER]:** ok, so what option did you pick and what were your reasons for the same?

**[PARTICIPANT]:** Well... I opted for providing my partners details, because we discussed each strategy with its pros and cons, and for all strategies I think this was safe for me as I didn't want them to find out that am HIV positive.

**[INTERVIEWER]:** did you opted for this for every partner or for some partner you opted other strategies?

**[PARTICIPANT]:** I have told you that I didn't want any of them to find out about my HIV status so I opted it for all.

**[INTERVIEWER]:** did you discussed every partner individually and assess possible strategy for each?

**[PARTICIPANT]:** yeah we did and I thought giving their contacts will be the best way for me.

**[INTERVIEWER]:** how do you feel about the strategy now?

**[PARTICIPANT]:** hehehe to me it worked well, none of them have contacted me with HIV stories so none of them knew it was me am happy it was well.

**[INTERVIEWER]:** were your partners notified?

**[PARTICIPANT]**: yes, but I wish I was told about their test results so that I can know who infected me out of the four (*chuckling*)

**[INTERVIEWER]:** Allow me to ask you a curiosity question (*chuckling*), you mentioned that you opted for this strategy because you wanted your HIV status not be known by them, do you think it is fine for you wanting to know about their HIV status?

**[PARTICIPANT]:** (*laughing*) well, it is not fine and that is why I wasn't told, but you see I wanted to do my investigation and find out the ones that are positive so that I will know it is them who infected me

**[INTERVIEWER]:** ooh okay I get you now, would that have helped you in anyway like getting over about HIV status, denial.......

**[PARTICIPANT]:** (*interrupted*) yeah, if I get to know who tested positive, then I might have decide to self-disclose to them and settle down with them have a committed relationship because our results are the same and both of us will be on treatment and we might go bareback when we are viral suppressed.

**[INTERVIEWER]:** ooh, It is clear now, what you are saying is that it would have helped you in serosorting. Okay, now we will talk about your sex partners, do you remember how many partners you mentioned to be contacted?

**[PARTICIPANT]:** yeah I mentioned three partners and those are the ones that we had sex recently and it was unprotected.

**[INTERVIEWER]:** what was their gender?

**[PARTICIPANT]:** two were men and one female

**[INTERVIEWER]:** let us start with the female, if this person would be notified, how do you think she might react?

**[PARTICIPANT]:** I know this one will have issues and that is why I gave out their number...

*******Interruption, a knock on the door, someone left their handbag in the room and was asking for it. Opening and giving the bag back took 19 seconds *******

**[INTERVIEWER]:** Sorry for the interruption, there is a note on the door that an interview is taking place but the person needs the handbag because they are leaving for [CITY_C]. Let's continue, you were saying that you think the female partner will have issues right? What are your reasons for this?

**[PARTICIPANT]:** it's okay

**[PARTICIPANT]:** yes, she will have issues because I have heard rumours that she is HIV positive and likes young boys am not the only one having sex with her. I have been asking her several times to go with me for a couple HIV test but she always gets mad and tell me that I don't trust her and am listening to rumours. So I know definitely she will have issues with HIV test, I suspect she is the one who infected me, you remember I shared it with you too.

**[INTERVIEWER]:** Yes, I do remember you sharing that, so what do you think should be done to have her tested?

**[PARTICIPANT]:** Eeeh! Am not sure what can be done to her she is very complicated she doesn't want anything with HIV test, for now am not sure what can be done. Perhaps she needs a health talk face to face.

**[INTERVIEWER]:** Now let's focus on the two men and think about one, if this person was notified, how do you think he might have reacted?

**[PARTICIPANT]:** both the male have no issues, they have been peer educators at [ORGANIZATION_C] and have information about benefits of testing, so those ones have no issues I know they have come I was notified by the counsellor.

**[INTERVIEWER]:** With all the three partners, do you think the notifications have affected your relationship?

**[PARTICIPANT]:** No, because we have met with each one of them and nobody seemed to be suspicious with me so our relationship is the same.

**[INTERVIEWER]:** Okay, now we will discuss about disclosure of HIV status to others.

Have you disclosed your HIV status to anyone else?

**[PARTICIPANT]:** not yet.

**[INTERVIEWER]:** what are your reason/s for that?

**[PARTICIPANT]:** You know people out there are not informed about HIV, am not sure what will be their reaction, am afraid of being stigmatised and neglected, for now it's better I keep it to myself.

**[INTERVIEWER]:** you mentioned that, for now you would rather keep it for yourself meaning that it will reach a time when you will perhaps disclose this. If this time comes whom would you want to tell?

**[PARTICIPANT]:** Mmh, I told you am member at [CBO_A], but I have not yet disclosed my HIV status, you know that they have few negative MSM members so it's hard to tell if am positive or not. So, perhaps I would want to join the positive members support group and I will disclose to them because they can give me more moral support when I need it and they are experienced than me some have lived with HIV for many years.

**[INTERVIEWER]:** now we will discuss about estimating number of sex partners, to what extent was it possible to discuss with me all sex partners from the past 12 months in our first interview?

**[PARTICIPANT]:** Well, for me it was easy to remember and discuss, this is because these were people that I was meeting regularly and I didn't have other partners.

**[INTERVIEWER]:** Were there any other partners apart from the three that we did not discuss during our first interview?

**[PARTICIPANT]:** Uuuh... I think there is, but I didn't feel importance of them being notified because we had safe sex and beside I didn't had their contacts it was hit and run just one time.

**[INTERVIEWER]:** like how many? And what if they don't know their status do you think notification would have helped them learn their HIV status?

**[PARTICIPANT]:** mmh there are 4 others, 2 male and 2 females but as I told you I didn't mention them because I preferred giving partners contact details but unfortunately I don't have their cell phone numbers.

**[INTERVIEWER]:** okay thank you, we are almost through with the questions, now we will discuss about barriers and facilitators in offering partner notification service to GBT.

Could you tell us some of the ways that helped or could help you disclose or invite your partners?

**[PARTICIPANT]:** you mean the importance or?

**[INTERVIEWER]:** I mean the ways that could help you invite your partners

**[PARTICIPANT]:** oooh...it's hard for me that is why I picked the strategy I did, but I think one can just ask their partner to take them to some place then go with them to a health facility and then just ask them if they could visit a counsellor for questions and the counsellor will be pick from there.

**[INTERVIEWER]:** What do you think about partner notification services?

**[PARTICIPANT]:** it's really important and it is very helpful to partners as I had mentioned earlier. What I like most is there is no forcing people, strategies are discussed and the person is helped to pick the one they prefer most.

**[INTERVIEWER]:** what challenges do you foresee with PNS for GBT?

**[PARTICIPANT]:** well. It can make people suspecting a specific partner is the one who gave their numbers especially those who keep well track of number of their sex partners and what kind of risky behaviour they had. One who is keen can really suspect. It can also bring social issues like violence, rejection, stigma, losing financial support from partner.

**[INTERVIEWER]:** what do you think can facilitate uptake of partner notification services to GBT and have less challenges?

**[PARTICIPANT]:** just like the way you did it, discuss all the options available, and discuss each partner vs each strategy, pros and cons of each and them let them decide on the strategy they feel is safe.

**[INTERVIEWER]:** now we are on the last section of our questionnaire, it's all about practical aspect of partner's notification services implementation. Could you come up with communication strategies for health care provider to discuss PNS with partners?

**[PARTICIPANT]:** looking at all strategies all?

**[INTERVIEWER]:** yes, or you can discuss strategies that you are comfortable with in relation to communication

**[PARTICIPANT]:** okay, assuming I was given oral self-test kit to offer the partner, first I will start discussing about HIV and show him that HIV is not a big deal like before, once someone starts treatment they live a normal healthy life. I will communicate this without even making him suspicious that am targeting him. I will talk about all the positive things like how ARVs lower viral load and how some people become more beautiful with ARVs. Then I will tell them about OST kit and ask them to use it at their own free time.

**[INTERVIEWER]:** Now think about the HCP and the anonymous call, how would introduce it?

**[PARTICIPANT]:** (*chuckling*) I will start by greeting them so warmly on the phone, like asking how they are doing and mentioning their name to show that I know them. Then I will introduce myself as so and so and where I work, I will talk about other health related issues not necessarily mentioning HIV like the cholera outbreak. I will say that we have good machines for testing and invite them to come. Once they come then I will collect a sample and test for HIV *(Chuckling*)

**[INTERVIEWER]:** if am hearing you are right, you are saying that we should not mention HIV

**[PARTICIPANT]:** yes, calling someone and saying that you might have been exposed to HIV is a bit scary it's not an easy thing to the person you are talking to over the phone, I will just talk about general health and tests.

**[INTERVIEWER]:** assuming that, they indeed come to your facility because you mentioned general health and outbreaks, how would you then introduce HIV test?

**[PARTICIPANT]:** (*laughing*) I would just draw blood and test for HIV then later on tell them we started with HIV and this are your results and then shift the focus to HIV only. Just like how the pregnant women mandatory HIV test started, they were not consented for the test a sample was just taken and results provided.

**[INTERVIEWER]:** ok am getting your point, is there any other communication suggestion to another strategy?

**[PARTICIPANT]:** those two are the most sensitive ones as far as communication is concerned.

**[INTERVIEWER]:** Thank you very much, how much time after your HIV diagnosis was PNS discussed with you?

**[PARTICIPANT]:** it happened on the same day, but I feel people should be given time to process their new HIV status, getting HIV positive results is not easy. A counsellor can introduce PNS like

*"I would like you to go and think about PNS, it will be good you start thinking about the people you have had sex with recently, it is important to notify them. When we meet for the next appointment we will discuss deep about this"*

And the counsellor should not leave them alone, they should call patients often just to check on them, this will make them feel loved and respected and it will be easy for them to disclose number of sex partners openly.

**[INTERVIEWER]:** you just said for you it was discussed on the same day and now you mentioned that people should be given time, what is your feeling on this?

**[PARTICIPANT]:** for me it was easy because I was not emotional so it was just okay, I had already received information on HIV, people from [CBO_A] had already disclosed to me so I was fit for the same day. But I feel people should be given time not the same day chances of forgetting number of sex partners are very high.

**[INTERVIEWER]:** you talked about time, what time are you referring to and is the best according to your opinion?

**[PARTICIPANT]:** I think a period of maybe two to three days, and with the phone follow up those days are just enough.

**[INTERVIEWER]:** Thank you very much for your contribution, we have come to the end of our interview. Is there anything else you would like us to discuss about PNS and GBT?

**[PARTICIPANT]:** No, I have nothing else to discuss (*chuckling*)

**[INTERVIEWER]:** thank you very much for coming and dedicating your time for sharing your views and experiences, I truly appreciate.
