# Supplementary material for: Peer-assisted HIV partner notification services to strengthen index partner testing for newly diagnosed men who have sex with men in coastal Kenya
Source: PLoS One. 2025 Oct 7;20(10):e0333707. doi: 10.1371/journal.pone.0333707 (PMC12503256; doi:10.1371/journal.pone.0333707)
Supplement: S3 Appendix — (ZIP) [file pone.0333707.s003.zip › Deidentified IDI Transcript_1064.docx]

**Participant characteristics:**

Age: 45-49

Sexuality: Gay

Education level: Primary

Days between enrollment and IDI: 42 days

Mobilization strategy: OST

Final PNS Strategy: HCP/PM

**Partners identified: 1**

**[INTERVIEWER]:** The recorder is now on, Welcome again to today's discussion. Like I said earlier this study is called PNS where we notify partners of index patient so as they can also know their HIV status. Index patient is the newly diagnosed patient. There are different ways of partner notification, one is that you can choose to disclose your HIV status to your partner so that they can also get tested, there is another way where the health provider can make an anonymous phone call to the partner and invite them to the clinic for testing. Like I said we don't know exactly how to carry out PNS among MSM, Transwomen that's why we are carrying out this study, we want to learn more from you guys

**[PARTICIPANT]**: OK, that's good

**[INTERVIEWER]:** PNS is not entirely a new thing, it has been going on for a while now especially for the general population.

**[PARTICIPANT]:** Mmmm

**[INTERVIEWER]:** So, tell me, how are you doing since then.

**[PARTICIPANT]:** I have been doing well with my ART. I have now started using a condom as this was not my thing before, sometimes I even opt for masturbation as you know for one to get a woman sometimes you need to use money

**[INTERVIEWER]**: Since you last tested until this moment do you feel like you can now talk about your status or possibly disclose

**[PARTICIPANT]**: I don't think so, I am a little bit conscious, I had multiple partners and never used a condom at all, I can't even know where I got infected from and by whom. I still have some disturbing questions on the same, but I try to maintain my morale

**[INTERVIEWER]**: Have you possibly experienced any difficulty or even challenge?

**[PARTICIPANT]**: I only was afraid and got shocked when I first knew my HIV status, I didn't know what to do. I was full of thoughts and stressed all the time. Later things gradually got better

**[INTERVIEWER]:** mmmmh ok

**[PARTICIPANT]:** I had thoughts of like people may turn away from me, discriminate me

**[INTERVIEWER]:** I understand. What made you come for testing that day.

**[PARTICIPANT]:** There are some friends of mine who advised me to test. I woke up not feeling so well and decided to come to hospital then I remember what my friends had told me and decided to look for one of them who works here at the clinic.

**[INTERVIEWER]:** Mmmmm ok, what did the two of you talk about regarding testing, I mean your friend whom you were looking for

**[PARTICIPANT]**: He told me about a certain test that we used first, it was an OST test, we did that test and results were not very satisfying for me, they showed that I was HIV positive, afterwards we repeated the test again using the rapid tests. I was enrolled to care, and more blood sample was collected from me, I even had a certain urine test that was done.

**[INTERVIEWER]**: Ok, did he possibly explain to you on how to go about with the OST tests and possibly how to interpret the results on your own?

**[PARTICIPANT]**: Yes

**[INTERVIEWER]:** Can you remember exactly what you saw on the kit once the results were ready?

**[PARTICIPANT]**: Yes, I do, there were two red lines that appeared one was seen clearly and the other one was a faint line.

**[INTERVIEWER]**: Oooh OK, were the lines visible enough to read the results

**[PARTICIPANT]:** Ooh yes, as much as the line was faint but it was noticeable

**[INTERVIEWER]:** Were you given any IEC materials?

**[PARTICIPANT]**: No, I was not given anything

**[INTERVIEWER]:** ok, could you please share your experience on your encounter with our peer mobiliser?

**[PARTICIPANT]:** First of all, when he told me about testing, I felt insulted. In my mind I was like, "what could he have possibly seen in me for him to even talk about testing for HIV, is there anything wrong with me in my appearance, do I have maybe boils or something that he has seen in me for him to even tell me such a thing. Am I so immoral that he thinks there is a need for me to get tested?" I asked myself so many questions and even at some point I was thinking aloud of the same. He then said to me, "It's quite important to test for HIV for the sake of your health, its good to understand what is happening with your health so that you can take better care of yourself. I really appreciate the information he gave me, I can say generally was awakened after that conversation with him.

**[INTERVIEWER]:** Mmmh that's good to know

**[PARTICIPANT]**: Oh yes, now I know the importance of taking my drugs. My health is my number one priority. The thing is I have been avoiding my partners and even though if need be and I get the urge of having sex then I would use a condom. I don't like using a condom but that's just how it is now, or another option is masturbating

**[INTERVIEWER]:** That's amazing, see what the results of testing has done for you: now you say you are using condoms more often. That's good news

**[PARTICIPANT]**: Yes, I know, but its not really my cup of tea as I take longer to cum. I don't really feel satisfaction as compared to when I don't use a condom.

**[INTERVIEWER]:** But still we can say at least something positive came out of it. So, what do you think can be done to motivate more MSM, transwomen to get tested?

**[PARTICIPANT]**: Possibly when I meet with my fellow peers at the hotspots, I can talk to them about it. Even for those who would want to be in a relationship with me I would advise them we get tested first before anything else.

**[INTERVIEWER]**: Ok, when you went to enroll for care at [ORGANIZATION_A], were you given ART the same day?

**[PARTICIPANT]**: Yes, I was given DOT and later sent for Gene xpert test.

**[INTERVIEWER]:** The facilitation seemed amazing. Kindly share with me about the counselling you received. How was it?

**[PARTICIPANT]:** I was told to put lot of consideration on adherence to ART and even encouraged to use a condom always

**[INTERVIEWER]:** Do you feel this was helpful to you

**[PARTICIPANT]**: Yes, especially with adherence. I have witnessed HIV positive persons who did bot take their medication, the kind of condition they were in and surely don't want to find myself on the same position.

**[INTERVIEWER]**: What gave you that encouragement of taking those drugs?

**[PARTICIPANT]**: I am one person who commits in taking my medication to the latter whenever I am given a dose, probably that's what making it easy for me to take my medication

**[INTERVIEWER]:** Have you experienced any challenges ever since you started taking ART?

**[PARTICIPANT]**: Now I have appetite unlike before when my health started deteriorating.

**[INTERVIEWER]:** That's something positive that we should be grateful for. Now let's talk about your experiences with partner notification. Has this affected your relationship with your partners?

**[PARTICIPANT]:** There is one that we not talking anymore. Nowadays she doesn't want anything to do with me

**[INTERVIEWER]**: You talking about the female partner, right?

**[PARTICIPANT]**: Yes

**[INTERVIEWER]:** I remember that there was no any contact information about her

**[PARTICIPANT]**: Yes, I didn't

**[INTERVIEWER]:** Therefore, we need to talk more about her and possibly give us information that will help us to get in touch with her

**[PARTICIPANT]**: Most definitely, afterwards I will go find her phone number then will come to give it to you. Talk to her in a way you know best without involving me at all

**[INTERVIEWER]**: That's not a problem, rest assured that your information is safe with me. So, during introduction of partner notification we explained different ways that can be used to reach your partners, do you have any information whether your partners were able to be contacted and came to the clinic for testing?

**[PARTICIPANT]:** That's where I say that its hard really because mostly when someone hears this kind of thing, they get shocked for sure.

**[INTERVIEWER]**: Sorry, allow me to take you back a little bit. There is this partner that you talked about to my colleague and gave him contact information of the partner, do you know whether he finally came to the clinic.

**[PARTICIPANT]:** Not really, its been a while since I talked to him, I believe the last time we got in touch was before Ramadhan

**[INTERVIEWER]**: Is there any other that probably told you that they received a phone call from the clinic inviting them for HIV testing

**[PARTICIPANT]**: No

**[INTERVIEWER]**: What made you not invite him for testing yourself but rather opted for my colleague to follow him up?

**[PARTICIPANT]**: He gets angry really fast so I thought that when a counsellor who has skills approaches him, he may consider and agree to come for testing, otherwise he could even insult me for it.

**[INTERVIEWER]**: Ok, we are still on the issue of the sexual partners you mentioned to us on the day of enrollment. There are others that we were not able to reach ...

**[PARTICIPANT]:** Yes, there is this Mzungu who doesn't come from around, he only comes to visit and goes back. He doesn't even say when he will be back, I only receive a phone call from him when he is already settled in a hotel

**[INTERVIEWER]:** Is the mzungu male or female?

**[PARTICIPANT]**: Male

**[INTERVIEWER]:** When was the last time you had sex with him?

**[PARTICIPANT]**: Its very long time ago, 2years

**[INTERVIEWER]**: Oh, ok. So, we are left with the female partner, you mentioned earlier that you will come back with her number soon so that we can try and get in touch with her. You also said that right now your relationship with her is a bit shaky...

**[PARTICIPANT]:** Yes, we had even planned to get together for Eid celebrations, but she suddenly changed and her behavior toward me is not satisfying at all.

**[INTERVIEWER]**: Do you think she will agree to come for testing when we finally reach her?

**[PARTICIPANT]**: Yes, she will. She used to encourage me to come for testing back then so I don't think she will refuse to come for testing herself.

**[INTERVIEWER]**: Mmmh...therefore we were able to get one of your partners whom you discussed with our colleague and finally came for testing. Has there been any change in any of your relationships so far? What I mean is has there been any change in your relationships ever since you knew YOUR HIV status?

**[PARTICIPANT]:** I am not nervous anymore like the first time. In the beginning it was always in my mind and made me stress over it most of the time but after a while I gathered myself up and told myself that I need to move forward instead of having a heartache over the situation. I know people who are living with HIV and they are doing ok so why not me.

**[INTERVIEWER]:** That's the way to go, our health should matter most. Therefore, we discussed about your partners and that one of them was able to come to the clinic. Has there been any change in your relationships so far?

**[PARTICIPANT]:** Not really, only what I have discussed with you about my female partner whom things are not quite the same now

**[INTERVIEWER]:** Ok. There is also a male partner that you discussed with us, how is your relationship so far?

**[PARTICIPANT]:** You know those are capable, they have money, I didn't have sex with them because I wanted to, but they paid me, I don't expect anything to change as they can get another person as well pay him and do the same thing.

**[INTERVIEWER]**: Ok, so apart from the three partners you talked about ie. The male partner in [CITY_B], the female partner and the mzungu partner who frequently comes to visit though this one it's been like two years since the two of you were together. Is there any other that you could possibly have forgotten to mention?

**[PARTICIPANT]:** No, just those ones.

**[INTERVIEWER]:** Ok, now we will talk about your condition. Have you ever disclosed to anyone about your status?

**[PARTICIPANT]**: No, I only talked to my mother about it. She was shocked about it and worried at the same time, but I told her it was ok we can cry over spilt milk what is left for me is to stick to my medication

**[INTERVIEWER]**: What really pushed you to tell your mother about it.

**[PARTICIPANT]**: She is my mother, she gave birth to me. I can't hide anything from her. If things get to worse right now, I know she is the one who will take care of me, therefore someone like that deserves to know about my condition

**[INTERVIEWER]:** Ok. So how is your relationship so far with your mother ever since you disclosed your status to her

**[PARTICIPANT]:** My mother is not happy with me. The friendship we had is not the same anymore

**[INTERVIEWER]:** What do you think could have caused hitches in your friendship, do you think its because she knows you are HIV infected or is there something else that could have made your friendship go sour a little bit.

**[PARTICIPANT]:** She used to warn me before about my ways. I introduced many women to her, and she even insisted that I get married and settle down cause my behavior was getting out of hand, but I didn't listen. All I did was making empty promises to her all the time.

**[INTERVIEWER]**: Is there a possibility that she knows you are an MSM?

**[PARTICIPANT]**: No, she doesn't know that.

**[INTERVIEWER]**: Ok, lets go back to partner notification. Do you remember the first time you discussed about your sexual partners, was there in difficulty in doing so?

**[PARTICIPANT]**: Nope, because I feel that's just normal. Relationships are not anything new at all

**[INTERVIEWER]:** So, there was no any difficulty at all

**[PARTICIPANT]:** Not really

**[INTERVIEWER]**: And were you able to identify all the partners that you had sex with in the last one year?

**[PARTICIPANT]**: yes of course

**[INTERVIEWER]:** Apart from the three you mentioned have you had any new relationships?

**[PARTICIPANT]**: No, I haven't had any new relationships

**[INTERVIEWER]**: Could you please identify other ways that you feel can be used to notify partners?

**[PARTICIPANT]:** I think what I can do is only disclose my own status to the partner then come together to the clinic for more counselling

**[INTERVIEWER]**: Ok, PNS now is a service offered to anyone who tests HIV positive so that their partners could also get tested and know their status, how do you feel about this?

**[PARTICIPANT]:** Its not a bad thing because when you know each other as in that both of you are HIV positive then you will be having sex with your fellow partner who is just as infected as you are. This can reduce HIV transmission instead.

**[INTERVIEWER]**: Can you recommend PNS to others as well?

**[PARTICIPANT]:** Yes, I would.

**[INTERVIEWER]**: Why?

**[PARTICIPANT]:** I may get a woman who loves me but before anything I would ask her to come and get tested with me.

**[INTERVIEWER]:** As I said earlier PNS is now incorporated as part of the services offered at the VCT. This was done for everyone, but we have no idea of how to make PNS effective to MSM, gay, Transgender. Do you have any ideas on how we can do this for this group of people?

**[PARTICIPANT]**: I believe now its up to the homosexuals to tell their partners to get tested. They should encourage couple testing always

**[INTERVIEWER]:** What you are trying to say is that you should test together before having any sexual relationship?

**[PARTICIPANT]**: Yes

**[INTERVIEWER]:** Do you think there will be any challenges in delivering this service to MSM, GAY or even Transgender?

**[PARTICIPANT]:** I don't think there will be any challenges since we will both be infected therefore no one will be able to question the other person.

**[INTERVIEWER]:** It doesn't always have to be that both of you are HIV infected, it is also possible that one of you could be negative while the other is infected

**[PARTICIPANT]:** Yeah that's possible and if its so then the decision is up to them.

**[INTERVIEWER]:** Ok, what do you think can be done to make PNS successful to MSM, gay OR even Transgender

**[PARTICIPANT]:** aaaaaah... You know people are different others can react positively to HIV positive results and others can be quite shocked. So, I believe it really depends.

**[INTERVIEWER]:** Do you think notifying partners has any benefits at all?

**[PARTICIPANT]:** Yes of course, the partner will be able to get tested and know their status. For those who are HIV positive then they will benefit from ARV's

**[INTERVIEWER]**: Ok like we discussed earlier we have different ways of Partner notification. We have the index patient, peer educator and the counsellor. Therefore, the counsellor can notify the partner by making an anonymous phone call to the partner, we have the peer educator who can give the partner OST kit so that they can test themselves. Another way is that the peer educator can give the index patient an OST so that they can give their partner to test on their own, the counselor can also help the index patient to convince the partner to test for HIV. Another one is that the peer educator can also help the index patient to notify their partner. Therefore, we have different ways of notifying the partner. What do you think of these ways that we just discussed?

**[PARTICIPANT]**: I have done this I believe, I was once given an OST test and did the same. I tested with the kit then afterwards did a confirmation with the rapid test

**[INTERVIEWER]:** Among these ways that we discussed of partner notification, which one would you go for

**[PARTICIPANT]**: For me I think I that I would come with my partner for couple testing so that we can both know our status together.

**[INTERVIEWER]:** Is there any other way apart from the ones mentioned that you would have used.

**[PARTICIPANT]**: Another way I would use is just the one you mentioned, giving you contact information of the partner then you can make an anonymous phone call to the partner and invite them for testing.

**[INTERVIEWER]:** Ok, how long did it take for the counselor to discuss about the partners with you?

**[PARTICIPANT]**: It took a long while I even told him that the environment is not conducive for us to talk about such issues, I decided to come to the hospital instead.

**[INTERVIEWER]:** Ok, what I mean is how long did it take after you knew your status for the counsellor to discuss with you about your partners.

**[PARTICIPANT]**: He didn't take long, we immediately discussed about my partners and gave out contact information for my partners so that they can make an anonymous phone call to them and ask them to come for testing. There is only this female partner whom I am yet to give you her phone, but I will do it as soon as possible.

**[INTERVIEWER]**: Did the discussion take place the same day?

**[PARTICIPANT]**: Yes

**[INTERVIEWER]:** Do you feel the that it was too sudden to discuss about your partners and rather have given you more time and have the discussion afterwards.

**[PARTICIPANT]:** No, it was ok for me as I know all my partners. That was not a problem at all

**[INTERVIEWER]**: Is there a better way or maybe have the right words that you feel can be used that would make the partner agree to come for testing

**[PARTICIPANT]:** You can just ask her like, "hey there is this person that you once asked to go for testing for HIV with, how is it so far. Then I would ask her to come for testing, she will agree and once we come here you will assume that its just a normal couple testing

**[INTERVIEWER]**: Ok, so what if I was making that phone call now, what would you prefer I tell them?

**[PARTICIPANT]**: You can just say there Is someone here who has come for testing and they would like it if you tested together, they would probably want to know who that person is and when you tell her its me then she would agree to come.

**[INTERVIEWER]**: So, are you saying its ok to mention your name when making that phone call?

**[PARTICIPANT]**: Now she will think that you are my woman because she is so jealous. That's possible to happen you never know

**[INTERVIEWER]**: Mmmh, Ok. We know that its rather difficult for one to open up and discuss about their sexual partner, you need to gain trust for them to believe in you and feel that the information they are giving out is quite safe as well is there identity. What do you think we can do so as to gain that trust so that they can willingly be able to discuss about their sexual partners just like you did?

**[PARTICIPANT]:** It will take time

**[INTERVIEWER]:** so, what you are saying is that sometime the index would not want to discuss about their sexual partner to a stranger, you need to have a good relationship with them first.

**[PARTICIPANT]**: Yes, you can even befriend them, and they would eventually feel free and talk about their partners with you

**[INTERVIEWER]**: This means that PNS is ongoing, you shouldn't give up with the first attempt, but we can talk about it again whenever the index comes to the clinic or even make a phone call just to check up on them.

**[PARTICIPANT]:** Yes, this will make them feel that at least someone cares and it's just not about getting the information about their sexual partners but its rather important for the partners to also get tested and benefit from either ART or otherwise as the counsellor would advise.

**[INTERVIEWER]**: wow, that's quite informative. Do you have something to say about PNS In regard to MSM, Gay or even Transgender. Any additions or even criticism.

**[PARTICIPANT]:** No, just to assure you that I will find the phone number of my female partner and bring it to you myself. I would really love it if you find her and if possible, I also get to test with her as well.

**[INTERVIEWER]**: Ok, thank you very much for your time. We have come to the end of today's discussion, thank you again for taking your time to take part in this study and come here today. We appreciate you a lot.
