# Supplementary material for: Peer-assisted HIV partner notification services to strengthen index partner testing for newly diagnosed men who have sex with men in coastal Kenya
Source: PLoS One. 2025 Oct 7;20(10):e0333707. doi: 10.1371/journal.pone.0333707 (PMC12503256; doi:10.1371/journal.pone.0333707)
Supplement: S3 Appendix — (ZIP) [file pone.0333707.s003.zip › Deidentified IDI Transcript_1072.docx]

**Participant characteristics:**

Age: 30-34

Sexuality: Gay

Education level: Secondary

Days between enrollment and IDI: 106 days

Mobilization strategy: AHI

Final PNS Strategy: Index

**Partners identified: 0**

**[INTERVIEWER]:** welcome to this interview, I will be recording this interview as I briefed you earlier so as to capture your opinions. Feel free and thank you for allowing this interview to be recorded. This interview is being conduct on [DATE]...now I'd like to start off this interview with my first question, how are you?

**[PARTICIPANT]:** am fine and I don't seem to have any problem as of now

**[INTERVIEWER]:** when you said you don't have a problem as of now does it mean at some point you did have some troubles?

**[PARTICIPANT]:** not really, I've been ok.

**[INTERVIEWER]:** when you came to know about your HIV status how did you feel?

**[PARTICIPANT]:** I came to terms with the results and lived my life well.

**[INTERVIEWER]:** did you accept your status immediately after knowing..?

**[PARTICIPANT]:** it wasn't immediately but eventually I did accept myself.

**[INTERVIEWER]:** what sort of challenges did you go through?

**[PARTICIPANT]:** I just thought how I'd live and how life would be for me...

**[INTERVIEWER]:** so what eased your worries?

**[PARTICIPANT]:** I found comfort from counselling and I came to terms with the situation.

**[INTERVIEWER]:** what drove you to come for testing?

**[PARTICIPANT]:** I just wanted to know about my status.

**[INTERVIEWER]:** what drove you to that decision, of all days why that day?

**[PARTICIPANT]:** I had a boil around my anal region so I was worried and I decided to go for testing.

**[INTERVIEWER]:** so you were worried?

**[PARTICIPANT]:** yeah.

**[INTERVIEWER]:** How was your testing experience before the last HIV test you took here?

**[PARTICIPANT]:** I usually test occasionally when at my work place

**[INTERVIEWER]:** I understand you were introduced here by a mobilizer, let's talk about your mobilizer...how did he/she approach you?

**[PARTICIPANT]:** the mobilizer just approached me and educated me about the symptoms and risks of HIV/AIDS.

**[INTERVIEWER]:** did the mobilizer tell you anything about behaviours that can lead to such too? And what did the mobilizer say?

**[PARTICIPANT]:** yes, the mobilizer did talk about that too like having intercourse without protection, group sex and...I don't recall some.

**[INTERVIEWER]:** did the mobilizer tell you about the kit or did you come to know about it on your own?

**[PARTICIPANT]:** the mobilizer did tell me about oral testing, it's a tool you can conduct a self-test and it is confidential because you get to see your results first hand and it is very easy to use.

**[INTERVIEWER]:** did the mobilizer tell you about acute HIV infection?

**[PARTICIPANT]:** yes, the mobilizer did.

**[INTERVIEWER]:** can you tell me more about AHI

**[PARTICIPANT]:** it's getting infected by a new HIV virus...

**[INTERVIEWER]:** are you saying that some viruses are old and some new?

**[PARTICIPANT]:** no...like the symptoms are visible quite sooner

**[INTERVIEWER]:** ooh, you've talked about symptoms...what sort of symptoms are you talking about?

**[PARTICIPANT]:** body aching, headache and diarrhea, fever... I can remember those

**[INTERVIEWER]:** when do these symptoms show up?

**[PARTICIPANT]:** roughly about two weeks after HIV infection

**[INTERVIEWER]:** was it easy for you to understand such information? About the OST, AHI and behaviors that may lead to infection.

**[PARTICIPANT]:** it was neither easy the mobilizer explained things so well to me.

**[INTERVIEWER]:** did you know anything about these information before the mobilizer approached you?

**[PARTICIPANT]:** I did know about some...

**[INTERVIEWER]:** what did you know of and what did you not know of?

**[PARTICIPANT]:** I knew about how HIV could be transmitted sexually but had no idea about AHI.

**[INTERVIEWER]:** what about OST? Was this new news to you or you were aware of it?

**[PARTICIPANT]:** I had heard of it but didn't know how it looked like or how it is used.

**[INTERVIEWER]:** did the mobilizer share any sort of resources with you? A booklet or a flier?

**[PARTICIPANT]:** no.

**[INTERVIEWER]:** how was your experience with the mobilizer? The approach and the teachings, how would you sum them up?

**[PARTICIPANT]:** well, the mobilizer did help me know so much that I wasn't aware of and now I have better knowledge on somethings I never had heard of and now I can educate someone else too

**[INTERVIEWER]:** so it was a learning experience for you?

**[PARTICIPANT]:** yeah.

**[INTERVIEWER]:** what did the mobilizer do to impress you?

**[PARTICIPANT]:** educating me about AHI, to me it was really helpful and useful.

**[INTERVIEWER]:** what do you think the mobilizer didn't do or should have done better?

**[PARTICIPANT]:** being a mobilizer is a voluntary work, to be able to mobilize people is a challenge...

**[INTERVIEWER]:** I can't quite understand clearly, what should the mobilizer do or should have done to improve?

**[PARTICIPANT]:** what I think the mobilizers should do is after mobilizing they should make a follow up and ensure that the clients have gone for testing or it would be zero work for them.

**[INTERVIEWER]:** what do you think we should do to encourage people at risk of being infected to be test regularly?

**[PARTICIPANT]:** everyone should feel responsible and get to know of their status, mobilizers should also make a follow up on their clients and ensure they get the services required.

**[INTERVIEWER]:** mobilizers aside, you as an individual how do you think we can reach out to people with the risk of getting infected with HIV?

**[PARTICIPANT]:** well there are various ways to approach that, we can use social media pages like Facebook and Instagram, friends and talk to partners and help each other out.

**[INTERVIEWER]:** tell me more about using the Facebook and Instagram pages

**[PARTICIPANT]:** we have many gay groups on both social media platforms, if we have an individual account or a page that can post and promote HIV testing then a few people will inbox to inquire for me and will be directed to come for testing

**[INTERVIEWER]:** what was your experience when using OST? Were there any challenges?

**[PARTICIPANT]:** no, none it was easy for me to use it because it had a manual instructing of how to use the kit.

**[INTERVIEWER]:** I'd want you to be audible so the recorder can capture everything clearly please, did you start ARV's treatment as soon as you realized you were infected?

**[PARTICIPANT]:** after knowing of my status I did start ARV's treatment the following day.

**[INTERVIEWER]:** so you started the following day after testing?

**[PARTICIPANT]:** yes

**[INTERVIEWER]:** what made you not to start the same day?

**[PARTICIPANT]:** working hours were already over so I had to come the next day.

**[INTERVIEWER]:** what was it like coming for the ARV's? How did you feel?

**[PARTICIPANT]:** i just didn't think I could manage taking pills every single day.

**[INTERVIEWER]:** how about now...how's your adherence with taking the medication? Have you sometimes skipped taking your pills? Have your fears been proven right?

**[INTERVIEWER]:** no, I have managed to fight off my fears, firstly it was hard but now am used to it.

**[INTERVIEWER]:** how was it hard at the beginning?

**[PARTICIPANT]:** not everyone likes taking pills every day, so to adapt to that takes time.

**[INTERVIEWER]:** what motivated you to be taking your pills daily?

**[PARTICIPANT]:** it is very important according to the doctor's advice so I didn't want to miss them for the betterment of my health too.

**[INTERVIEWER]:** what were you advised about taking your pills?

**[PARTICIPANT]:** I was told how important it was to take the pills on a daily basis because it will boost my immunity.

**[INTERVIEWER]:** Okay...

**[PARTICIPANT]:** let's say instead of pills there should be a form of injection where one can come for an injection that would last up to three months and so on and so forth instead of moving around with pills everywhere if it is possible though...

**[INTERVIEWER]:** it can be possible, there are researches that aim making that happen and when they come to pass probably we will offer such services too. Let's proceed to my next question, I want to ask about your sex partners and PNS...how was PNS introduced to you? After testing you came the following day for your ARV's, how was that topic introduced to you?

**[PARTICIPANT]:** I met a counsellor and I was introduced to PNS and the importance of it.

**[INTERVIEWER]:** ok, what can you say about the importance of notifying your partner? After the counsellor told you about the importance of notifying your partner how did you perceive it?

**[PARTICIPANT]:** well it is a good thing, to sit and talk to your partner and understand each other well and you notify your partner so as to know each other' status and be able to take safe measures

**[INTERVIEWER]:** what strategy did you use to notify you're partner?

**[PARTICIPANT]:** pardon?

**[INTERVIEWER]:** do you remember the strategies used to notify your partner?

**[PARTICIPANT]:** there was a kit I was given...

**[INTERVIEWER]:** given or were you provided with options to pick?

**[PARTICIPANT]:** yes I decided to take the kit and talked to my partner and gave the kit to my partner to test.

**[INTERVIEWER]:** any other method? Did you give your partners number maybe for a mobilizer to call them?

**[PARTICIPANT]:** no I didn't I decided to take the kit to my partner:

**[INTERVIEWER]:** what made you to choose that option?

**[PARTICIPANT]:** to involve mobilizer is more like not being confidential with you and you're partner's matters, so I preferred a direct approach because I know my partner best and a third party to them is like exposing our matters to outsiders.

**[INTERVIEWER]:** was that strategy was helpful to you?

**[PARTICIPANT]:** yes it was.

**[INTERVIEWER]:** and what's your say on that strategy?

**[PARTICIPANT]:** personally I think it is the most preferable strategy, not all people are pleased with being contacted by a stranger and be told about their health issues.

**[INTERVIEWER]:** let's shift to our next question. How many partners did you list?

**[PARTICIPANT]:** just one.

**[INTERVIEWER]:** so how was your partner's reaction as in how did you even begin to explain things to your partner and how hard was it for him to understand and accept using the kit?

**[PARTICIPANT]:** I explained things to him as things were told to me by the mobilizer and my partner finally agreed to use the kit.

**[INTERVIEWER]:** what were the results?

**[PARTICIPANT]:** positive.

**[INTERVIEWER]:** has your partner started treatment?

**[PARTICIPANT]:** my partner started treatment immediately after that day.

**[INTERVIEWER]:** where does your partner take his pills from?

**[PARTICIPANT]:** [HOSPITAL_B]

**[INTERVIEWER]:** so you had no problem talking to your partner about testing at all?

**[PARTICIPANT]:** none at all.

**[INTERVIEWER]:** and has this in any way affected your relationship?

**[PARTICIPANT]:** no.

**[INTERVIEWER]:** so you two are still together to this date?

**[PARTICIPANT]:** yes.

**[INTERVIEWER]:** ok, now we'll talk about disclosing your status to others, have you told anyone about your current status?

**[PARTICIPANT]:** I haven't disclosed it to anyone, personally I think one's health concerns are a personal matter and no one should actually know a thing.

**[INTERVIEWER]:** so that's the only reason why you haven't disclosed your status to anyone?

**[PARTICIPANT]:** yes.

**[INTERVIEWER]:** let me ask you about your sex partner, you did disclose to him...why did you only confine in him about your status?

**[PARTICIPANT]:** according to the counsellor HIV/AIDS has branches, might be number five or number ten but if you have unprotected sex you are way out of those numbers so it's a different type OF AIDS so I had to be open with my partner because we share a sexual relationship.

**[INTERVIEWER]:** and that hasn't affected your relationship at all?

**[PARTICIPANT]:** yes.

**[INTERVIEWER]:** now let's talk about safety of partner's notifications, did you face any troubles or security problems when you opened up to your partner?

**[PARTICIPANT]:** as for me and my partner I did not experience any sort of threat or harm, my partner is very understanding and an open minded person, I had no fears when I approached him because I know him.

**[INTERVIEWER]:** ok, let's now talk about partner's estimation, can you recall how many partners you were involved with sexually in the twelve months?

**[PARTICIPANT]:** it was easy to recall because I do not have too many partners, I stick to one partner till when things don't work out for us.

**[INTERVIEWER]:** you just listed one partner when you came for testing, was he the only one you were sexually involved with in the past twelve months?

**[PARTICIPANT]:** yeah, it was just him.

**[INTERVIEWER]:** do you think it is easy for someone to recall their sexual partners in the past twelve years?

**[PARTICIPANT]:** maybe, people are different, some it's their business and some just seek pleasure in one person.

**[INTERVIEWER]:** do you think the ones who do it as a business can recall their numbers?

**[PARTICIPANT]:** not really, some might miss clients in certain days plus they really don't see the need to keep records so it's hard to tell.

**[INTERVIEWER]:** was there may be a partner you left out or forgot to mention when you were asked before?

**[PARTICIPANT]:** no.

**[INTERVIEWER]:** ok, to our next topic, barriers and facilitators in offering services to GBMT, can you suggest or have an opinion on how we are going to render services to GBMT?

**[PARTICIPANT]:** it's a challenge, cause it's a voluntary act, some people have an attitude and vow to never go for testing. I think the best thing I think is using the person who came for testing willing.

**[INTERVIEWER]:** what if that person is afraid because they do not have a strong relationship with other partners? Some just meet once a month or even once in every three month and they might be threatened by their partners.

**[PARTICIPANT]:** I think what you can do is just seek the people the mobilizer can mobilize and with them it will be a chain, those people at risk might reveal other partners too.

**[INTERVIEWER]:** apart from mobilizers how do you think we can approach this matter? It is important to have an alternative and not rely on just one method.

**[PARTICIPANT]:** maybe hold a forum and pass information to the public and maybe some people in that crowd who might be touched and be willing to come forth and may use them to get valuable information.

**[INTERVIEWER]:** tell me about this forum?

**[PARTICIPANT]:** well, maybe it might be make an announcement in a hospital institution that there will be an event and that they would be an educational forum on a certain date and that the topic is going to be specific because and then you won't just get GBMT but with the forum you might just get some GBMT.

**[INTERVIEWER]:** with that you can't get the partners of your clients because some might not even be at the hospital at all or they might not be interested at all, so how can this help?

**[PARTICIPANT]:** you might tell your partners to go to that event and ask them to go to that place, when there after listening to the forum I can ask my partner to go for testing with me, I already know of my status so I will be confident for the sake of my partner so as to make them accompany me too.

**[INTERVIEWER]:** what about PNS? What's your say on that, do you think there's need to notify partners?

**[PARTICIPANT]:** as I said before it's a voluntary act, not everyone can just tell their spouses about this, some have wives and might take the news differently, so I suggest people take their partners for testing.

**[INTERVIEWER]:** you said that one should come with their partner for testing right?

**[PARTICIPANT]:** before testing the person must be accompanied by their partner.

**[INTERVIEWER]:** ok. You said some have more than one partners, how would this apply? Should that person come with all the partners for testing?

**[PARTICIPANT]:** yes, but you must have your main partner, so with your main partner you ask him to go for testing.

**[INTERVIEWER]:** well, how about those who just have many partners but don't have a main, they do it for business how do we get those people to come with their partners?

**[PARTICIPANT]:** it won't be easy that's why you should conduct those forums and maybe out of ten partners three must show up.

**[INTERVIEWER]:** back to your statement that it's not advisable to notify your partners, why aren't you recommending that?

**[PARTICIPANT]:** like I said earlier it is something voluntary act, after counselling you might see that the counselor is concerned and maybe you might volunteer to help put and have your partners over too, but if they come willingly I think is it is a good idea, like after testing I just encouraged my partner we go testing and if they willingly agree they won't be that much shocked because it's a test conducted by the doctor.

**[INTERVIEWER]:** correct me if am wrong but from what I understand is you don't have a problem with PNS but what you prefer is the partner who came for testing to personally notify the other partner and ask the partner to go for testing with them, am I right?

**[PARTICIPANT]:** if it's possible before testing the partner you recommend that they be accompanied by their partners too, but if it's a partner with many partners and their partners are approachable then the idea of the forum can be applicable.

**[INTERVIEWER]:** so you are against mobilizers calling partners?

**[PARTICIPANT]:** I love a private life so I won't be comfortable someone else giving out my number and maybe someone might have some bad intentions and call you to meet up with them and just to harm you. Personally I love a personal life and wouldn't want anyone I don't know have access of my number.

**[INTERVIEWER]:** ok, so you are not against PNS but against giving out people's contacts, would you recommend that we educate people about PNS?

**[PARTICIPANT]:** according to me after educating someone about PNS someone might like have an attitude and question the doctor, as a grown up when you find out about your status you have the will to decide what's right for you, you can ask your partner to come for testing or take a kit to them. But it is important to educate them about PNS.

**[INTERVIEWER]:** what do you think are the advantages of PNS?

**[PARTICIPANT]:** yes it has some advantages when you notify your partner and plan on how to live your lives responsibly.

**[INTERVIEWER]:** after notifying your partner and they test positive how will this impact a relationship?

**[PARTICIPANT]:** it does impact a relationship, if the partner is an understandable being you will plan out your lives together.

**[INTERVIEWER]:** what if they test negative? How will this impact your relationship?

**[PARTICIPANT]:** well you will explain things clearly to them and tell them that you are already under medication and that your viral load is low and it won't be able to affect them.

**[INTERVIEWER]:** now to our last questions, the duration that took you to be educated about PNS and asked to go for testing do you think it was rushed?

**[PARTICIPANT]:** no, it's best to know your status earlier.

**[INTERVIEWER]:** what am asking is after testing do you think it was too early to be educated PNS or was there need for it to be introduced before?

**[PARTICIPANT]:** it was very fast, after knowing of your status you need time to come to terms with everything then all can be introduced later on.

**[INTERVIEWER]:** how long do you think one should be introduced to this new information?

**[PARTICIPANT]:** three weeks to four months.

**[INTERVIEWER]:** don't you think it might be risky for the partner, it might spread to other partners? The logic of PNS is to be able to notify your partner so they can test and know of their status so as to avoid spreading it further too and three weeks to four months is such a long time?

**[PARTICIPANT]:** yeah, that is true, well I think it's not supposed to be an immediate thing, after testing you do have their details and can contact them after a day or two and ask them to meet up and introduce them to PNS because immediately after testing some may not even be able to concentrate and understand what is said to them. At least five to seven days.

**[INTERVIEWER]:** I know you are against this strategy, giving out your number to mobilizers, assume it's the only strategy, I know it is very challenging and people ask how you were able to obtain their contacts and ask so many questions, so what do you think can be done or what sort of message do you think should be packed to try and make people understand? Assuming we are calling your partner what do you think we should say during the call?

**[PARTICIPANT]:** most men are easily lured by women, and if a man calls another man there might be a misunderstanding but if a woman calls a man they can lure them with words...

**[INTERVIEWER]:** what words exactly?

**[PARTICIPANT]:** it should start by introduction and a meeting place and I don't think if the mobilizer should say what center they work with. But there would still be a challenge because some would still ask how you get their phone numbers.

**[INTERVIEWER]:** so you are suggesting that we shouldn't be too formal and mention our work place?

**[PARTICIPANT]:** yes, it scares some people and they would be so furious.

**[INTERVIEWER]:** so you are totally against us telling them that where we work and what why we called them?

**[PARTICIPANT]:** yeah.

**[INTERVIEWER]:** ok, we are almost done now, what do you think we should do to build trust and confidentiality to the person who just tested? When discussing about notifying their partners.

**[PARTICIPANT]:** how am I going to trust you people to keep it confidential?

**[INTERVIEWER]:** yes.

**[PARTICIPANT]:** it's a bit challenging but after giving you the number I would live with the fear of maybe you would tell on me.

**[INTERVIEWER]:** we never disclose the names, some demand to know who it is s they would show up but we never tell them. We even ask them to go for testing at some different center not specifically ours.

**[PARTICIPANT]:** what I think is when you call I should be present so as to hear the entire conversation.

**[INTERVIEWER]:** Is there anything you recommend we change or improve on our PNS strategies?

**[PARTICIPANT]:** I really think your methods are quite useful and I don't see the need to add on anything relating to that matter, and when sending mobilizers at least they should be straight men so they don't get judged or even get beaten up by the community.

**[INTERVIEWER]:** that's a nice point, anything else?

**[PARTICIPANT]:** well, when someone is seeing coming in to your center they are judged to be GBMT so what am suggesting is people walking in at least they act manly so as the community to not be too keen on what sort of people come in and out of your center.

**[INTERVIEWER]:** thank you for allowing me to record this interview, thank you for sharing your opinions with me and thank you for your time.
