# Supplementary material for: Peer-assisted HIV partner notification services to strengthen index partner testing for newly diagnosed men who have sex with men in coastal Kenya
Source: PLoS One. 2025 Oct 7;20(10):e0333707. doi: 10.1371/journal.pone.0333707 (PMC12503256; doi:10.1371/journal.pone.0333707)
Supplement: S3 Appendix — (ZIP) [file pone.0333707.s003.zip › Deidentified IDI Transcript_1113.docx]

**Participant characteristics:**

Age: 20-24

Sexuality: Gay

Education level: Primary

Days between enrollment and IDI: 43 days

Mobilization strategy: Open B

Final PNS Strategy: Index

**Partners identified: 3**

**[INTERVIEWER]:** Thank you very much for coming for the interview and agreeing to participate in this interview. As I had mentioned earlier, we will tape record this interview so that we will be able to capture every opinion, thoughts and ideas when we write a report. Is that still fine with you?

**[PARTICIPANT]:** Yes am okay

**[INTERVIEWER]:** How have you been since the last time we met?

**[PARTICIPANT]:** I am doing fine, I thank God am very much okay.

**[INTERVIEWER]:** How have you been since you knew your HIV status?

**[PARTICIPANT]:** I am doing fine, I have no worries and I am taking my medication as prescribed.

**[INTERVIEWER]:** What motivates you to take the medication?

**[PARTICIPANT]:** The advice I was given by the counsellor.

**[INTERVIEWER]:** What about your own personal motivation?

**[PARTICIPANT]:** to prevent it from spreading.

**[INTERVIEWER]:** Okay. You just said that you are doing good, how did you manage to be doing good since you knew your HIV status?

**[PARTICIPANT]:** I stopped worrying and accepted my HIV results.

**[INTERVIEWER]:** So you mean to say that in the beginning it wasn't easy?

**[PARTICIPANT]:** Yes, it wasn't easy.

**[INTERVIEWER]:** How were you feeling on the day that you took the test and found out that you were infected?

**[PARTICIPANT]:** I was surprised because I was not expecting the result I got.

**[INTERVIEWER]:** What equipment was used for the test?

**[PARTICIPANT]:** RNA

**[INTERVIEWER]:** Okay. What about the other type of HIV test what were the results?

**[PARTICIPANT]:** The results were negative.

**[INTERVIEWER]:** How easy was it for you to accept the RNA result even when you had not seen it?

**[PARTICIPANT]:** It was easy because that's what the RNA result were and I had no otherwise.

**[INTERVIEWER]:** But right now you said that you are fine and taking your medication as well, right?

**[PARTICIPANT]:** Yes, I am

**[INTERVIEWER]:** I am happy to hear that, do you have any fear or worry about this interview?

**[PARTICIPANT]:** No, I have no worry at all.

**[INTERVIEWER]:** I want to ask you about your frequency of getting tested and your knowledge about this.

**[PARTICIPANT]:** Okay.

**[INTERVIEWER]:** When you discovered that you were infected, what made you go for a HIV test?

**[PARTICIPANT]:** I was enrolled in a study and it required me to test regularly that is every month.

**[INTERVIEWER]:** so you went to get tested because you were on the research. Did you have any early signs or symptoms of HIV?

**[PARTICIPANT]:** yes I did.

**[INTERVIEWER]:** what symptoms did you have?

**[PARTICIPANT]:** Feeling sickly, weak, pain in the joints, flu and fever.

**[INTERVIEWER]:** Any other sign?

**[PARTICIPANT]:** No that's all.

**[INTERVIEWER]:** Okay, when you got those signs what came into your mind?

**[PARTICIPANT]:** I thought a lot but didn't get any answer.

**[INTERVIEWER]:** What did you think it was?

**[PARTICIPANT]:** I didn't have any idea because this was the first time I felt that way.

**[INTERVIEWER]:** Haven't you ever been sick?

**[PARTICIPANT]:** I have but this was severe.

**[INTERVIEWER]:** Didn't you suspect that you were infected with HIV?

**[PARTICIPANT]:** Yes I did suspect.

**[INTERVIEWER]:** What was it that made you suspicious of being infected with HIV?

**[PARTICIPANT]:** Because I had been having unprotected sex and I wasn't adhering well to PrEP.

**[INTERVIEWER]:** Okay and right now how are you having sex?

**[PARTICIPANT]:** I am using protection right now.

**[INTERVIEWER]:** What motivates you to use protection right now?

**[PARTICIPANT]:** Because I know I am HIV positive and I wouldn’t like to be responsible to make someone go through what I went through, It is not my wish to infect others that is why am trying hard to use protection.

**[INTERVIEWER]:** What do you think of the ways of PNS and what way did you prefer using?

**[INTERVIEWER]:** There were different strategies but I opted for having health care provider calling them and invite them to come and test.

**[INTERVIEWER]:** Did you use that with all of your partners or there others that you chose to use another method?

**[PARTICIPANT]:** Yes

**[INTERVIEWER]:** What do you think about the methods you chose to use?

**[PARTICIPANT]:** They were nice.

**[INTERVIEWER]:** Were all of your sexual partners tested for HIV?

**[PARTICIPANT]:** Yes.

**[INTERVIEWER]:** How do you know that they were all tested for HIV?

**[PARTICIPANT]:** I was making follow up on each in a discreet manner and they all confirmed to have tested recently at this facility.

**[INTERVIEWER]:** That's okay. I am going to get back to the question I asked you before, seems I wasn't that clear maybe. How was PNS introduced to you?

**[PARTICIPANT]:** After I took the HIV test.

**[INTERVIEWER]:** Okay, explain to me I want to hear more if you are comfortable.

**[PARTICIPANT]:** After I took the HIV test and found out that I was HIV positive, the counsellor asked me to contact my sexual partners for them to be tested too because I also didn't have an idea of who infected me.

**[INTERVIEWER]:** When did you get to know about the PNS, was it on the same day that you got your HIV results or was it done later?

**[PARTICIPANT]:** We briefly discussed it on same day but the actual discussion and detailed of the services were discussed after a day, No it was discussed on the same day I think.

**[INTERVIEWER]:** Then you started talking about PNS on that very day?

**[PARTICIPANT]:** Yes.

**[INTERVIEWER]:** What is your opinion on asking someone who has just found out that he/she is HIV positive about notifying or talking about his/her sexual partners?

**[PARTICIPANT]:** My opinion is that the person who is infected shouldn't be stressed or worried just be yourself.

**[INTERVIEWER]:** I want you to think of all your sexual partners. How many sexual partners did you have?

**[PARTICIPANT]:** Four.

**[INTERVIEWER]:** Okay, I want you to think of all your sexual partners and I'll be asking you about each one of them. Which sexual partner do you prefer I start with, but don't mention any name let's just start with the first one you brought for the HIV testing. How did he react when you brought him to [RESEARCH_INSTITUTION]?

**[PARTICIPANT]:** he was scared.

**[INTERVIEWER]:** Did that affect your relationship in any way?

**[PARTICIPANT]:** No it didn't.

**[INTERVIEWER]:** You still see each other and talk?

**[PARTICIPANT]:** Yes, but we do not have any sexual relations anymore.

**[INTERVIEWER]:** Why is that?

**[PARTICIPANT]:** am the one who stopped.

**[INTERVIEWER]:** With just him or even the others?

**[PARTICIPANT]:** Yes with all of them.

**[INTERVIEWER]:** How was your partner contacted?

**[PARTICIPANT]:** Health care provider from [RESEARCH_INSTITUTION] to contact my partner.

**[INTERVIEWER]:** okay, now on to the next partner, was he contacted and tested for HIV?

**[PARTICIPANT]:** Yes

**[INTERVIEWER]:** What method did you use?

**[PARTICIPANT]:** Health care provider from [RESEARCH_INSTITUTION] to contact my partner. Because that was the safest way for me I didn't want to expose myself to any risk or harm

**[INTERVIEWER]:** what kind of risks or harm do you think might have come your way?

**[PARTICIPANT]:** Violence, rejection and I being exposed to other people.

**[INTERVIEWER]:** What were the results?

**[PARTICIPANT]:** I don't know he did not disclose their status to me

**[INTERVIEWER]:** So it hasn't affected your relationship in any way?

**[PARTICIPANT]:** It hasn't

**[INTERVIEWER]:** Okay on to the third sexual partner, was he contacted and tested for HIV?

**[PARTICIPANT]:** Yes.

**[INTERVIEWER]:** Did it affect your relationship in any way?

**[PARTICIPANT]:** Yes, it did because after he was tested and got his result I have not seen him since then.

**[INTERVIEWER]:** Was this the one you told me that you highly suspect infected you?

**[PARTICIPANT]:** Yes, he's the one.

**[INTERVIEWER]:** Was it a man or a woman?

**[PARTICIPANT]:** A man.

**[INTERVIEWER]:** So it didn't affect your relationship it's just he had he's fears and worries?

**[PARTICIPANT]:** Yes.

**[INTERVIEWER]:** Okay to the last partner, did she take the HIV test?

**[PARTICIPANT]:** Yes she did.

**[INTERVIEWER]:** What method was used?

**[PARTICIPANT]:** The same method but she also went missing after she got her result.

**[INTERVIEWER]:** Who advised her?

**[PARTICIPANT]:** Me, I told her lets go to [RESEARCH_INSTITUTION] and get tested because it's been long since we took a HIV test.

**[INTERVIEWER]:** Was it a man or a woman?

**[PARTICIPANT]:** A man.

**[INTERVIEWER]:** Did you mention this last partner at first or you just recently added him?

**[PARTICIPANT]:** No I initially just mentioned three partners but I came back and added one more.

**[INTERVIEWER]:** What could be the reason for not adding the fourth partner at the beginning?

**[PARTICIPANT]:** I didn't remember the last partner at that time but when I remembered and I was at the clinic I told the counsellor I saw on that day.

**[INTERVIEWER]:** Yes, I disclosed to someone today.

**[PARTICIPANT]:** And who was it?

**[PARTICIPANT]:** My uncle.

**[INTERVIEWER]:** Why did you decided to open up to your uncle?

**[PARTICIPANT]:** It just came out.

**[INTERVIEWER]:** Did you just tell him about your HIV status or even about your sexual practices?

**[PARTICIPANT]:** He just asked me about the ARVs because he saw them and that's when I told him that I went and got tested and found out that I am HIV positive and that's when I got these medicine. I only disclosed about the HIV status.

**[INTERVIEWER]:** What made you chose what to disclose and what not to disclose?

**[PARTICIPANT]:** Some information like who I have sex with is a bit private and if I suspect it might bring issues I would rather not disclose that.

**[INTERVIEWER]:** Did he tell any other family member?

**[PARTICIPANT]:** No, I haven't heard any other family member talking about it.

**[INTERVIEWER]:** So you believe that he hasn't told anyone?

**[PARTICIPANT]:** Yes.

**[INTERVIEWER]:** Do you think you telling your uncle about your HIV status has affected you in anyway?

**[PARTICIPANT]:** It hasn't

**[INTERVIEWER]:** How do you feel that you've told a family member about your HIV status?

**[PARTICIPANT]:** I feel good, I have no worries. He can emotional support me and encourage me as a matter of fact he is doing it already.

**[INTERVIEWER]:** Is there anyone else apart from your uncle that you have also disclose about HIV status?

**[INTERVIEWER]:** I'm going to ask you the Safety in PNS, the partners that have been contacted and tested were there any effects after all that, for example like fighting or suspecting you or ending the relationship with you?

**[PARTICIPANT]:** No, nothing like that happened.

**[INTERVIEWER]:** What's your opinion about a person remembering or knowing their sexual partners in the past 12 months?

**[PARTICIPANT]:** It's not an easy thing to do because of having multiple sexual partners and a year is a long time.

**[INTERVIEWER]:** Why didn't you decide to approach them and tell your partners about your HIV status?

**[PARTICIPANT]:** I was afraid of the other person's reaction, they can start being violent because they were not expecting it.

**[INTERVIEWER]:** What way can this be invented to help the counsellors get in contact with the partners?

**[PARTICIPANT]:** Counselling or a health care provider at [RESEARCH_INSTITUTION] to help out.

**[INTERVIEWER]:** What's your opinion about PNS?

**[PARTICIPANT]:** It is a good thing because if the partner knows his/her HIV status too he/she will be able to get the treatment early enough or just in time.

**[INTERVIEWER]:** What if your partner takes the HIV test and is found negative, how will that benefit him/her?

**[PARTICIPANT]:** He/she will get the chance to prevent himself/ herself from getting infected by using condoms or getting PrEP

**[INTERVIEWER]:** What if your partner takes the HIV test and is found positive, how will that benefit him/her?

**[PARTICIPANT]:** He/she will get counselled and will be given the ARVs.

**[INTERVIEWER]:** What's your opinion about PNS to men who have sex with men?

**[PARTICIPANT]:** It's okay, because they will learn ways of preventing the spread of the virus.

**[INTERVIEWER]:** What's your opinion on the methods of PNS?

**[PARTICIPANT]:** All of them are okay and reliable.

**[INTERVIEWER]:** Why did you choose the strategy you opted for?

**[PARTICIPANT]:** I was worried about the reaction and outburst of my partners. I feel the provider can handle it better.

**[INTERVIEWER]:** How long do you it should take before a person is introduced to PNS? **[PARTICIPANT]:** After a month, so as to give the person time to process everything.

**[INTERVIEWER]:** Don't you think that a month is a very long time? That will be disadvantageous to the partners who might be infected too and don't know yet, why is a month good for you?

**[PARTICIPANT]:** To give time to the client to accept and stop feeling worried, stressed and angry about the result he/she just got.

**[INTERVIEWER]:** If you were the counsellor and I was the client when would you have introduced me to PNS? And why?

**[PARTICIPANT]:** After a week, because after a week someone might have already had time to think it through and accepted their status.

**[INTERVIEWER]:** Any recommendations in the PNS?

**[PARTICIPANT]:** Yes I do, I want to be a living example to the people who will turn out to be HIV positive that you can still live your life even when you are infected as long as you abide by the diet and dosage of the ARVs. I can't motivate them to accept and move forward with their lives.

**[INTERVIEWER]:** do you have any other recommendations a part from being a role model?

**[PARTICIPANT]:** No, I have nothing else to discuss.

**[INTERVIEWER]:** Thank you very much for your contribution, we have come to the end of our interview.

**[INTERVIEWER]:** thank you very much for coming and dedicating your time for sharing your views and experiences, I truly appreciate.
