# Supplementary material for: Peer-assisted HIV partner notification services to strengthen index partner testing for newly diagnosed men who have sex with men in coastal Kenya
Source: PLoS One. 2025 Oct 7;20(10):e0333707. doi: 10.1371/journal.pone.0333707 (PMC12503256; doi:10.1371/journal.pone.0333707)
Supplement: S3 Appendix — (ZIP) [file pone.0333707.s003.zip › Deidentified IDI Transcript_1142.docx]

**Participant characteristics:**

Age: 30 - 34

Sexuality: Bisexual

Education level: Secondary

Days between enrollment and IDI: 30 days

Mobilization strategy: OST

Final PNS Strategy: Index

**[INTERVIEWER]:** Welcome, I will be your interviewer today [DATE] and your study number is 1142 and this interview is conducted at [CLINIC_B].

We would like to understand about notifying your partner/s about your status on HIV after testing. Remember partner notiﬁcation means your partner or the partner of the person infected with HIV/AIDS is notified about their risk for HIV he/she is advised to test for HIV.

**[PARTICIPANT]**: yeah

**[INTERVIEWER]:** A person who is tested positive is called an index client so, you are the index client ok?

**[PARTICIPANT]:** yeah

**[INTERVIEWER]**: So, partner notiﬁcation study is a way of volunteering with your permission in which you are the index client to tell us about your partners, so that they can get tested for HIV and AIDS.

**[PARTICIPANT]:** yeah

**[INTERVIEWER]:** This service is provided by trained Personnel, More so counselors and other Health care providers. In PNS there is a way in which you are assisted so that your partners will be notified about your HIV status. So, when this assisted approach is used, health care provider gives information to your partner of their possibility of contracting HIV.

**[PARTICIPANT]:** yeah

**[INTERVIEWER]:** and when you are being assisted to notify your partners a clinician explains to your partner, concerning their risks of being infected without mentioning your name.

**[PARTICIPANT]:** yeah

**[INTERVIEWER]:** In Kenya, it is now national policy to offer assisted PNS to someone who has tested positive for HIV. Assisted PNS have been studied in heterosexuals in Kenya and are considered safe: there was no intimate partner violence.

**[INTERVIEWER]:** we have little information on how people can be notiﬁed or concerning partner notiﬁcation study on men who have sex with men or transgender and men who have sex with men and women.

**[PARTICIPANT]**: yeah

**[INTERVIEWER]:** on this interview I would like to hear much on your experience on this service on how you notiﬁed you partners and if you think this service will be best for GBT group.

**[INTERVIEWER]:** How are you feeling since your diagnosis? Let's talk about you first.

**[PARTICIPANT]:** I was shocked since I did not know I had HIV but later I took it as something normal since HIV is there and many people have it. I started using ARV's, now I feel like am normal because I use the drugs like others so have just taken it as normal. And since I knew my HIV status, I stated avoiding some people because I have to change my life style. There is nothing I can do, but to focus on taking my pills.

**[INTERVIEWER]:** do you feel comfortable talking about your experience?

**[PARTICIPANT]:** Yes.

**[INTERVIEWER]:** you don't have any problem with that, you are not scared or anything?

**[PARTICIPANT]:** no am ok.

**[INTERVIEWER]:** What made you come for the HIV test?

**[PARTICIPANT]:** What made me come for the HIV test, you know, person has so many partners reaches a point where they feel that they want to know their status. Without this test then you will not know if you are infected or not. The viruses will continue replicating in the body one's knowledge. Then it will reach a point that this person becomes weak. So, testing early is good.

**[INTERVIEWER]:** So, what made you want to know your HIV status?

**[PARTICIPANT]:** What made me want to know my status was because, most people go for HIV test when they look very healthy. Sometimes we rely on our partners HIV results. I may say to myself that I have so many partners thus I need to know my status. If one tests negative they say thanks and they become happy, if they test positive, then they get shocked but after counseling and starting the ARV's then one becomes better.

**[INTERVIEWER]:** Can you remember when you got tested for HIV, when was it?

**[PARTICIPANT]:** Month of April I think [DATE] or [DATE]

**[INTERVIEWER]:** April or May, last month or the previous month.

**[PARTICIPANT]:** Last month

**[INTERVIEWER]:** Last month the 5th month.

**[PARTICIPANT]:** Yeah.

**[INTERVIEWER]:** which date?

**[PARTICIPANT]:** I cannot clearly remember the date but it was between [DATE] to [DATE],

**[INTERVIEWER]:** did you go for the test or did you expose yourself and you thought of going for HIV test or did you have sex with a new partner?

**[PARTICIPANT]:** I was not doubting myself in any way, but I decided to accept myself after receiving advice from health worker I met.

**[INTERVIEWER]:** Peer educator?

**[PARTICIPANT]:** yeah, peer educator who came and insisted that it's good to know my HIV status if I test positive or negative I'll be guided accordingly and I thought it is good because it involved my health. When I tested, it turned positive.

**[INTERVIEWER]:** Let's talk about the day you were mobilized by the peer educator and he told you about research, what you were happy about. What did he tell you for you to make a decision to join the research?

**[PARTICIPANT]:** He called a meeting with some people. He did not approach me.

**[INTERVIEWER]:** A group of people?

**[PARTICIPANT]:** Yeah, a group of 2 to 3 people. On listening since I was close to the group I thought no this concerns my health, I joined them. when I listened, the information was good and I decided to join in and do a HIV test which turned positive, and we agreed to come to [RESEARCH_INSTITUTION] clinic for further tests to confirm this status.

**[INTERVIEWER]:** please let's go back a little bit and try to explain to me what the peer mobilizer was saying what attracted you in joining the group? what was he saying exactly? what information was he giving?

**[PARTICIPANT]:** He told us at [RESEARCH_INSTITUTION] HIV testing is free and when you test positive you are taken care of without incurring any expenses, including transport and your health is well taken care of. And this gave me encouragement to know my status.

**[INTERVIEWER]:** What did you mostly talk about, your risks or oral self-test or the possibility of you getting HIV?

**[PARTICIPANT]:** He had a kit which he said it was for self-test. The peer educator explained to us on how to use it. He gave them to those were ready to do a HIV self-test. There are some who used it and there are some who did not use it.

**[INTERVIEWER]:** What did you do?

**[PARTICIPANT]:** I used the kit and because the result was positive I had to come to [RESEARCH_INSTITUTION] for confirmatory test.

**[INTERVIEWER]:** Did the peer mobilizer tell you about early symptoms of HIV called acute HIV infection?

**[PARTICIPANT]:** He explained and told us that we men who have sex with men and women are at high risk of getting HIV because we are engaged in unprotected sex without caring. That's the reason as to why I eagerly followed what the peer mobilizer was explaining.

**[INTERVIEWER]:** What do you think you understood from that conversation?

**[PARTICIPANT]:** What I understood from the conversation is that, it mostly touched on one's health as an adult. I could not ignore such information. That's when I decided to come to [RESEARCH_INSTITUTION] for the services, and when I came, I was served well and I confirmed my results to be truly HIV positive. He really helped me know my status.

**[INTERVIEWER]:** Let's talk about the topics that the peer educator was discussing, were you aware of the information he was giving?

**[PARTICIPANT]:** No.

**[INTERVIEWER]:** You had no idea of what he was talking about? You have never heard of received this information elsewhere?

**[PARTICIPANT]:** no, I only heard when I met him.

**[INTERVIEWER]:** Did he give you anything maybe papers containing some explanation or did he give you a book or anything that you can read and understand better?

**[PARTICIPANT]:** No, he told me that if I want to know more I should come to [RESEARCH_INSTITUTION], he gave us advise, explained to us, we did not ignore him we trusted him because what he was saying was something understandable. He also said that if we were ready we could accompany him to [RESEARCH_INSTITUTION]. We told him that we could manage to go to [RESEARCH_INSTITUTION] the day that following day.

**[INTERVIEWER]:** How was your experience of being mobilized by the peer educator?

**[PARTICIPANT]:** It was good I listened to him and understood him because the results were fruitful since am now using the drugs and am under treatment. My experience was I should not ignore anyone who is giving out vital information.

**[INTERVIEWER]:** How do you think we could motivate more GBT people at-risk of HIV to take an HIV test?

**[PARTICIPANT]:** They should get an advisor a good one to advise them, they will be able to make better decisions and those who ignore will later on regret because the advice will be about their own health.

**[INTERVIEWER]:** How did you get your test results, and what was the experience like?

**[PARTICIPANT]:** you know the peer educator had the kits and I had the temptation to pick one and use in order to ﬁnd the truth about my HIV status. So, I took and tested myself though I did not know how to use the Kit. When the results came out I didn't believe the results. That's when I called him and asked if there was another test apart from using the OST kit, he said yes, and that we should accompany him the next morning to [RESEARCH_INSTITUTION] clinic for confirmatory. When I arrived at [RESEARCH_INSTITUTION], I was tested and the results came out HIV positive.

**[INTERVIEWER]: Ok**, did you get the ART drugs the same day.

**[PARTICIPANT]:** Yes, I started the medication the same day.

**[INTERVIEWER]:** Tell me how was the experience? what was done to you before you were given the drugs.

**[PARTICIPANT]:** I was tested, the results turned positive and I was advised on how I will be using the long-term therapy.

**[PARTICIPANT]:** Ooh, am sorry am forgetting, I never started the medication that same day, the health worker advised me on how to use the drugs. He also wanted to know my partners, either their phone numbers or how we can contact them so that they come and know their HIV status. I gave out their phone numbers and that they will be contacted. I was then given an OST kit to take to my regular partner. I requested to be given time to think about the long-term medication. So, the next day I came back and I was initiated to the medication.

**[INTERVIEWER]:** How did you feel starting these medications knowing that you were going to use it for the rest of your life?

**[PARTICIPANT]:** I ﬁrst asked him that question, for how long will I be taking these pills? And he said that I was going to use them daily until the viral load suppressed completely probably until zero and I will also continue using them for the rest of my life. 1 asked how long was it going to take and he said for life, he even said their people who have used for more than 20 years. That's when I realized I will be depending on drugs for survival if not the virus would kill me. This reality stressed me especially the ﬁrst week. I became angry but later I decided just to accept myself since I was not used to the life of using drugs. Stressed me was the endless use of the drugs all through my life which only God knows. I now take it as normal and am no longer stressed.

**[INTERVIEWER]:** Tell me about the counseling you received.

**[PARTICIPANT]:** When I received the results I didn't believe, I did not trust the OST Kit completely since I knew that, if you one is to be tested blood must be drawn ﬁrst. That's why I ignored the OST kits results and asked the peer educator to take me with the others to [RESEARCH_INSTITUTION] Clinic and he agreed.

**[INTERVIEWER]:** What happened?

**[PARTICIPANT]:** he asked me to interpret the results, I said there were two lines and he said that if they were two, then I was positive but I shouldn't worry. I said to him that I would like test to be done using my blood because I felt the test kit has is faulty.

**[INTERVIEWER]:** How did you feel after being told not to worry?

**[PARTICIPANT]:** I calmed down and waited to visit the clinic for confirmatory test. When I tested positive, I lost my mind but the counselor did not realize.

**[INTERVIEWER]:** what happened?

**[PARTICIPANT]:** I started behaving weirdly. My thoughts were far and I started talking to myself that I really had the virus. I witnessed some of my friends suffering and also dying due to AIDS. If someone tests positive counselors should have a unique formula to explain the situation because at that time the person is not in their right mind, they may do something dangerous.

**[INTERVIEWER]**: Something dangerous like?

**[PARTICIPANT]**: Like carrying the anger grudge to their, which is very dangerous. He may leave the counseling room and the clinic place nicely but due to the thoughts that they are going to die due to sex they might even think of killing all their partners/lovers since they may feel they are no longer living but they will die of the virus anyway.

**[INTERVIEWER]:** That's how you felt after the results?

**[PARTICIPANT]:** Yes, but ﬁrst I am grateful because I didn't start the medication that same day. I went home, slept and come the following day alone without the peer educator. I felt that I had accepted that situation.

**[INTERVIEWER]**: I would like to talk to you about PNS, how was it introduced to you?

**[PARTICIPANT]:** I was told that the research aims to help those who are infected to know their status so as to save their life. So, this research help people to know their status and to start medication so that they live a healthy life. If it was not for this research I don't know where I would be.

**[INTERVIEWER]**: How do you feel about the counselling you received on notifying your sex partner(s)?

**[PARTICIPANT]:** we discussed about different strategies of notifying my partners. One was if possible I should use the counselor by giving him my partners phone numbers he can contact them without me being involved. There are those partners that I can use the counselor or health provider and others who I can only contact them without any assistance due to how they will react.

**[INTERVIEWER]:** These are the methods/strategies you were advised to use?

**[PARTICIPANT]:** Yes

**[INTERVIEWER]:** Which strategy did you choose?

**[PARTICIPANT]**: I decided that there are those that I may advise without any assistance and those that I will give their numbers to the counselor, who will later call them. There is also one of my friends that was called and informed me that he was contacted by a health provider at [RESEARCH_INSTITUTION] to visit the clinic, my duty was to make sure that he truly obeys the call and visits the center.

**[INTERVIEWER]**: Apart from that, how did you feel about the strategies that you selected?

**[PARTICIPANT]**: They were okay.

**[INTERVIEWER]:** Am grateful for what you have told me, and now let's talk about your partners whom you decided to notify. How many were they?

**[PARTICIPANT]:** Three.

**[INTERVIEWER]**: How was your first partner notified for HIV?

**[PARTICIPANT]:** He was contacted and notified by the [RESEARCH_INSTITUTION] health worker, he has not come, but we should expect him any day.

**[INTERVIEWER]:** This is a person that you gave out his number to the counselor? Oh, he now wants to visit the clinic?

**[PARTICIPANT]:** Yes

**[INTERVIEWER]:** And tell me about the one you took for the OST kit, kindly explain how it was, how was the experience?

**[PARTICIPANT]:** When I went with the kit, I waited for three days for him to arrive from [CITY_D] since he wasn't around. He came and as we were discussing about our normal issues and because he is a person that I know him very well and I understand, I told him that there are ways of testing your health without even going to the doctor would you like to test? he accepted and I started showing him how to use the Self-test kit as shown by the peer educator. Few minutes later the results were out and he was positive, but you know that the kit is not trusted by many.

**[INTERVIEWER]:** Did he believe the results?

**[PARTICIPANT]**: He was ﬁfty ﬁfty, he could be here today but due to his nature of work he may not be able to come soon. He is ready to come for the blood tests so that he can believe the test results or accept the outcome of the confirmatory test. I will also come with him but, I will pretend that I've never been here before, I will pretend that it is my ﬁrst time here.

**[INTERVIEWER]:** Has PNS affected you?

**[PARTICIPANT]**: No.

**[INTERVIEWER]:** And do you see whether it has affected you and your partners?

**[PARTICIPANT]:** The effects that I've seen is just stress from the positive results, which makes people not so confident with self-test results. I also see that some people should not use the kit because if the results are positive and the person is short tempered, he or she may do something that is not good.

**[INTERVIEWER]:** Did your regular partner change when you were with him when he was using the self-test kit.

**[PARTICIPANT]:** Yes, he did, but what made him not react was the fact that he never believed the kit. So, this kit when taken to someone and results read positive, and they realize that they are truly positive and are not near a health provider then they may cause some problems or become violent.

**[INTERVIEWER]:** Ok, and did the results affect your relationship?

**[PARTICIPANT]:** No

**[INTERVIEWER]:** Apart from this regular partner are there other partners you haven't notiﬁed?

**[PARTICIPANT]:** Yes, but those that I haven't mentioned are those from far who can't be easily reached. I have mentioned around three people.

**[INTERVIEWER]:** Yes, because here I see a list of around three people.

**[PARTICIPANT]:** Yes,

**[INTERVIEWER]:** these other partners, how far are they?

**[PARTICIPANT]:** like [CITY_E]

**[INTERVIEWER]:** And, are there any other ways that those from far can be reached? either by text message since they also have a right to know about their status? will it be okay if they are notified?

**[PARTICIPANT]:** It will be okay if they are notiﬁed because if not it's like I will be letting them become sick yet they are not aware.

**[INTERVIEWER]:** Will you mind giving us their phone numbers or which method do you prefer to use?

**[PARTICIPANT]:** There are those that I may give out their numbers and those that I may notify.

**[INTERVIEWER]:** lets speak talk about disclosure, have you disclosed your status to anyone else?

**[PARTICIPANT]:** No

**[INTERVIEWER]:** Whom do you want to tell?

**[PARTICIPANT]:** The one I would like to disclose to ﬁrst is my wife, because I am gay for business only. I will explain to her face to face in front of a health provider.

**[INTERVIEWER]:** Why do you want your wife to know?

**[PARTICIPANT]:** Because this is a virus and am infected, the only person who will understand me is the person who knows me and very close to me. A friend my not accept me and I may be a burden to them but for a close person like her she will be with me till the end.

**[INTERVIEWER]:** how will disclosing to your wife affect you?

**[PARTICIPANT]:** That's why I prefer going with her to the health center for her to be tested ﬁrst?

**[INTERVIEWER]:** How will this affect you?

**[PARTICIPANT]:** I just need someone close to me to know mostly my wife and that's why we will go to for a test together. First the test will be done to her, if she's positive, I will be open and disclose to her. But if she's negative, then I will use a health worker to help me disclose.

**[INTERVIEWER]:** You say that's when you will need the doctor's assistance, what do you think can happen if she's negative?

**[PARTICIPANT]:** You know, a HIV positive and negative person don't rhyme, especially if they are married. She may refuse to listen to me or she may even want a divorce. That's why I will need the doctor's assistance to help. So that our relationship last.

**[INTERVIEWER]:** Let's now talk about your security. Did you experience any safety issues or other harms as a result of notifying your partner(s)?

**[PARTICIPANT]**: You know the fear of harm made separate those that am going to use the health worker to notify and those I will notify myself.

**[INTERVIEWER]:** what kind of insecurity do you think may arise? It could be ﬁght, losing your house or even being rejected by family members or ending of relationship.

**[PARTICIPANT]**: Apart from being rejected by family members, I may lose a client, an angry newly infected person may decide to spread the virus to other innocent people. If my client ﬁnds out that they are HIV positive, he may deﬁnitely think that I am the cause.

**[INTERVIEWER]:** According to you were there any, think of the partner you took the self-test to.

**[PARTICIPANT]:** No, but the problem may arise from those who I gave out their phone numbers to the counselor.

**[INTERVIEWER]:** Kenyan national policy recommends notification of all sex partners in the last 12 months before someone tests positive for HIV. What was it like to discuss your sex partner(s) with me during our first interview?

**[PARTICIPANT]**: The ﬁrst day?

**[INTERVIEWER]:** Yes, how was it?

**[PARTICIPANT]**: If I had the ability to inform them myself, i could have done it. There are those who could be notified and those who I couldn't.

**[INTERVIEWER]:** My question is, how did you feel talking of your partners?

**[PARTICIPANT]:** I felt well, I had the freedom and confidence to do so.

**[INTERVIEWER]:** But you said that you only named a few and gave out their number, what made you not to name all of them?

**[INTERVIEWER]:** Okay. Is it possible for you to give me the number of people you've been involved with sexually within the last twelve months?

**[PARTICIPANT]:** They are more than ten.

**[INTERVIEWER]:** Are you be able to give out their phone numbers?

**[PARTICIPANT]:** There those that their numbers are available and others are not.

**[INTERVIEWER]:** Okay. And if they are more than ten, how may are they? You know more than ten can even be 10000 so how may are they exactly?

**[PARTICIPANT]:**13 to 15 people

**[INTERVIEWER]:** Are they 13 or15

**[PARTICIPANT]:**13

**[INTERVIEWER]:** Could you tell me of some ways that helped (or could help) you disclose / invite your partner(s) for testing?

**[PARTICIPANT]:** The best way, is to advise them indirectly. When communicating I should discuss about health. That is how I would have advised them to know their health status. To my partners will advise them to use my way of notifying them or use the health worker to contact their partners. Assuming that I am talking to a group I will just tell them that it is better for us to go for a health checkup frequently.

The Self-test kit would be ideal, but my fears are that most of the people may have a problem if they test positive. They may not believe the results.

**[INTERVIEWER]:** Your best strategy is to use the self-test kits?

**[PARTICIPANT]:** Yes, a Self-test kit and also advise from the health care providers.

**[INTERVIEWER]:** The WHO recommends PNS and recently the Kenyan MOH adopted the approach, which is now recommended for clients who test HIV positive. Tell me about the challenges that may arise from this PNS strategies or barriers

**[PARTICIPANT]:** There are no challenge but one issue is that it may not be easy for someone who just tested positive to accept their results. Unless they receive proper counseling and advice from the health workers.

**[INTERVIEWER]:** How do you feel about PNS?

**[PARTICIPANT]:** it is a good service since it helps people to know about their health.

**[INTERVIEWER]:** Would you recommend this service to others.

**[PARTICIPANT]:** Yes.

**[INTERVIEWER]:** Why?

**[PARTICIPANT]**: Because it saves lives.

**[INTERVIEWER]:** If PNS were to be provided to GBT who test HIV positive, what are your thoughts?

**[PARTICIPANT]:** It would be good to inform partners if they are at risk because It wouldn't be good to spread the virus even on the eyes of the Lord.

**[INTERVIEWER]:** Do you foresee any positive outcomes or benefits?

**[PARTICIPANT]:** The beneﬁt is, it may help them be aware of their risks.

**[INTERVIEWER]:** As we discussed before, there are different methods to notify sex partners of a person who is diagnosed with HIV. [Show PNS flyer and discuss methods]. What do you think about these different methods for PNS?

**[PARTICIPANT]:** they are all good but I would prefer using the peer educator or a counselor.

**[INTERVIEWER]:** Why?

**[PARTICIPANT]:** They advise people.

**[INTERVIEWER]:** Apart from these strategies, could you think of another strategy?

**[PARTICIPANT]:** I don't think so.

**[INTERVIEWER]:** How much time after your HIV diagnosis was PNS discussed with you?

**[PARTICIPANT]:** I was told on the same day.

**[INTERVIEWER]:** What would you envision a good moment to discuss PNS after someone is diagnosed with HIV?

**[PARTICIPANT]:** The same same day.

**[INTERVIEWER]:** Why?

**[PARTICIPANT]:** Because if it is not on the same day then a person may not know the benefit of counseling and also notifying their partner.

**[INTERVIEWER]:** Could you come up with communication strategies for HCP to discuss PNS with a person who tested positive for HIV?

**[PARTICIPANT]**: One should not to give up, since life is not yet over and, take medication and eat healthy.

**[INTERVIEWER]:** I mean the PNS services, the precise words to introduce the topic.

**[PARTICIPANT]:** This service helps you to help you accept and cope with the use of long term medication anywhere anytime. If you have partners please tell us so that we know we can help you notify them. If they are infected, then they can benefit from ART's and if they are not infected then they can benefit from taking PrEP.

**[INTERVIEWER]:** Just the introduction only. A short introduction topic on PNS.

**[PARTICIPANT]:** Our service, helps you to notify your partners and also you are getting your drugs on time. We will make sure that you are well taken care of.

**[INTERVIEWER]:** Thank you for that. Would you have any recommendations for implementing PNS for GBT in Kenya?

**[PARTICIPANT]:** Thank you for helping me to know my status and starting to take ART, if it was not for this service, I would have been greatly affected I am taking my drugs well and I am better now.

**[INTERVIEWER]:** Thank you for accepting to be recorded and thank you for coming to our clinic today.

**[PARTICIPANT]:** Thank you too.
