# Supplementary material for: Peer-assisted HIV partner notification services to strengthen index partner testing for newly diagnosed men who have sex with men in coastal Kenya
Source: PLoS One. 2025 Oct 7;20(10):e0333707. doi: 10.1371/journal.pone.0333707 (PMC12503256; doi:10.1371/journal.pone.0333707)
Supplement: S3 Appendix — (ZIP) [file pone.0333707.s003.zip › Deidentified IDI Transcript_1248.docx]

**Participant characteristics:**

Age: 20-24

Sexuality: Gay

Education level: Vocation

Days between enrollment and IDI: 74 days

Mobilization strategy: OST

Final PNS Strategy: Index

**Partners identified: 4**

**[INTERVIEWER]:** Thank you so much for accepting to attend this interview...so as I stated earlier that I will be recording this for the purpose of scripting this later on. So welcome and feel free. Today is on [DATE]. How are you feeling and how are you feeling?

**[PARTICIPANT]:** I'm okay, feeling good just continuing with my dose.

**[INTERVIEWER]:** When you found out that you were HIV positive for the first time what did it mean to you and how did you take it?

**[PARTICIPANT]:** It was very tough for me at first but I just had to accept it and just start taking the ARVs.

**[INTERVIEWER]:** What made it seem tough?

**[PARTICIPANT]:** I didn't expect the result to turn out the way they did, it was news I just didn't expect to be in that state.

**[INTERVIEWER]:** You said that the first time was hard but with time you turned out to be okay. What facilitated the change from hard to being okay?

**[PARTICIPANT]:** Talking to the counsellors, they talked to me and made me feel that everything is okay.

**[INTERVIEWER]:** What was the reason that made you take the HIV test?

**[PARTICIPANT]:** The reason why I came to get tested is that, I was not feeling well and there were some changes happening, which to me were not normal that's why I went to get tested for HIV.

**[INTERVIEWER]:** Are you comfortable talking about the changes, mentioning the specific changes. What were the changes?

**[PARTICIPANT]:** Um... being sickly, I was just not myself, there some diseases that I found to be petty that I was always suffering from that's why I got tested.

**[INTERVIEWER]:** Okay, so it was like you were suspecting it?

**[PARTICIPANT]:** Yeah I was.

**[INTERVIEWER]:** Okay, but earlier on you told me you didn't expect to be HIV positive and now you are telling me that you were suspecting that's why you came for the test.

**[INTERVIEWER]:** Because I had sex with different people and it was bareback which was wrong.

**[INTERVIEWER]:** Before you took the last HIV test, were you fond of taking the test after every three months or was it your first experience?

**[PARTICIPANT]:** I had taken the test before.

**[INTERVIEWER]:** How long was it before the recent one?

**[PARTICIPANT]:** It was like... three to months back.

**[INTERVIEWER]:** so you mean it was a recent infection...

**[PARTICIPANT]:** Yeah, it was.

**[INTERVIEWER]:** But right now you are okay. How are faring on with the dose?

**[PARTICIPANT]:** I'm doing well.

**[INTERVIEWER]:** And what motivates you to keep on taking the ARVs?

**[PARTICIPANT]:** Moving forward that's what motivates me.

**[INTERVIEWER]:** When you say moving forward what do you mean?

**[PARTICIPANT]:** I'm focusing on having better health I don't want my health to be worse than it is.

**[INTERVIEWER]:** Okay, you don't want it to get worse than how it is, how did you come to [RESEARCH_INSTITUTION], for the first time?

**[PARTICIPANT]:** I came to [RESEARCH_INSTITUTION] through the mobilizer

**[INTERVIEWER]:** How did the mobilizer approach you?

**[PARTICIPANT]:** He called me and told me that they are doing HIV test and other STI are free to test, so I just decided to come knowing that I was also not feeling well for a very long time I just wanted to know.

**[INTERVIEWER]:** Did the mobilizer talk to you about risky behaviours?

**[PARTICIPANT]:** Yes I did.

**[INTERVIEWER]:** Okay can you tell me some of the risky behaviours you discussed?

**[PARTICIPANT]:** Unprotected sex, using of sharing sharp objects with infected people I guess that's all I remember

**[INTERVIEWER]:** What did he tell you about the oral self-test kit?

**[PARTICIPANT]:** He told me how to use it, it was simple and easy and within some few minutes I'll get my results.

**[INTERVIEWER]:** Did he talk to you about acute HIV infection?

**[PARTICIPANT]:** No he didn't.

**[INTERVIEWER]:** What do you know about Acute HIV infection?

**[PARTICIPANT]:** Mmh..I don't know anything

**[INTERVIEWER]:** The mobilizer discussed with you about risky behaviours and OST, how easy or hard was it for you to understand?

**[PARTICIPANT]:** It was very easy to understand, the mobilizer was very clear.

**[INTERVIEWER]:** To what extent did you know about this topic before?

**[PARTICIPANT]:** I used to know them from school and through guidance counselling programs.

**[INTERVIEWER]:** Okay so you had an idea you heard them from the guidance and counselling programs, did the mobilizer share information leaflets like materials papers?

**[PARTICIPANT]:** No he didn't.

**[INTERVIEWER]:** Okay, how can you describe your experience with mobilizer?

**[PARTICIPANT]:** The experience was good.

**[INTERVIEWER]:** It was nice, okay... I need you to tell me more what made the experience good?

**[PARTICIPANT]:** When he told me about this place and how well the services are in a [RESEARCH_INSTITUTION] , so it was good for me. I was happy that everything he told me was exactly what I came and found here in [RESEARCH_INSTITUTION].

**[INTERVIEWER]:** Are there any things in your opinion that you think the mobiliser didn't do other or could have done better?

**[PARTICIPANT]:** There's nothing

**[INTERVIEWER]:** Don't you think it was important for the mobilizer to tell you more about the acute HIV infection?

**[PARTICIPANT]:** I don't, because even if he missed still got information from the guidance and counselling place.

**[INTERVIEWER]:** In your opinion how do you think we can motivate GBT community to be testing for HIV frequently?

**[PARTICIPANT]:** They are those who are still new in the GBT community and those who already have been there for a long time, we should focus on the new ones and get them tested using different platforms such as visiting them in schools, social media, using a friend to tell a friend and existing CBOs to do targeted outreaches.

**[INTERVIEWER]:** How can you describe your experience of using the OST kit? Was it easy or hard?

**[PARTICIPANT]:** It was easy.

**[INTERVIEWER]:** Okay, what was easy?

**[PARTICIPANT]:** Because it was fast I got the results immediately.

**[INTERVIEWER]:** What can you say about the process?

**[PARTICIPANT]:** The process was not hard at all, and it was really good especially for people who are afraid of needles and blood.

**[INTERVIEWER]:** Did you start your treatment the same day you found out that you were HIV positive?

**[PARTICIPANT]:** No, I didn't start on that same day.

**[INTERVIEWER]:** How long did it take you to start the treatment?

**[PARTICIPANT]:** Around 3 days.

**[INTERVIEWER]:** What do you think were the reasons for the delay?

**[PARTICIPANT]:** The reason for the delay was first, I was shocked and did not believe that I was infected with HIV after guide and counselling I got to understand and was ready to start the treatment.

**[INTERVIEWER]:** Okay, so you were low, emotionally disturbed, how did you feel about starting treatment?

**[PARTICIPANT]:** I felt okay because I had to, it was the only way to protect myself from the worse.

**[INTERVIEWER]:** Did you ask yourself other questions like "ill be taking these ARVs for the rest of my life will I be able to do all that?"

**[PARTICIPANT]:** Yes, such things have to be there.

**[INTERVIEWER]:** Then tell me more.

**[PARTICIPANT]:** I felt all that but since I was focused on moving on and being strong and knowing that there's some future ahead. You still have to live, life has to still go on.

**[INTERVIEWER]:** Okay, what can you tell me about the counselling you received?

**[PARTICIPANT]:** The counselling was good.

**[INTERVIEWER]:** Was good right, meaning?

**[PARTICIPANT]:** I understood it very well and followed exactly what I was told.

**[INTERVIEWER]:** Okay, now we are going to talk about sexual partners and your experience with PNS.

**[PARTICIPANT]:** Yeah.

**[INTERVIEWER]:** How was PNS introduced to you? PNS is Partner Notification Services.

**[PARTICIPANT]:** Oh, I was first told by the counsellor, I was told that if my partner knows his status and such and how to get my partner to take the test too.

**[INTERVIEWER]:** What PNS strategies were you told?

**[PARTICIPANT]:** He told me that we can contact my partners through the healthcare service provider...

**[INTERVIEWER]:** yes, the second strategy?

**[PARTICIPANT]:** The second one was for me to go with the OST kit and discuss with my partner and get him to test.

**[INTERVIEWER]:** Any other strategy you remember?

**[PARTICIPANT]:** I only remember those two.

**[INTERVIEWER]:** Okay, what strategy did you choose?

**[PARTICIPANT]:** I chose the second one I mentioned about being given the OST kit.

**[INTERVIEWER]:** Okay, did you manage to issue OST kit to them?

**[PARTICIPANT]:** He was not available...

**[INTERVIEWER]:** Meaning?

**[PARTICIPANT]:** He's in [CITY_D].

**[INTERVIEWER]:** And on phone?

**[PARTICIPANT]:** I prefer discussing these things personally than on phone.

**[INTERVIEWER]:** Okay, what made you decide that strategy?

**[PARTICIPANT]:** I felt comfortable with this strategy, than me giving out his number I wasn't comfortable with that.

**[INTERVIEWER]:** What were your worries about the other strategy?

**[PARTICIPANT]:** I don't know, I just liked the second one.

**[INTERVIEWER]:** Didn't you have any fears like, they might mention my name to him?

**[PARTICIPANT]:** No, I was told that that cannot happen.

**[INTERVIEWER]:** In short you just liked the second one.

**[PARTICIPANT]:** Yes.

**[INTERVIEWER]:** How do you feel about the strategy you chose now?

**[PARTICIPANT]:** I still think it was easier...

**[INTERVIEWER]:** Did you manage to get him to take the test?

**[PARTICIPANT]:** No I didn't I told you that he travelled to [CITY_D] but he'll be back soon.

**[INTERVIEWER]:** When did he travel?

**[PARTICIPANT]:** Its two months now.

**[INTERVIEWER]:** When did you find out that you were HIV positive?

**[PARTICIPANT]:** Can I really remember...I guess three months before.

**[INTERVIEWER]:** So according to all that you had the kit for a month and you didn't manage to make him take the test, what was the reason for the delay?

**[PARTICIPANT]:** He was unavailable and I needed to seriously have a talk with him before I tell him to take the test.

**[INTERVIEWER]:** Okay, so you took time because he's unavailable meaning he didn't have time or he wasn't around completely?

**[PARTICIPANT]:** He wasn't around completely he's under chemotherapy in [CITY_D] so currently he is there. I took the kit the other time because he told me he's going to be here on weekend so I could give it to him on that weekend.

**[INTERVIEWER]:** Now its clear, thank you. So in short he has not been contacted and tested yet.

**[PARTICIPANT]:** Yes.

**[INTERVIEWER]:** Okay, how many partners did you mention during the first interview?

**[PARTICIPANT]:** two partners.

**[INTERVIEWER]:** Now, ill ask you about each, you'll know who we are talking about.

**[PARTICIPANT]:** Yeah.

**[INTERVIEWER]:** Did you choose the same strategy on the other partner?

**[PARTICIPANT]:** I tried calling him and telling him about everything and I got blocked.

**[INTERVIEWER]:** Was it an option you were given to choose?

**[PARTICIPANT]:** Yes, I really preferred talking to them about these things myself, yeah.

**[INTERVIEWER]:** Okay, so this partner was not contacted?

**[PARTICIPANT]:** Yes not yet.

**[INTERVIEWER]:** How do you think telling them all that has affected your relationship with your partners?

**[PARTICIPANT]:** Some people don't like the issue of taking HIV test, I guess when I told him about this he didn't like and decided to block me.

**[INTERVIEWER]:** Do you think they will accept to take the test?

**[PARTICIPANT]:** I don't know about the one who blocked but the one in [CITY_D] will take the test.

**[INTERVIEWER]:** Do you think they will be willing to start using ARVs assuming they are found positive?

**[PARTICIPANT]:** Okay, yeah I do. Because no one wants to have deteriorating health.

**[INTERVIEWER]:** In case they get tested and turn out to be negative do you think they'll be willing to start PrEP?

**[PARTICIPANT]:** I think they might be up for other ways of protecting themselves, because PrEP is associated with stigma out here.

**[INTERVIEWER]:** tell me about examples of stigma associated with PrEP?

**[PARTICIPANT]:** being called a sex worker or a reckless person as far as safe sex is concerned.

**[INTERVIEWER]:** How do you think we can get the partner that blocked you?

**[PARTICIPANT]:** He's actually not from [CITY_G] he's also from [CITY_D], maybe I give you his contacts and you talk him.

**[INTERVIEWER]:** It's very dangerous for them to be out there without knowing their status, they might be spreading the disease unknowingly, so it's important to find ways to get to them. Is there any other partner you forgot to mention at first?

**[PARTICIPANT]:** No, they are just those two.

**[INTERVIEWER]:** Have you disclosed to anyone about your HIV status?

**[PARTICIPANT]:** Yes my friend.

**[INTERVIEWER]:** What kind of a friend, a gay friend, female friend or a straight friend?

**[PARTICIPANT]:** A female friend.

**[INTERVIEWER]:** How much did you tell her, like your sexual orientation?

**[PARTICIPANT]:** Actually she knows my sexual orientation.

**[INTERVIEWER]:** Okay, how did you disclosed?

**[PARTICIPANT]:** I just told her that I was found positive all she did is to encourage me to start my treatment.

**[INTERVIEWER]:** Why did you decide to tell her and not anyone else?

**[PARTICIPANT]:** I trust her and we've been friends for a long time now.

**[INTERVIEWER]:** Has disclosing affect your relationship with her?

**[PARTICIPANT]:** The relationship is just normal.

**[INTERVIEWER]:** How did it affect you when you opened up to her?

**[PARTICIPANT]:** I felt better because I was holding it in and it was hard.

**[INTERVIEWER]:** Have you had any PNS safety issues as a result of you notifying your partners?

**[PARTICIPANT]:** No, I didn't.

**[INTERVIEWER]:** How was it to discuss about your sex partners?

**[PARTICIPANT]:** It was tough because I was not really comfortable yet with the counsellor I was talking to, but as time went by I got comfortable and opened up.

**[INTERVIEWER]:** She asked you about your sex partner in the last 12 months?

**[PARTICIPANT]:** Yes.

**[INTERVIEWER]:** Was it easy for you to remember?

**[PARTICIPANT]:** I had to do a lot of counting (*chuckling*).

**[INTERVIEWER]:** How many did you end up having?

**[PARTICIPANT]:** so many.

**[INTERVIEWER]:** Then how comes you mentioned only two partners?

**[PARTICIPANT]:** Oh no she also asked me from when I started having sex, how many guys have I had sex with that when I started the counting.

**[INTERVIEWER]:** Oh okay and what about in the past 12 months after taking the test?

**[PARTICIPANT]:** They were ten.

**[INTERVIEWER]:** Ten. Okay how comes you mentioned only two?

**[PARTICIPANT]:** The two are the ones I was in a relationship with, the rest were one night stands.

**[INTERVIEWER]:** Don't you think it was important to notify them?

**[PARTICIPANT]:** I didn't find it to be important because I was okay during that time.

**[INTERVIEWER]:** Do you think somebody else might find it challenging to tell the number of partners in the last 12 months?

**[PARTICIPANT]:** (*chuckling*).yes (*chuckling*) there are people who sleep around with a lot of people.

**[INTERVIEWER]:** What will make it challenging?

**[PARTICIPANT]:** For example the sex workers, the cant keep a record of every person they have sex with.

**[INTERVIEWER]:** That's true, can you share with me any idea you think can help reach the partners?

**[PARTICIPANT]:** I think those strategies of PNS are good.

**[INTERVIEWER]:** They are good but you had the OST kit and you've not yet managed to get him to test. What do you think, is one strategy enough or two combined can work better?

**[PARTICIPANT]:** A client might come up with a better idea of reaching his partner, away from the ones in the PNS.

**[INTERVIEWER]:** you are trying to say that we should give room to client to share their ideas.

**[PARTICIPANT]:** yes

**[INTERVIEWER]:** What is your opinion about the PNS?

**[PARTICIPANT]:** PNS is worth it because people have to know their status and through PNS we can get a lot of people to have the HIV test.

**[INTERVIEWER]:** would you recommend PNS to friend who just found out he's HIV positive?

**[PARTICIPANT]:** Yes I definitely would.

**[INTERVIEWER]:** what will make you recommend the services to him?

**[PARTICIPANT]:** Because it will help in reducing the spread of the infection.

**[INTERVIEWER]:** What is your feeling about PNS especially considering the GBT community, do you think if it's something that can really work in the community or not?

**[PARTICIPANT]:** It might work for some people and other not really.

**[INTERVIEWER]:** Tell me more about both sides

**[PARTICIPANT]:** Okay, there are those people who are understanding that they get to agree it and others won't.

**[INTERVIEWER]:** Why do you think there others who won't be for it?

**[PARTICIPANT]:** Some guys are uneducated that's why, they might find it hard to understand such things.

**[INTERVIEWER]:** Okay, do you see any positive outcomes and benefits of PNS?

**[PARTICIPANT]:** It reduces the spread of HIV and the partners get to know their status...

**[INTERVIEWER]:** Any other benefit?

**[PARTICIPANT]:** No that's all I have.

**[INTERVIEWER]:** Okay, how will a person who tests negative benefit from it?

**[PARTICIPANT]:** They immediately start PrEP and will be protected.

**[INTERVIEWER]:** What about the one who will be found to be HIV positive?

**[PARTICIPANT]:** He will be offered counselling and get to start the treatment.

**[INTERVIEWER]:** Okay, do you think there are any social benefits?

**[PARTICIPANT]:** No, none.

**[INTERVIEWER]:** Any other strategy that you think would be easier for us to reach out to the partners, you mentioned that the clients should be given a chance to chip in their ideas. Do you have any?

**[PARTICIPANT]:** No, I think for me using the OST kit is the best.

**[INTERVIEWER]:** Was PNS introduced the same day that you took the HIV test?

**[PARTICIPANT]:** Yes it was.

**[INTERVIEWER]:** What was you're feeling about that? Do you think it was okay for it to be introduced the same day you knew about your status or you would recommend otherwise?

**[PARTICIPANT]:** Okay I wasn't introduced to PNS on the same day I got to know about my HIV status, it was a day after. It was okay with me.

**[INTERVIEWER]:** Do you have any communication strategy on how to approach the partners when we calling them, We always call and say "hello how are you I'm calling from [RESEARCH_INSTITUTION] I'm informing you that you may be at a risk of being infected with HIV we are asking you to come over and get tested at our institution" how would you put it, let's say you are health care service provider what would tell the client you just called?

**[PARTICIPANT]:** I think its fine.

**[INTERVIEWER]:** Okay, think about it this way you called a partner that you were in a relationship with and he blocked now think about a counsellor calling a total stranger?

**[PARTICIPANT]:** it must be hard

**[INTERVIEWER]:** it's hard?

**[PARTICIPANT]:** yeah very hard, by the do you that calling thing work? Are you getting partners through calling?

**[INTERVIEWER]:** (*chuckling*) I'll tell you after the interview.

**[PARTICIPANT]:** (*Chuckling)* it's okay.

**[INTERVIEWER]:** It just depends on the partner on the other end, a good number have reported and others not successful

**[PARTICIPANT]:** Okay. That's true calling someone with that message.

**[INTERVIEWER]:** How should we put it?

**[PARTICIPANT]:** I think its okay.

**[INTERVIEWER]:** How can we build confidentiality and trust when using the health care service provider strategy?

**[PARTICIPANT]:** Just by communicating well and being nice that person and understand will trust you.

**[INTERVIEWER]:** Do you have any recommendations for implementing PNS for GBT in Kenya?

**[PARTICIPANT]:** No, I don't.

**[INTERVIEWER]:** Is there anything else you would like to add?

**[PARTICIPANT]:** No, I have nothing else to add.

**[INTERVIEWER]:** thank you very much for coming and dedicating your time to share your views and experiences. I truly appreciate.
