# Supplementary material for: Peer-assisted HIV partner notification services to strengthen index partner testing for newly diagnosed men who have sex with men in coastal Kenya
Source: PLoS One. 2025 Oct 7;20(10):e0333707. doi: 10.1371/journal.pone.0333707 (PMC12503256; doi:10.1371/journal.pone.0333707)
Supplement: S3 Appendix — (ZIP) [file pone.0333707.s003.zip › Deidentified IDI Transcript_1272.docx]

**Participant characteristics:**

Age: 20-24

Sexuality: Bisexual

Education level: Primary

Days between enrollment and IDI: 63 days

Mobilization strategy: OST

Final PNS Strategy: HCP/PM

**Partners identified: 6**

**[INTERVIEWER]:** Welcome again, It's the end of the month, [DATE] at [CLINIC_A]. As you remember we assigned to you a unique number for the purpose of confidentiality, therefore your number is 1272 Right now, I will go back to the purpose of this study so that we can be at per with each other.

**[PARTICIPANT]**: Ok

**[INTERVIEWER]:** We are currently conducting a study known as PNS, ie. Notifying partners of those infected with HIV so that they can also get tested and know their HIV status. Therefore what we are doing in the study is that, we have peer educators/mobilisers who are distributing the OST kits to their peers, you test yourself at your preferred location then come to the clinic with the kits and other study procedures are explained to you, once you accept to join the study other procedures follow.

**[PARTICIPANT]:** Mmmmh

**[INTERVIEWER]:** Ok therefore PNS is a service that has been going on for a while now, anyone who tests HIV positive we would like their sexual partners to also get tested and know their status, for those who are HIV negative we can find ways that are suitable for them to avoid HIV infection.

**[PARTICIPANT]**: Ok

**[INTERVIEWER]:** We have different methods of PNS, one of them is that the service provider can help you notify your partner so that they can also get tested and know their status. In summary PNS has been done for the general population but we are yet to know how we can go about delivering this service to the population that is most at risk of HIV infection. In our discussion today those at risk that we are focusing on are MSM, GAY, or even Transgender women. What iam trying to say we don't know how we can reach their partners for testing and that is why we thought to ourselves what better way we can do this than working together with the MSM, gay and Transgender themselves.

**[PARTICIPANT]:** Ooooh OK.

**[INTERVIEWER]:** Therefore, that is one of our objectives of this study

**[PARTICIPANT]:** OK

**[INTERVIEWER]:** So, tell me, how have you been ever since the last time you were here?

**[PARTICIPANT]:** Iam grateful, iam getting better compared to the last time I was here. Iam taking my medication well and things are just fine.

**[INTERVIEWER]:** Have you had any challenges

**[PARTICIPANT]**: Not at all, I take my medication go on with my normal duties. I eat well and that's all to it

**[INTERVIEWER]**: Have you possibly been able to disclose your status to anyone?

**[PARTICIPANT]:** There are others that I tell them but not the ones that know me. You may tell someone that you know, and they start going around gossiping to others.

**[INTERVIEWER]:** What do you mean when you say, "Not those that know you? "Please make me understand.

**[PARTICIPANT]:** You know there are those that you may tell, and the information gets home to my relatives.

**[INTERVIEWER]:** Therefore, there are some that you have talked to about your status.

**[PARTICIPANT]**: Yes

**[INTERVIEWER]:** Has there been any challenges so far?

**[PARTICIPANT]:** About?

**[INTERVIEWER]**: I mean any challenge or difficulty ever since you started ART

**[PARTICIPANT]**: At the beginning yes but right now I am doing much better, I didn't have a job and was not able to feed well. I avoid much stress as well right now since I know my condition and I want to be healthy. Right now, I am a fisherman and I solely depend on the sea. At [LOCATION_B] (A place here in [CITY_A] where they do fishing) at the moment there is no fish but hopefully August things will be good.

**[INTERVIEWER]**: You look much better now indeed!

**[PARTICIPANT]**: Yes, before I used to desire a certain meal and it was difficult for me but now, I eat what I want. Things are much better for me.

**[INTERVIEWER]:** Oooh Yes, your skin is even glowing

**[PARTICIPANT]:** Ha ha ha ha... this is nothing better things are on the way, I will come here again one of these days and all of you will be amazed. HAHAHA...

**[INTERVIEWER]:** Ok let's go ahead then. What made you come here for testing the other time?

**[PARTICIPANT]**: You know, I started getting ill but didn't know what was wrong with my health. I was always sleepy, no appetite at all I was only drinking water. I was afraid to come for testing, one time I visited [MOBILIZER_A] ([MOBILIZER_A] is one of the mobilisers) and he looked at my condition and advised me to come for testing, but I was not convinced at all. In a period of two weeks he came to me again and told me it was important to come to the hospital and seek help, that's when I gave myself courage and came along with him to this place and was tested for HIV.

**[INTERVIEWER]:** Ok, how frequently did you test for HIV before

**[PARTICIPANT]:** I had only tested twice my entire life, and this was my third time....

**[INTERVIEWER]:** When was the last time you got tested before you came here?

**[PARTICIPANT]:** It has been like three years now. I was tested with health providers who were wandering around in the community offering HIV services. I was with my partner at that time a woman and the results were negative. I was ok

**[INTERVIEWER]**: So, this is three years, back right?

**[PARTICIPANT]:** Its four years if I am not wrong.

**[INTERVIEWER]:** Ok, what do you think put you at risk of HIV infection?

**[PARTICIPANT]:** Having sex......

**[INTERVIEWER]:** What do you mean?

**[PARTICIPANT]:** Most of the time when I had sex, I did not use a condom. I assumed that my partner was ok but then that's just how it is. Sometimes also I experienced a condom burst

**[INTERVIEWER]:** Oooh OK, it's possible that you didn't have enough information but once one experiences a condom burst, they can get help. It maybe of no use with your situation now but when you have enough information you can share and help others out there. So here is the thing, when one experiences condom burst they should come to the hospital as soon as they can ie within 72 hours since the incident happen and will undergo an HIV test, once they are negative they will be given medication known as PEP that will reduce the chances of infection at all the other partner was HIV infected.

**[PARTICIPANT]**: This is good information, other people I know can benefit from this. I also heard there are those medication that one can take and have sex without protection with a person who is infected and still be ok.

**[INTERVIEWER]**: Yes, those are called PREP.

**[PARTICIPANT]:** I will bring my current partner who we live together. He is a male partner. I believe PREP is what he needs right now. We have not tested together yet so I want him to get tested and depending on the results he can start the ARV's like me or take PREP.

**[INTERVIEWER]:** That will be a good thing. Ok taking you back a little bit you just told me that there is a peer mobiliser who advised you to come for testing, what did he tell you that motivated you to agree to come with him and know your status

**[PARTICIPANT]:** I think its just myself. He followed me like twice, but I was not ready. I didn't know that he was working with you people here. He told me it was important for me to get tested and he brought me an OST kit. I did the test at home and then afterwards he brought me here

**[INTERVIEWER]:** What do you have to say about the discussion you had with the peer mobiliser

**[PARTICIPANT]**: He told me about quite several things, he told me that I should not stop taking ARV's, he advised that I should be aware of drinking because the drugs don't respond well with Alcohol. He was of much help and kept motivating me all the time. I even use to leave my drugs at his house since it was not safe to have the drugs with me at my own house.

**[INTERVIEWER]:** Its evident that he has been so supportive.

**[PARTICIPANT]:** Of course, look at me now. I am much better.

**[INTERVIEWER]**: Ok, just like you Men who have sex with other men don't frequently get tested. You are a good example if you may allow me to say, you stayed for over four years without testing for HIV. What do you think we can do so that the MSM, GAY or even Transgender can frequently test for HIV?

**[PARTICIPANT]:** If you allow me, give me like two weeks then I will have enough money. I will then be able to go after them one by one and bring them here for testing, but right now I don't have enough so when I waste it on transport to go find them, I may be left with nothing.

**[INTERVIEWER]**: Perhaps you did not understand me, I was not talking about your partners. Normally the MSMS gay or even Transgender women don't test for HIV as frequent, then what would we do so that these people can come out in large numbers and test for HIV. What ideas do you have that can possibly help to convince or even motivate them to test for HIV?

**[PARTICIPANT]:** Oooh Ok, this should be done with patience because it's not easy. You can use people who are familiar with them, someone who understands them better to convince them to test. I believe that would be very important.

**[INTERVIEWER]:** Ok, this is helpful. Let's get back to when you tested at first using the OST. What was going through your mind when u first saw those two red lines on the kit.

**[PARTICIPANT]:** At first, I thought I saw only one line, then I saw the second line that's when my heart sunk and felt so sad. I thought that was it for me, everything was shuttered in a moment. After some time, I was more motivated, the mobiliser helped me a lot. I told myself I could not cry over spilt milk, I can't change it therefore I should fine ways of dealing with the situation instead, that's when I came here.

**[INTERVIEWER]:** I can imagine what you went through, it was so brave of you to come here and finally started ART. Did you start your medication immediately when you came here for the first time?

**[PARTICIPANT]:** I started my medication immediately

**[INTERVIEWER]:** I believe you met with the health provider and talked about a few things, could you tell me about the discussion you had with the counsellor

**[PARTICIPANT]**: It was ok, he talked of so many things but most importantly he insisted that I should never stop taking my drugs and incase of any challenges I could come in to the hospital for further support and counselling.

**[INTERVIEWER]:** Did you feel supported and much at ease afterwards?

**[PARTICIPANT]**: Yes, everyone around here treated me so well. We even discussed on my smoking. I smoke bhang a lot something that I have tried to work on ever since, now I only smoke one stick of bhang in a day compared to before.

**[INTERVIEWER]:** Ok now we will talk about your partners that you mentioned and partner notification. When was partner notification introduced to you?

**[PARTICIPANT]**: we did not discuss about my partners immediately, but he told me of the do's and don'ts of taking ARV's. I came back on a different occasion and we were able to discuss about several partners.

**[INTERVIEWER]**: Ok, I remember having that discussion with you. Most of your partners didn't have contact information but you were able to give a description to our mobiliser and lucky enough we could get most of them to come for testing, but it was rather difficult for us to get the female partners. I thought you should know, and confidentiality was maintained in every aspect.

**[PARTICIPANT]**: ok

**[INTERVIEWER]**: has anything changed so far in terms of your relationships?

**[PARTICIPANT]**: Personally, I am the one who is trying to avoid my partners because iam certain that one of them infected me. I say my greetings when we meet but its just cordial for now.

**[INTERVIEWER]**: Ok I understood you. Apart from the partners that I mentioned we were able to reach, is there any other that maybe you forgot to mention before and would like us to discuss about it now?

**[PARTICIPANT]**: No, we exhausted all the partners

**[INTERVIEWER]:** Ok, you mentioned earlier that there are people that you were able to disclose your status to, what relationship do you share with them?

**[PARTICIPANT]**: They are my colleagues, we fish together.

**[INTERVIEWER]:** And what was their reaction when you told them that you were HIV positive?

**[PARTICIPANT]:** They are ok

**[INTERVIEWER]:** Has your relationship with your colleagues change in ever since

**[PARTICIPANT]**: Not at all, I am the captain there so they answer to me.

**[INTERVIEWER]:** So, you have only disclosed to your colleagues

**[PARTICIPANT]**: Yes.

**[INTERVIEWER]:** Now that you are aware that some of your partners were able to come to the clinic for testing, has anything happened as a result of Partner notification?

**[PARTICIPANT]**: Not at all. Nothing extraordinary has happened, everything is normal.

**[INTERVIEWER]:** Generally, when offering PNS we normally discuss about sexual partners of the index within the past one year, what was going through your mind while we were discussing about your partners back then, what made you be open about it?

**[PARTICIPANT]:** I didn't have a reason to hide it from you because its important for my partners to also get tested and know their status.

**[INTERVIEWER]:** Ok I hear you. Now we are going to talk about the challenges of Partner notification. In your own opinion what do you think about PNS

**[PARTICIPANT]**: I believe it's a good thing, since I already know my status, its also good for my partners to get tested. You never know amongst them there are those that I may have also infected unknowingly. That's why I feel PNS is something that should be ongoing and therefore offered to everyone in general.

**[INTERVIEWER]:** OK what we are trying to achieve in this study is that we would love to offer PNS services to MSM, GAY or even transgender in future therefore we trying to get ideas on how we can make PNS effective to this group of people. What can you tell us about the same? Do you have any ideas that maybe be of help in making PNS a success?

**[PARTICIPANT]**: It will depend on each person individually, there are those that will listen once you advise them on a certain situation. So, I think it's how an individual will perceive

**[INTERVIEWER]:** Ok, do you foresee any challenges in making PNS effective among MSM?

**[PARTICIPANT]:** No, I don't see any challenges whatsoever

**[INTERVIEWER]:** Ok, so when we discussed about your partners there were different ways that we mentioned that we can use to reach them, and you specifically choose the method that suits best for each partner.

**[PARTICIPANT]:** Yes, I remember

**[INTERVIEWER]:** I will now take you back to different methods that partner notification can be done. There is the index patient, we have a peer mobiliser, and there is a health provider, one way of PNS is that the health provider to make an anonymous phone call to the partners and invite them for testing, that is of course with your consent, Second way is that the peer mobiliser can offer an OST kit to the partner...

**[PARTICIPANT]:** Ooh yes, I just remember I would need one more OST so that I can take it to my current partner for testing.

**[INTERVIEWER]:** Ok, no problem I will give you after our discussion. Let's get back to different methods of PNS, Thirdly the peer mobiliser can go to a hotspot where your partners mostly hang out, they would distribute OST to people at the hotspots but in mind targeting that partner...

**[PARTICIPANT]:** Now that's the best one

**[INTERVIEWER]**: Oh really, the fourth one is that the health provider can help you disclose to your partner, this can be done through couple counselling and testing. In this situation you can bring your partner for testing assuming that you don't know your status and that you are testing with them for the first time. Another way is that the peer mobiliser can help you to invite your partner for testing. Lastly, we can give you the OST kit so that you can give it to your partner/s and afterwards they can come to the clinic for a confirmation test. Are we together?

**[PARTICIPANT]:** Yes. I understand what you are saying

**[INTERVIEWER]:** Therefore, among all this different methods that we just discussed which one would you prefer?

**[PARTICIPANT]:** I would rather take the OST kits and give them to my partner/s

**[INTERVIEWER]:** Ok, you told me earlier that PNS was introduced later. Do you think that was favorable or would it have been better if you discussed about your sexual partners on the same day of diagnosis?

**[PARTICIPANT]:** In my opinion it's better to discuss it later. On the day of diagnosis so many things are happening, a lot was on my mind and I could not think straight. Waiting for a few days later is the best thing to do

**[INTERVIEWER]:** Ok so what you are saying is that one needs to be given time

**[PARTICIPANT]:** Yes, at that time one is not in their right senses. You feel like the whole world has crumbled on you and therefore not in the right state of mind to give you the right information that you need.

**[INTERVIEWER]:** We almost coming towards the end of our discussion. I would like you to tell me exactly how you would want us to tell your partner when we make that phone call to invite them for testing.

**[PARTICIPANT]:** I think that its hard to make anyone come here actually if they don't know you at all. The best thing to do is first go and meet them and create a good rapport then afterwards invite them to the clinic.

**[INTERVIEWER]:** Ok, is there any thing that you would like to share with us that can help in making PNS success, ideas or suggestions.

**[PARTICIPANT]:** I am just grateful for the good work you people are doing

[INTERVIEWER]: Thank you so much, we have just concluded our discussion. We are done with all our procedures regarding the study, but we will be here to support you. Thank you for taking your time to participate in this study, we are totally privileged to have you. Ahsante sana.
