# Supplementary material for: Peer-assisted HIV partner notification services to strengthen index partner testing for newly diagnosed men who have sex with men in coastal Kenya
Source: PLoS One. 2025 Oct 7;20(10):e0333707. doi: 10.1371/journal.pone.0333707 (PMC12503256; doi:10.1371/journal.pone.0333707)
Supplement: S3 Appendix — (ZIP) [file pone.0333707.s003.zip › Deidentified IDI Transcript_1327.docx]

**Participant characteristics:**

Age: 40-44

Sexuality: Gay

Education level: Secondary

Days between enrollment and IDI: 44 days

Mobilization strategy: OST

Final PNS Strategy: HCP/PM

**Partners identified: 1**

**[INTERVIEWER]**: We will have to be audible enough to make sure what we discuss will be well recorded,

**[PARTICIPANT]:** Thanks

**[INTERVIEWER]:** Welcome today for the interview, today is [DATE] and thanks for fulfilling our request to meet you.

**[PARTICIPANT]**: Thanks

**[INTERVIEWER]:** we would want to have an interview with you in our offices here in [CITY_A] about PNS where your PNS number is 1327.

**[PARTICIPANT]**: Yes

**[INTERVIEWER]**: we start by explaining what the study was all about, the study was called PNS (partner notification services) it's a study where the partners of HIV positive people are tested to know their HIV status, where if one is HIV positive it's important for them to start early ART treatment and for those who will have not been infected there are ways to make them stay HIV negative throughout. For the infected people we call them index clients just like you who tested positive, through volunteerism we ask you to discuss your partners so that we can let your partners know their HIV status. There are different methods we can use to successfully do PNS or to reach to your sexual partners. So, we will use different methods to get your sexual partners to come for testing and you will give us the right method by yourself which you think its suitable to communicate with your sexual partners, which every sexual partner may have his own method of contacting him/her and it depends on how we will agree here. PNS has been going on long time ago where if one test HIV we could do a follow up to make sure we bring the sexual partners for testing but it was only that we didn't have better means to reach to those people who are at a high risk of getting HIV virus. Mostly we would like to know more especially from men who have sex with other men and it's the main aim of this study to know from you, MSM how we can successful do PNS or to let those MSM know their partners who are HIV infected and this method will not affect them in any way for those people who will have given us the information.

**[INTERVIEWER]:** How do you feel today.

**[PARTICIPANT]**: There is a very big different since that time I tested HIV positive, because that time I could be having fever always before I started the ART, my body was very weak, joint pains, I could have head ache, back ache but since I started ART all those problems stopped and because I am doing well with drugs and all the problems I faced before since I started drugs are no longer there.

**[INTERVIEWER]:** Happy to hear this, are there any other challenges you have faced since you started ART?

**[PARTICIPANT]:** No, I can't say they are challenges as such, because if I wake up in the morning I take my drugs and after that I don't see any problem, in fact I didn't experience any side effects with these drugs.so I can't say I had any problem with the drugs.

**[INTERVIEWER]**: The day you tested HIV for the first time is there any reason which made you to know your HIV status?

**[PARTICIPANT]**: There are two to three things which made me test, you know most of the time you get examples from people, you may here people have died and maybe if u ask people they tell you he/she refused to take drugs and sometimes if you look your sexual life, today you may use a condom ,tomorrow you don't use ,you expose yourself to sharp objects meaning your reckless sometime that's why I saw why people go for HIV testing and me I don't get tested, despite that most of the time I get drunk and have sex with my partner without protection. it's when I got the urge to go for testing. So, I told another brother that I have decided to go for HIV testing, that's why I came here and when I tested, I got myself to have been HIV infected.

**[INTERVIEWER]**: So, before you came here for testing, how long had you stayed before testing again?

**[PARTICIPANT]**: I had stayed for almost four months before I came again here for testing.

**[INTERVIEWER]:** And what was your previous results before you came here for testing?

**[PARTICIPANT]**: The results were HIV negative.

**[INTERVIEWER]**: So, your HIV results were okay?

**[PARTICIPANT]**: yes, they were okay.

**[INTERVIEWER]**: So, was it your first time to realize your HIV positive?

**[PARTICIPANT]**: Yes, it was my first time to realize I was HIV infected?

**[INTERVIEWER]**? Is it possible that you met with peer mobilisers? because we have mobilizers who we work together in the field. If you met them are they the ones who directed you to the clinic or they gave you the oral self- test kits?

**[PARTICIPANT]**: I got a person who directed me to this place, he told me it will be better if I come to [RESEARCH_INSTITUTION] where I will be attended best.

**[INTERVIEWER]**: can you please share with me, what you discussed and what convinced you to come at [RESEARCH_INSTITUTION].

**[PARTICIPANT]**: Yes I explained to him my worries, what I want, because I didn't want to hide anything on him because most of the time if you get a person, you explain to him your personal issues although he might not be in a position to give you fully support but he can give you ideas, direct you to a better place which if you go to that place your problem will be easily solved that is where you feel the door for success opens, so when I explained to him that am worried of something and I doubt my health status although am not 100% sure but if I go and test its when I will know the way to go and he directed and explained to me about [RESEARCH_INSTITUTION] and for sure I came and tested and the results were positive., so when I tested I positive I thank GOD am now doing well.

**[INTERVIEWER]**: Could you please explain in detail what you discussed with him.

**[PARTICIPANT]**: Just briefly speaking, he asked me if I understand what it means for a person who has tested HIV positive. what are the things one should avoid so that he could not be infected and if he is infected what he should do? That's why we discussed a lot, for me the knowledge I had is to avoid sharp objects e.g. razor blades, needles, pins, having unprotected sex, those are the questions he asked me I answered him. For pregnant women I have no idea but those things which surround me are the ones I explained to him and he asked for all those I have explained to him are there some I suspect they could have exposed you, for sure I didn't hide anything so I told him the truth that sometimes I might be drinking, so he told me there is a big possibility that I may be okay or I may not be okay but there is no any problem in HIV testing. you just come to the hospital you be tested after testing, you may be okay but you never know.so my big worry was and the explanation he gave me already gave me relief in my heart that's why I said to myself anyway there is life after one test HIV positive but although people have denial it's as if people are seeing you and they may ask what did we tell(blame game) ,something like that but because I have my own life and the only person I asked was the health worker and they have confidentiality with peoples private information then that's what made me decide to come without worries.

**[INTERVIEWER]**: So, the peer mobiliser assured to you about confidentiality that's why you decided to open up?

**[PARTICIPANT]**: Yes, that's what made me open up and share with him.

**[INTERVIEWER]**: yes, it's good to hear that from you. So, did you bring the oral self-test kit when you came on the first day which you tested using your mouth?

**[PARTICIPANT]**: yes

**[INTERVIEWER]**: Maybe did he explain to you how the oral self-test it's being used?

**[PARTICIPANT]:** yes, he explained to me that the self-test gives results if you test HIV using your saliva, so I used it and after like 30 minutes not exactly sure but it showed results i.e. two lines that is when the peer mobiliser confirmed to me that am HIV positive.

**[INTERVIEWER]**: Was there any importance of confirming that with the blood test?

**[PARTICIPANT]**: Yes, it was important to confirm with the blood test so that we can fully be sure.

**[INTERVIEWER]**: That's good.

**[PARTICIPANT]**: Yes, because we could not rely on only the first test.

**[INTERVIEWER]**: Yes, that's good because we inform people after testing with the oral self-test they must come to the clinic for confirmatory before doing anything with client, because we didn't witness the person using the self-test, maybe he didn't follow the correct instructions of testing that's why it's very important to confirm with the blood test before doing anything with the client.

**[PARTICIPANT]**: He confirmed with the blood test which tested the same as the self-test.

**[INTERVIEWER]**: That's okay.

Did the discussion with the peer mobiliser assist you in anyway?

**[PARTICIPANT]**: Yes it really assisted me because ,when we are in the villages we find it hard or difficult to make certain decision because it's easy for people to know your HIV status where sometimes it's shameful even to your family members ,so the peer mobiliser played a very big role by giving me information which makes me understand the current situation and which it's easy to disclose my status, when you are left without been given any information by the mobiliser which will make you aware that you are a human being and your very important ,people need you and you can still do something and if your thinking is still about in the community you will continue hiding, and if you hide you will not be hiding yourself but you will be threatening your health which could be easy if you could open up to the mobiliser, because I opened up to the mobiliser ,he had the courage to explain to me what is going on ,which I felt supported that's why I took the step to seek for treatment through him.

**[INTERVIEWER]**: Oh, that's very good, it shows he really helped you, and that's are our goals. we give information that's why people can understand us, because most people in the village don't have information which makes it very hard to make decision on health issues.

How can we give morale to MSMs to come and seek testing services, because majority are very shy and afraid to go test their HIV status?

**[PARTICIPANT]**: I think the mobiliser may seem strange to MSM and it might be very hard to face and talk to the MSM but let's say for example I meet a stranger it will be very difficult of me explaining to him or it will be very difficult to begin what you wanted to explain/inform him but you will find it easy to a person you are used to or you had already interacted/met before. It becomes very easy for you to sit down and discuss a lot of things and then you will see how you switch the story and explain to him in a good way that even the person who will be being explained will understand, you know living without knowing what your doing is like having a phone which you don't know if it has credit or it doesn't have ,it waits until you want to call is when your told the line doesn't have sufficient credit.so its good even if he is okay but at least he is your very close friend and maybe you have undergone HIV testing and know your infected you will know what to do and if you are uninfected also you will know what to do provided you underwent HIV testing it becomes very easy even when you meet in the village or hot spots taking alcohol you will just explain to him that we can visit a place where we will find a lady called [STAFF_B] she is also a person who understands very well and that will be our confidentiality between me and you but if you find that's he will be HIV negative you will know what to tell him but if he turns HIV positive I will tell him that am also HIV positive and I started medications thus am controlling my life well and everything is going on well. Instead of just seated and not knowing what's deteriorating your health. So, I think by just using people among us has more impact although peer mobilisers are more capable because they are more educated.so there is a small challenge but I think if the mobilisers will use people like us to reach the clients to their end, then their work becomes much easier.

**[INTERVIEWER]**: ooh, so you are telling me that the mobiliser can't reach to new clients until they laisse with you?

**[PARTICIPANT]**: Yes, unless they know each other is when they will know how they will handle each other.

**[INTERVIEWER]**: during the first time you were tested and found you were HIV positive, what was going on in your mind?

**[PARTICIPANT]**: For the first 15 to 30 minutes of knowing my HIV results, there was a big shock, just trying to figure out that my life is no more and am leaving the world, so you think your friends will be going on well and yourself you will be just counting of your days on earth. So I had such thoughts although I remembered the peer mobiliser what he told me which consoled me so I reminded myself that people take drugs and they are living ,why should I stress myself but even when you think something like that but you will again remember that you are infected ,how will you know I will live a good life like them? You ask yourself so many questions that, if people talk about medication are they talking the truth about the drugs really work or they are just talking to make us happy then life will be short us we think? I faced the challenges and I had a lot of questions until at the end I was stressed (mixed up in my head) I just gave up and decided what come what may because it had already happened I had no any other options.

**[INTERVIEWER]**: so, you had no any other option?

**[PARTICIPANT]**: yes, that's what I felt at first, but within one to two hours going on after I had talked to health workers, I went to the adherence room and I listened what they explained to me then I got a lot of hopes.

**[INTERVIEWER]**? Yes

**[PARTICIPANT]**: Because they explained to me with good examples and everything that is supposed to happen and trying to compare from all doctors what they are trying to tell me and it's when I realized all what they were talking about were the same issues. So, when I was started on treatment I experienced what exactly what they were talking was now happening to me.

**[INTERVIEWER]**: Did you start drugs just immediately or you started later?

**[PARTICIPANT]**: no, I started drugs after two weeks because on the first time when I came I was diagnosed with TB so they couldn't start me on ART and TB drugs at the same time so they had to start me on anti TB drugs then after two weeks they came and initiated me with the ARVs.

**[INTERVIEWER]**: yes, I now understand you, so now we will discuss about your sexual partners which you mentioned on the first day we were together. You mentioned two sexual partners, a man and a woman, so you will explain to me your experience with PNS, disclosing your HIV status to your sexual partners or we are disclosing your status to your sexual partners for them to come and know their HIV status. On the first day you came could you remember what you discussed about PNS.

**[PARTICIPANT]**: about disclosing one's status to sexual partners.

**[INTERVIEWER]**: yes

**[PARTICIPANT]**: I think through phone calls could be the best option, because sometimes you may approach a person and there may be a blame game, which I would be who have will made all this happen, which may result to a conflict where one will think that i have wasted his life which he thinks he will soon die, so there will be no good dialogue but if we use the health worker to contact the sexual partners through phone calls, to me it will be the safest way of getting the sexual partners.

**[INTERVIEWER]:** So, you think this will be the safest way of getting your sexual partners come for testing?

**[PARTICIPANT]:** yes, I think this is the best method which would have avoided much drummer, and if he will respond in a good way then it's when I will also intervene and see how we can help each other but at first the health worker should start.

**[INTERVIEWER]**: you mentioned to me about your two sexual partners, has any one of them approached and told you maybe they were contacted through phone? Which they were called and requested to have HIV test? Is there anyone who opened to you?

**[PARTICIPANT]**: No, because it's very rare to meet them, one of them travelled and the other one I have not met with him, you know according to what the doctor told me about TB treatment, mostly they require one to be patient.

**[INTERVIEWER]**: yes

**[PARTICIPANT]:** that's why I have not met them because most of the time am indoors, this cold season I have chosen not to travel, most of the time I could go out but dew to the chest problem which has made me relax indoors most of the time. most of the time when you called through the phone and maybe he was your sexual partners you sometimes must ignore because sometimes he may persuade you and break instructions from the chest treatment, so you see, you avoid that until you complete the required treatment and instructions.so this has been my challenge, even its hard for me to know if any was contacted through phone.

**[INTERVIEWER]:** it's okay, I have understood you. You mentioned that one of your sexual partners has travelled?

**[PARTICIPANT]**: yes, the female partner.

**[INTERVIEWER]**: she is not here [CITY_A]?

**[PARTICIPANT]:** yes, she travelled but it's not that she will never come back but there is a problem which came out about land issues, so she just went to work on that then she will be back.

**[INTERVIEWER]:** so, she is outside [CITY_A] for now?

**[PARTICIPANT]**? Yes, but in two days' time she will back because she went one week back, yes there is a person who they communicate through phone so we met yesterday that's why he told me she will be back the day after tomorrow.

**[INTERVIEWER]**? Okay then will try and make sure we continue looking for her through phone calls.

Have you ever disclosed your HIV status to anybody so far?

**[PARTICIPANT]**: yes , I have a brother who is like my next of keen ,he is the one who knows almost everything about me, he has been motivating me, helping me with everything, because I have different needs and am supposed to work but sometimes I can't work so he has been supporting me a lot ,he is the only person I have disclosed my status to him ,the others am still thinking who I can tell although they are my brothers, but I am still assessing who can show confidentiality that's why am still selecting the best, so I don't know much with the others but to me with this situation I see this must be a big secret so for the one I will have disclosed to he must show me he will keep my secret and be caring like health workers but the rest I don't think they will be having any help to me because I don't want any person to remind me of the past, suppose I get five people who take care of me but out them I don't hear anything bad it will be okay.

**[INTERVIEWER]:** So, for now you have only one brother who you have disclosed to

**[PARTICIPANT]:** yes

**[INTERVIEWER]:** He is the only person who is very close to you,

**[PARTICIPANT]:** yes, he is my closest person

**[INTERVIEWER]**: how is your relationship since you disclosed to him?

**[PARTICIPANT]:** in fact, he has become very much closer and more caring than before.

**[INTERVIEWER]**: He has been close to you than before. He is the one giving you morale to continue living better and making sure you always take medication.

**[PARTICIPANT]**. Yes, he makes sure I take much care of myself, and he has accepted how I am.

**[INTERVIEWER]:** we say if a person is HIV infected, it doesn't mean it's the end of life but it means it's a beginning of a new life. Nowadays people take drugs and live a very normal life, in fact people die due to other illnesses, what we want as health care givers we make sure people don't die due to HIV virus, we want people to take their drugs always as we know in Kenya if people are started on ART early, their virus will be suppressed completely which will be very hard to infect another person. The virus in the body will not be active, so you will be like any other person who is not HIV infected.

**[PARTICIPANT]**.so you will just be normal?

**[INTERVIEWER]**: yes, you will just be like a normal person.

So, among the sexual partners, there are some who maybe were told your HIV status or they don't know your HIV status, of which you are not aware, if you compare the relationship with them is there any change or you never know as you have told me that you have not been meeting with them?

**[PARTICIPANT]**: I don't know and it's because I see things stagnant always but maybe when I complete these things concerning drugs is when I will know how it's going on, mostly its brought about visiting each other, through phone communication that's when you will know how people are doing.

**[INTERVIEWER]:** it's okay. So if I try to remember at first PNS is something which used to happen long time ago, if you come to the hospital we could do a follow up of your sexual partners to get tested, so for now the Kenya National policy they recommend that we talk about partners for a person he has been with for the past one year, for any person they have had sex either they had long term or short term relationship provided they have had sex ,so that person has to be notified.so we just did the same discussion with you about your sexual partners the first time you came to the clinic. So, if I can ask how was the discussion which made you open about your partners.

**[PARTICIPANT]**: yes, there was a feeling, you know this is a disease which is a result of infecting each other, so if you think about the chain, I think to a person who cares, it really feels, if this disease is transmitted from others and those people I have had unprotected sex with them and maybe by that time I was already infected and so it comes the thinking that he might be infected just like me, and the reason is that I know my status and the sexual partner doesn't know.

**[INTERVIEWER]**: yes

**[PARTICIPANT]:** so, the other person will be in the dark side, doesn't know anything.

**[INTERVIEWER]:** yes true.

**[PARTICIPANT]:** so, you have to act in humanity way, that this other person I have to make sure he gets assistant even if it's giving out his phone number to the health worker so that he can get assistant by knowing his HIV status, if he is positive will be together but if he is luck he knows how to take care of himself from being infected and we can know more how we will be meeting. And for that time maybe he will not be aware of my HIV status so it is very important to know his HIV status, so it really helps, especially for those who don't know will unfortunately will come and know their status and be assisted for him to know how to do.

**[INTERVIEWER]:** yes. You mentioned two sexual partners on the first day?

**[PARTICIPANT]**: yes

**[INTERVIEWER]:** what do you think made it easier for you to discuss about the two sexual partners so that we can look for them?

**[PARTICIPANT]**? Because I had known my HIV status so I didn't know what could be their status, that's why I mentioned them.

**[INTERVIEWER]:** sometimes we talk about sexual partners and most of the time it's hard to remember all the sexual partners, say the sexual partners are a lot I can't remember them but according to your case it was much easier because they were only two which you remembered and you gave us their phone numbers, sometimes if one can't remember all it becomes a challenge and doesn't have phone numbers so contacting them becomes very difficult.

**[PARTICIPANT]**: yes

**[INTERVIEWER]:** were there any sexual partners apart from the two you didn't mention last time because you were not free?

**[PARTICIPANT]**: No. they are the only two, I don't have many sexual partners so it was easy to mention the two and I had their phone numbers.

**[INTERVIEWER]:** it's okay, we are going slowly but almost completing this interview.

**[PARTICIPANT]:** its fine.

**[INTERVIEWER]**: According to you that we gave you the PNS services and according to you the method we used to you it was the right one, how can you talk about this PNS services if other people could accept it or it's a service it can work.

**[PARTICIPANT]**: it's a service which can work but according to me it cannot apply to all people because we are born differently, there are other people if they get a bad thing they could wish there friends also to get the bad thing and vice versa, there are others who will sit in front of you and ask themselves why can I mention my fellow male partner, how will people look at me, he will already started putting obstacles which will make him that he can't work with PNS services and if he works with it he will have hidden a lot of things and its very hide things which sometimes it comes back to you and the service will never assist you. So, this service is good and according to my view I would like everybody who will pass through PNS like me should do PNS services and make it successfully with all his effort because being open will make all of us overcome this problem and hiding other information or afraid or saying I will do this PNS voluntarily I don't think if I will use the microphone, yes, it's okay but at the end of the day will you keep confidentiality? And there is a lot of secrets and I don't think if there will be any problem because my information will assist so many people and they will know if they will face the problem in which way, so I recommend PNS on my opinion and those which will maybe not like the service they must understood its importance if something happens it happens with a reason and I don't see any problem that their other things we can talk others we can't talk.

**[INTERVIEWER]**: so, we say if water pours it's never collected.

**[PARTICIPANT]**: yes

**[INTERVIEWER]:** so, we must look others means to deal with the situation how it has occurred.

So PNS is what I have told it was being carried out but its only that we don't have proper guidelines on to conduct it on MSM and it's the main object of this study, there is recommendation that PNS to be done on MSM what is your opinion about this issue? do you think there will be challenges that we will face to make this service successful to MSM?

**[PARTICIPANT]**: MSMs have more secrecy than the other people.ie heterosexuals, MSMs like mostly to hide not to be known by any one, especially the one who play receptive role among the two he most of the time has no problem with being known but the top most of the time he hides so that people may not know him.so there is a challenge, which I think if we use the bottom although they sometime exchange roles most work like that but most you will find the bottom is supposed to bring his sexual partner on this PNS services and it could be good if we can start looking for them and see where to find them because we can't discriminate them, for somebody like me ,if I know that there is one or two who are MSM and if I know there are PNS services required I can't say that I will leave them just like that without explaining them about PNS and there is a very big importance that they require such services but the challenge that a man will never accept such services just easily like that because they like hiding a lot.so my opinion is they should also get the services.

**[INTERVIEWER]**: so, it can be successful although there will be challenges.

**[PARTICIPANT]:** There should be added more ideas first.

**[INTERVIEWER]:** and if it is done do you see the MSM benefiting from the service, any positive outcome that will come from PNS in case it is made happen?

**[PARTICIPANT]**: There are a lot of benefits because education does not end, it's a lot I have leant since I came to [RESEARCH_INSTITUTION] which I didn't know. And they will also benefit a lot from PNS which they didn't know, sometimes other people argue a lot or afraid because they will not have undergone the services but the services when they start, through testing and gone through PNS he sometimes feels like opening up which at first, we could not be free.so there are a lot of benefits where they will get.

**[INTERVIEWER]:** For yourself which benefit have you gotten through PNS?

**[PARTICIPANT]:** the first thing I had, seeing me that am a living dead, that came out completely, second the body I had at first and now it has changed, I remember the first time I came you were looking for veins which was very difficult to get them because I was very thin and weak but now am okay, third is that am now free to talk to people ,I don't know why but I just found myself to have released everything but am in control, so you see things are improving because most of the time I could complain about backbone because I have slept and I can't do anything.by interacting with my fellow friends when telling stories ,I was surprised to see my friends life if very good but I get sick always but for now I have shared a lot you see then I can tell about my future because there is a nice place am going , that time I couldn't talk. I just knew that I could die anytime so it gave me hope, so those are the benefits I got from since I started ART.

**[INTERVIEWER]:** so, the dugs have really helped you?

**[PARTICIPANT]:** yes

**[INTERVIEWER]:** can you tell me the methods we said we can use for PNS or to inform the sexual partners. We say there are these people who are HIV infected just like you, where you are our study participant and we have that peer mobiliser and we have the counselor.so all these people can together to make PNS success or inform the sexual partners so that they can test and know their HIV status. Because they all play different roles on PNS.so we are saying the first one the one we did for you the counselor contacting your sexual partners and inviting them to come and have the HIV testing.so if the method is successful he applies a lot of confidentiality for the infected. The second method is the peer mobiliser to give the index client the self-test kit to go and give it to his sexual partner at home. Many prefer taking the self-test kit to the sexual partner to give the sexual partner to test where he will gain strength and morale to know more that's why he will come for confirmatory blood test. The third method is when the peer mobiliser will go to where the index partner goes either to sit with his friends (hot spot) and may the client he will be looking for likes to hang there. If he goes to such hot spot he gives out the OST to everybody emphasizes the OST he is giving free of charge where in the chemist its being sold and it's important to confirm with the blood test in the hospital although the mobiliser will know the one he is targeting for him to not realize we were looking for him, if he does that he prevents if the index participant of disclosing. The fourth method is peer mobiliser to assist the index client invite his sexual partners to come and do couple testing or assist the index client to open up to his sexual partners to come for HIV testing together. The last method is the index client like you to give your sexual partners the OST for testing so that he can test for HIV virus. So, of all these methods of PNS we have discussed the one we have used to your situation is the counselor who contacted your sexual partner's so we say that method was the one you thought it was nice to you according to you or you think there is another one you feel it be used so that we can get the second partner whom we have not yet contacted him?

**[PARTICIPANT]:** to me I think the one I recommended is the best way you can use it, because for me to get them where they are living for now it will be difficult.

**[INTERVIEWER]:** oh, it's okay. And it's after how long you were explained about PNS since you knew your status?

**[PARTICIPANT]**: just one month.

[INTERVIEWER]: is it true

**[PARTICIPANT]:** we discussed the first day we met and then this is the second time we are talking about it again.

**[INTERVIEWER]**? So, on the first day you knew your HIV status is the very day PNS was introduced to you?

**[PARTICIPANT]**: yes

**[INTERVIEWER]:** and do you think on the first day it was the best time to discuss PNS or you think you could be given more time before talking about PNS according to you.

**[PARTICIPANT]:** with me I was very comfortable because was very ready for any programme because I was very sure that because it has gotten me, they can get another person so it was better we discuss everything at ago so that even others can get help.

**[INTERVIEWER]**: oh, that's okay.

There is the method we use to contact the sexual partners through phone calls, we invite them for HIV testing, maybe the words we are using for example I tell them that my name is so and so and am calling you from a certain organization and we are calling because it's important for you to come and test and know your status because you are at risk of contracting, or I tell him that we are doing research here where we invite people for testing and when people find they are HIV infected it's important to look for their sexual partners to come and test their HIV status and if possible this number is one of the numbers I have been given for those people we are looking to come and know their status. These are the words we can use to call your sexual partners; how do you see about these wordings or you have other words you can give us so us to use them which will enable them to come?

**[PARTICIPANT]:** mmhhh the question is very challenging because their people who don't want to hear the word HIV testing.

**[INTERVIEWER]:** yes

[PARTICIPANT]: the moment you mention the word they feel upset by the word testing, but for this one who have travelled he has no any problem. The other one I don't know his respond will be like what but the other one has no problem depending what you will tell her she is not arrogant and she will just answer you if she will be free she can take her time and come in fact she likes most of the time to know her HIV status. If there will be any issues what's she wants her things to run smoothly. Most of the time people have issues about HIV testing.

**[INTERVIEWER]:** so, we try to remove the aspect of HIV testing?

**[PARTICIPANT]**: no, for the other one she has no problem.

**[INTERVIEWER]**: so, she has no problem, so we will continue looking for her

**[PARTICIPANT]**: yes, just continue looking for her, also I will try to keep her on phone so that I can try to look for him.

**[INTERVIEWER]:** we are heading towards the end of the interview; may you have any recommendation about to improve and successfully do PNS? Especially for MSM.

**[PARTICIPANT]**: what I can talk about is the MSM, those are the people to be given first priority about PNS, because the many risks we have ,so those are the ones supposed to be in the program and we find more ways that we can educate the MSM bout PNS ,being interviewed, to open up, if you keep confidentiality then your information will also store, so don't think there any barrier for opening up, so PNS should reach to them like what I did , I was very open and explained to you, so its them that they are free to come out because we will make this PNS is successful which will benefit a lot of people.

**[INTERVIEWER]:** yes, it's very important.

Thanks a lot for your time, I don't think if you have any other issues you would want to add before we complete?

**[PARTICIPANT]:** My question, is about the ART drugs, the day I was given, I was told they will have side effects, like dizziness and I asked why ,I was told it's because they will be fighting with the viruses, so I don't know if it's a must to experience the side effects or you can take and nothing will happen to you and do you know as to why am asking like that, it's because didn't experience such so does it mean that the drugs did not work in my body? I thought it was a must they could fight.so does it work either way or it's a must they fight so that they can give you the dizziness?

**[INTERVIEWER]:** no, it's not a must you experience the side effects because we have different body reactions and they had to explain because in case they could react with your body and they didn't tell you could be surprised, so it was very important to explain it to you.

**[PARTICIPANT]**: ooh

[INTERVIEWER]: what you were told if something could happen to you they advised to go back to the clinic but if nothing happen its shows that you have a good body.

**[PARTICIPANT]:** that's was the only question I had.

**[INTERVIEWER]:** thanks for your time, thanks so much and this makes the end of our interview plus you participating in this study. Services will still be provided to you but now thanks for you participating.

**[PARTICIPANT]:** Thank you too.
