# Supplementary material for: Peer-assisted HIV partner notification services to strengthen index partner testing for newly diagnosed men who have sex with men in coastal Kenya
Source: PLoS One. 2025 Oct 7;20(10):e0333707. doi: 10.1371/journal.pone.0333707 (PMC12503256; doi:10.1371/journal.pone.0333707)
Supplement: S3 Appendix — (ZIP) [file pone.0333707.s003.zip › Deidentified IDI Transcript_1339.docx]

**Participant characteristics:**

Age: 20-24

Sexuality: Gay

Education level: Vocation

Days between enrollment and IDI: 38 days

Mobilization strategy: OST

Final PNS Strategy: HCP/PM

**Partners identified: 9**

**[INTERVIEWER]:** welcome to this interview which is taking place today on [DATE]. Thank you very much for coming for the interview and agreeing to participate in this interview. As I had mentioned earlier, we will tape record this interview so that we will be able to capture every opinion, thoughts and ideas when we write a report. On to my first question how are you feeling?

**[PARTICIPANT]:** Thank you, I am feeling good and concerning the ARVs I'm now used to taking them and isn't a big deal anymore.

**[INTERVIEWER]:** Is there a time that you got a hard time taking the ARVs?

**[PARTICIPANT]:** The first few days it was challenging, I used to feel dizzy sometimes, but after a week or so I started feeling like it's a normal thing.

**[INTERVIEWER]:** What are your feelings and psychological status since you found out that you were HIV positive?

**[PARTICIPANT]:** In short I am happy to have known that I am HIV positive on time because I start taking the ARVs and prevent myself from infecting other people unknowingly.

**[INTERVIEWER]:** Okay, what about emotionally and your thoughts? Are you still feeling the way you were after knowing that you were HIV positive?

**[PARTICIPANT]:** To be honest when found out that I was HIV positive I was shocked because I wasn't expecting the results I got but after I was counselled I accepted that I'm HIV positive and I'm living a normal life.

**[INTERVIEWER]:** Okay so counselling really helped you and you are better now and that got some side effects in the first seven days but now you are okay. Do you remember the first time you found out that you were HIV positive?

**[PARTICIPANT]:** The very first time I took a HIV test was in 2014, but finding out that I was HIV positive I think it was two months ago.

**[INTERVIEWER]:** What reason made you take the HIV test those two months ago?

**[PARTICIPANT]:** I had a friend who told me about [RESEARCH_INSTITUTION] and when we got time we came because I wanted to know my HIV status, so took the test and found that I was HIV positive.

**[INTERVIEWER]:** What reason made you decide to listen and follow what the mobilizer told you till you decided to take the HIV test? Because sometimes the mobilizer can approach a person and that person can chose to come or not.

**[PARTICIPANT]:** Thanks, what made me decide to take the test was that along time had passed since I took the HIV test and what he told me made me have the need to know my HIV status.

**[INTERVIEWER]:** Did the risk behaviours you were involved with play a part in your decision making of getting the HIV test? For example having unprotected sex or having several sexual partners.

**[PARTICIPANT]:** Most of the time I had sex with my partners not all of them accepted to use condoms and I don't know why I didn't have the thought of getting to know my HIV status but I'm grateful to God for bringing the mobilizer to me on time.

**[INTERVIEWER]:** Okay so your reason for accepting to take the test is because the mobilizer approached and you just decided to take the test.

**[PARTICIPANT]:** Yes that's it.

**[INTERVIEWER]:** Okay. How did the mobilizer approach you?

**[PARTICIPANT]:** He's my friend I didn't even know that he is a mobilizer, I just found him telling me about [RESEARCH_INSTITUTION] and all that and I got interested and wanted to hear more.

**[INTERVIEWER]:** Okay and where did the mobilizer approach you where did you meet?

Because he's a friend he came to my place.

**[INTERVIEWER]:** When he came over and he started talking about taking the HIV test, did you guys speak of other things like risk behaviours, self-testing kit or acute HIV infection?

**[PARTICIPANT]:** Yes we did talk about the risks and how risky it is to be infected and how I prevent myself from the risks of being infected.

**[INTERVIEWER]:** What are the risk behaviours that the mobilizer told you about?

**[PARTICIPANT]:** The first one was unprotected sex and that's the worst risk one can take.

**[INTERVIEWER]:** Any other risk behaviour?

**[PARTICIPANT]:** No I don't remember because we talked about a lot of things.

**[INTERVIEWER]:** That's fine, what do you remember about the oral self-test in your conversation with the mobilizer?

**[PARTICIPANT]:** The mobilizer told me that it's an equipment that a person can use to get to know their HIV status through rubbing it in the gums and it's easy to use and one can use it even at home. I loved the equipment because it gives a person some privacy.

**[INTERVIEWER]:** What do you remember about acute HIV infection?

**[PARTICIPANT]:** The mobilizer told me that if you find out that you are infected in may be the first three days I think there's some medication you'll be given and the virus will be cured.

**[INTERVIEWER]:** Is that all the mobilizer talked to you about acute HIV infection?

**[PARTICIPANT]:** No that's not everything I'm just having trouble remembering most of the things.

**[INTERVIEWER]:** That's okay, it has been a long time since you came here and we are human beings we have a lot going on, was it easy for you to comprehend all that the mobilizer was telling you?

**[PARTICIPANT]:** No it wasn't easy because I only had shallow knowledge about HIV, some the information was really new to me.

**[INTERVIEWER]:** Did the mobilizer share any materials with you? Some kind of pamphlets?

**[PARTICIPANT]:** No I wasn't given any material, we just talked.

**[INTERVIEWER]:** Okay, how do you think we can motivate the GBT to be frequently coming for HIV testing?

**[PARTICIPANT]:** It's a very important question, because a lot of people, especially gays some of them have accepted that they are gays but going to the health facilities to get to know their HIV status is hard what I'm suggesting is that in the forums and gatherings we should be told more about HIV, the importance of frequent HIV testing and dangers of not knowing your HIV status in time. If people get to know more about the self-testing kit this will help people feel the ease of getting to know their HIV status and it gives them the privacy they need.

**[INTERVIEWER]:** That's great, you mentioned that you used the OST, what was your experience?

**[PARTICIPANT]:** When I got the equipment I was surprised because I had not seen such a thing, but once I opened it and read the instructions and the mobilizer had already also told me what to do. It was easy to use.

**[INTERVIEWER]:** So it was challenging to you, where did you conduct the test?

**[PARTICIPANT]:** I was here at [RESEARCH_INSTITUTION], in a room that no one was in, just me.

**[INTERVIEWER]:** Did you start taking your ARVs the exact same day you found out that you were HIV positive?

**[PARTICIPANT]:** No, I just direct back home give myself time to think about what I am going to do, then after two days is when I came back and started taking the ARVs.

**[INTERVIEWER]:** Why did it take you two days before you decided to come back for the ARVs?

**[PARTICIPANT]:** I was in shock and I had not accepted myself but when I went home I digested the news and since I love my life so I came back.

**[INTERVIEWER]:** Yeah, everyone loves their lives, and when you came back after the two days and you were told to start taking the ARVs what was going on in your mind about starting the treatment?

**[PARTICIPANT]:** I asked myself where will I be keeping these ARVs because at my place my friend's come over and if they see them they'll start to stigmatize me I had a lot in mind but I am grateful.

**[INTERVIEWER]:** What are you grateful for?

­**[PARTICIPANT]:** (*chuckling*) Starting the treatment.

**[INTERVIEWER]:** How did you address your fears? Like making sure your friends don't get to see the ARVs?

**[PARTICIPANT]:** I was just very careful and kept the ARs far from where people are.

**[INTERVIEWER]:** And where is that?

**[PARTICIPANT]:** I put them in paper bag and then put them somewhere very private.

**[INTERVIEWER]:** Can you tell me more if you are comfortable about the the hiding place?

­**[PARTICIPANT]:** (*chuckling*) I put it under the bed.

**[INTERVIEWER]:** Okay, what can you tell me about your post counselling?

**[PARTICIPANT]:** Before I took the second test after using the OST and the results were HIV negative, I don't know why, the counsellor talked to me first and told me if I turn out to be HIV positive I should accept and start the treatment and if I'll turn out to be HIV negative I should take PrEP and keep myself safe.

**[INTERVIEWER]:** Okay, what about after the two day?

**[PARTICIPANT]:** When I came back to start the treatment, I was told to be very careful and specific when taking the ARVs, if I have decided to be taking the ARVs at 9 pm I should do that every day and if I delay I shouldn't delay for more than 12 hours.

**[INTERVIEWER]:** Okay, and were you contented with the counselling you got? For example, was there something you think was missing or was there more you think you could have been told

**[PARTICIPANT]:** I was satisfied with the counselling I was recived a lot that was beneficial to me.

**[INTERVIEWER]:** How was PNS introduced to you?

**[PARTICIPANT]:** I liked the PNS it is a good thing, especially using the healthcare service provider because facing someone on my own could have been really hard and could have been violent.

**[INTERVIEWER]:** What are the benefits of PNS?

**[PARTICIPANT]:** It's very beneficial because if I was contacted and notified by my partners in time I think I wouldn't have been HIV positive but on my side I think PNS is an important thing.

**[INTERVIEWER]:** Okay, how many strategies of PNS did you discussed, which one did you use?

**[PARTICIPANT]:** I think they were four, the first one was using the mobilizer, the healthcare service provider, approaching them myself but the last one I can't remember.

**[INTERVIEWER]:** Okay and what method did you chose?

**[PARTICIPANT]:** The appropriate method for me was through the healthcare service provider to contact my partners and I chose this because I thought that getting information about something from a professional in that field is taken to be serious.

**[INTERVIEWER]:** What is your opinion about the method you chose right now? For example, If you were given a chance to choose again will you chose the same method or will you change your mind?

**[PARTICIPANT]:** I think I can try another method of approaching my partners myself because I feel I'm composed and courageous enough to face them now.

**[INTERVIEWER]:** Do you know if your partners were contacted? I still don't know but I think they were contacted, and may be they didn't show up.

**[INTERVIEWER]:** Do you remember how many partners did you mention?

**[PARTICIPANT]:** I think 6 or 5 partners.

**[INTERVIEWER]:** You mentioned 10.

**[PARTICIPANT]:** Yeah I was just mentioning the ones from here didn't include the ones from [CITY_D].

**[INTERVIEWER]:** What do you think was their reaction when they were contacted? Each one of them.

**[PARTICIPANT]:** Since they are different people with different characters, I think some were just calm and others wanted to come.

**[INTERVIEWER]:** Did the PNS affect your relationship with your partner?

**[PARTICIPANT]:** No, everything is okay.

**[INTERVIEWER]:** How was your experience with being informed about PNS for the first time?

**[PARTICIPANT]:** The first time I didn't want to comply because some people don't like their numbers being given to strangers and it could have been harmful to me. I just trusted the healthcare service provider and believed that they are professionals and they can handle the situation well.

**[INTERVIEWER]:** What if I told you that your partners were contacted but they haven't come here to be tested or they promised to go get tested somewhere else but they have not given me their results, what do you think should be done to help them?

**[PARTICIPANT]:** I think I can approach them myself and tell them the importance of knowing their HIV status.

**[INTERVIEWER]:** Okay and how will you approach them?

**[PARTICIPANT]:** I can't disclose to them that am HIV positive, I'll just do the mobilizer way.

**[INTERVIEWER]:** Have disclosed your HIV status to anyone?

**[PARTICIPANT]:** No, I haven't told anyone.

**[INTERVIEWER]:** If you were to tell someone who would you tell?

**[PARTICIPANT]:** My mother, but not soon.

**[INTERVIEWER]:** Why not soon?

**[PARTICIPANT]:** I've just started my treatment if I tell her now she'll be very shocked and might cause her some health issues but if I wait for like 5 years or so and tell her, she will be shocked but not as much because I would have lived for 5 years since I found out that I am HIV positive and that will encourage to take things light.

**[INTERVIEWER]:** What will be your reason for telling your mother and not a friend or boyfriend?

**[PARTICIPANT]:** I'm very close to my mother and I find it safer telling her but with friends they will start spreading the news.

**[INTERVIEWER]:** How do you think disclosing your HIV status to your mum will affect your relationship with her?

**[PARTICIPANT]:** I don't think if it will our relationship what I fear is that she might not be at peace.

**[INTERVIEWER]:** Okay, you already told me that you have not been exposed to social harm and that none of your partners has suspected that you are the one who gave out their phone numbers? Do you think that there can be any harm with the PNS?

**[PARTICIPANT]:** Yes, am glad it didn't happen to me, but risks are there for example it can to violence.

**[INTERVIEWER]:** Any other harm?

**[PARTICIPANT]:** It can affect the relationship and if someone is very keen they might have a feeling that I'm infected and that's not good at all.

**[INTERVIEWER]:** How was it you talking to me about your sex partners in the first interview?

**[PARTICIPANT]:** It wasn't easy because remembering the partners I've been with for the last 12 months wasn't easy.

**[INTERVIEWER]:** Why was it hard?

**[PARTICIPANT]:** I move around a lot, I travel a lot and I've been with a lot of people in those different places so knowing the number is hard and 12 months is a long time.

**[INTERVIEWER]:** Are there any partners that we didn't speak about in our first interview?

**[PARTICIPANT]:** Yes there are some.

**[INTERVIEWER]:** How many?

**[PARTICIPANT]:** Roughly 10, they cannot be more than 10.

**[INTERVIEWER]:** Do you have their contacts?

**[PARTICIPANT]:** Some I do but some I think I lost them but I can try to get their contacts.

**[INTERVIEWER]:** Okay, what was the reason for not mentioning the ones you just mentioned?

**[PARTICIPANT]:** I have two sim cards and I left one at home last time so the ones I had their contacts at hand are the ones I mentioned.

**[INTERVIEWER]:** what do you think can facilitate uptake of partner notification services to GBT and have less challenges?

**[PARTICIPANT]:** Sometimes the fear of the partner wondering where the healthcare service provider got his number from.

**[INTERVIEWER]:** What else?

**[PARTICIPANT]:** The partner may be aware of their HIV status and this can discourage them to come for another HIV test.

**[INTERVIEWER]:** How can we improve PNS in the GBT community?

**[PARTICIPANT]:** The OST will be very helpful cause if you give someone that kit it will be easy for them to take the test at the time they feel comfortable and after testing if they find out that they are HIV positive they will obviously go to a clinic to get treatment.

**[INTERVIEWER]:** Who will take the OST to the partners?

**[PARTICIPANT]:** The mobilizer.

**[INTERVIEWER]:** How best do you think the mobilizer can best approach them?

**[PARTICIPANT]:** I can also do the distributing. There are some friend of mine who know about the OST but they have no access to it, some of them are friend and some of them are partners.

**[INTERVIEWER]:** Out of all the PNS methods which one do you think is best?

**[PARTICIPANT]:** Id approach them myself and also use the healthcare service provider to contact them our efforts together might be stronger and work faster.

**[INTERVIEWER]:** So you don't find one specific method that is best but you feel like two methods combined can be better.

**[PARTICIPANT]:** Yes.

**[INTERVIEWER]:** And what PNS methods do you think can bring out the best results when combined?

**[PARTICIPANT]:** Those two I had mentioned earlier are the only ones can work best. If they are used at once they'll have a better impact.

**[INTERVIEWER]:** Okay what if the healthcare service provider contacts your partner and tells them that they are at risk of getting infected with HIV because of having sex with their recent partner and that he should come to [RESEARCH_INSTITUTION] for counselling and getting tested while at the same time you are also telling him that you two should go get tested for HIV at [RESEARCH_INSTITUTION], don't you think he will suspect that you are the one who gave out his number?

**[PARTICIPANT]:** I didn't not look at it at that angle, (*chuckling*) I think I can withdraw my statement and say that one method should be used first if it fails now the one can be used.

**[INTERVIEWER]:** Okay so for instance you have given the phone number, I've called your partner but they are not responding the way we expected and you suggest that we should let you approach him, after how long do you think we should give you for you to approach him?

**[PARTICIPANT]:** We can wait and give it some time like some few weeks or a month so that he can forget and not connect that am liaising with the healthcare service provider from [RESEARCH_INSTITUTION].

**[INTERVIEWER]:** Okay thanks you very much, do you have any other method that you think can help contact the partners apart from the mentioned PNS methods?

**[PARTICIPANT]:** Most of the time we get people who wants to have sex with me without a condom it's the right time to ask this person to take a HIV test because we are not supposed to have sex with people who we don't know their HIV status. We can use the OST at that time.

**[INTERVIEWER]:** Okay , but what am asking is after knowing the client is HIV positive is there another way we can get to contact the partners?

**[PARTICIPANT]:** Then there's none.

**[INTERVIEWER]:** Do you think that the time you were introduced to PNS was an appropriate time?

**[PARTICIPANT]:** I think it was the right time because the earlier the better if I was also contacted earlier I might have been a different case.

**[INTERVIEWER]:** Do you think it's appropriate to talk about PNS to a client on the very day that they found out that they are HIV positive?

**[PARTICIPANT]:** Yes it is.

**[INTERVIEWER]:** If you were a healthcare service provider and was given a phone number to contact the partner how would you do it?

*(Beginning of the role play between a healthcare service provider and a client)*

**[PARTICIPANT]:** Hello

**[INTERVIEWER]:** Hello

**[PARTICIPANT]:** I am a counsellor from [RESEARCH_INSTITUTION] and I'm asking if you would like to know you HIV status

**[INTERVIEWER]:** Why should I do that and I don't even know who you are.

**[PARTICIPANT]:** It's important and good to know your HIV status.

**[INTERVIEWER]:** I don't want to and I'm not ready because I took the test less than 3 months ago.

**[PARTICIPANT]:** it's okay but I would like you to come after the 3 months are over so as to know your HIV status.

*(End of role play)*

**[INTERVIEWER]:** (Laughs) that's nice so in the role play you didn't tell the partner that they are at risk of being infected and they've had sex recently with someone that is infected because you feel like that is not an appropriate way to approach a partner

**[PARTICIPANT]:** Yeah. I'd just ask if they can come to and take the test, because telling them all that over the phone they might start getting violent and start asking a lot of questions.

**[INTERVIEWER]:** But that's how we do it, you think it's not a good way?

**[PARTICIPANT]:** It's also okay but according to me my way can also work.

**[INTERVIEWER]:** Put yourself in the partners shoes and you are told all that, how will you take it?

**[PARTICIPANT]:** I'll be very shocked and I might have no idea of the client and will have a lot of questions.

**[INTERVIEWER]:** And would have come?

**[PARTICIPANT]:** Yes, because I love my life.

**[INTERVIEWER]:** Okay and your way of approaching a partner just telling someone to go get tested and not telling them the importance of knowing ones HIV status would you come?

**[PARTICIPANT]:** It's going to be hard because it's a stranger who called me.

**[INTERVIEWER]:** How do you think we can build trust and confidentiality with PNS?

**[PARTICIPANT]:** By just telling the people you call that you just got random contacts and convincing people to come and get tested because it's important to know their HIV status.

**[INTERVIEWER]:** Okay, do you have any recommendations that can improve the PNS? **[PARTICIPANT]:** No I don't but just keep doing it because it helps a lot of people even though it's challenging.

**[INTERVIEWER]:** Is there anything else you would like us to discuss about PNS and GBT?

**[PARTICIPANT]:** Yes there's a lot to discuss, we are minorities and most people don't like us, I would just want the GBT to come together and get to know each other and let the rest know that we are human beings and that we also have rights. So when it comes to PNS we will easily be able to identify, remember and approach our partners because we will be familiar with each other.

**[INTERVIEWER]:** Is there anything else you would like to add?

**[PARTICIPANT]:** (***chuckling*) No, I have nothing else to add.

**[INTERVIEWER]:** thank you very much for coming and dedicating your time for sharing your views and experiences. I truly appreciate. We have come to the end of our interview.
