# Supplementary material for: Peer-assisted HIV partner notification services to strengthen index partner testing for newly diagnosed men who have sex with men in coastal Kenya
Source: PLoS One. 2025 Oct 7;20(10):e0333707. doi: 10.1371/journal.pone.0333707 (PMC12503256; doi:10.1371/journal.pone.0333707)
Supplement: S3 Appendix — (ZIP) [file pone.0333707.s003.zip › Deidentified IDI Transcript_1343.docx]

**Participant characteristics:**

Age: 25-29

Sexuality: Gay

Education level: Primary

Days between enrollment and IDI: 34 days

Mobilization strategy: OST

Final PNS Strategy: HCP/PM

**Partners identified: 2**

**[INTERVIEWER]:** Thank you very much for coming for the interview and agreeing to participate in this interview. As I had mentioned earlier, we will tape record this interview so that we will be able to capture every opinion, thoughts and ideas when we write a report. Is that okay?

**[PARTICIPANT]:** Yes it is.

**[INTERVIEWER]:** How are you feeling right now?

**[PARTICIPANT]:** I'm doing fine, I feel healthy and I have no complications or problems like before.

**[INTERVIEWER]:** You mean to say that there's a time that you had health issues or problems?

**[PARTICIPANT]:** Yes, when I first found out about my HIV status I became weak.

**[INTERVIEWER]:** How did finding out that you were HIV positive affect you?

**[PARTICIPANT]:** I felt like it's the end of me, I felt worthless and decided to isolate myself from the rest but right now I'm better and I don't see any difference between other people and I.

**[INTERVIEWER]:** What is it that helped you become who you are right now?

**[PARTICIPANT]:** Counselling, mostly from the clinic and other people close to me.

**[INTERVIEWER]:** What challenges and worries did you face when you found out that you were HIV positive?

**[PARTICIPANT]:** I felt very worthless and even thought of committing suicide but I'm really doing fine right now.

**[INTERVIEWER]:** I'm glad that you are now feeling great you have no challenges or problems. How are the ARVs taking you?

**[PARTICIPANT]:** At first whenever I took them I used to feel sickly and weak and cold but right now I don't feel that anymore.

**[INTERVIEWER]:** How long did the side effects last?

**[PARTICIPANT]:** Four to five days.

**[INTERVIEWER]:** When did you learn that you are HIV positive?

**[PARTICIPANT]:** It was on [DATE].

**[INTERVIEWER]:** What was the reason that made you decide to get tested for HIV?

**[PARTICIPANT]:** I just thought it was important for me to know my HIV status. I didn't even suspect that I'm going to be found HIV positive, I thought I was just okay.

**[INTERVIEWER]:** I am aware that you came here through the mobilizer, how did the mobilizer approach you?

**[PARTICIPANT]:** He told me the importance of knowing your HIV status, and if you know your HIV status you'll also get to know your partners HIV status at the same time.

**[INTERVIEWER]:** Did he tell you about any risk behaviours?

**[PARTICIPANT]:** He told me about having sex with without knowing your HIV status or your partners HIV status and having unprotected sex, also having anal sex is the most risky way of getting infected. Also group sex.

**[INTERVIEWER]:** Did he talk to you about acute HIV infection?

**[PARTICIPANT]:** I don't remember that.

**[INTERVIEWER]:** Did you know about these risk behaviours before the mobilizer informed you about them?

**[PARTICIPANT]:** I didn't know at all.

**[INTERVIEWER]:** Did the mobilizer give you any papers that contained some information when you came in?

**[PARTICIPANT]:** Yes he did.

**[INTERVIEWER]:** And what kind of information did they have?

**[PARTICIPANT]:** The papers were explaining on the ways that HIV can easily be transmitted from one person to another, and that HIV can affect anyone regardless who you are having sex with whether its same sex or man and woman sex they can all be infected by HIV.

**[INTERVIEWER]:** what did you think about the papers that had such information, was it easy to comprehend?

**[PARTICIPANT]:** They were easy to comprehend and the information was clear and straight to the point.

**[INTERVIEWER]:** What are some of the good things that the mobilizer did?

**[PARTICIPANT]:** He advised me to get tested and know my HIV status and also advised me to accept my HIV results as they are no matter the outcome.

**[INTERVIEWER]:** What are some of the things do you think need to be improved about your mobilizer?

**[PARTICIPANT]:** On my side I was satisfied with the services.

**[INTERVIEWER]:** In your own opinion what are some of the ways do you think we can convince the GBT to be taking the HIV test?

**[PARTICIPANT]:** I know so many who don't like taking the HIV test, but once they get those papers with the information on HIV I was given they will read and get to understand the importance of knowing their HIV status.

**[INTERVIEWER]:** What is your experience while using the self-test kit?

**[PARTICIPANT]:** It was easy and not painful like the finger prick.

**[INTERVIEWER]:** When did you start using the ARVs?

**[PARTICIPANT]:** On that same day that I found out that I was HIV positive.

**[INTERVIEWER]:** What comments do you have about your first post counselling?

**[PARTICIPANT]:** It motivated me, I saw that I can still live my life even when I'm HIV positive.

**[INTERVIEWER]:** How was PNS introduced to you?

**[PARTICIPANT]:** I was told about it on that same day I found out that I was HIV positive. I was asked how many partners I had, if they are nearby or are far, how long have I been with them sexually. They also asked me how they can be contacted and what method am I comfortable with and I decided to give the healthcare provider my partners contacts so they can communicate with them.

**[INTERVIEWER]:** Why did you chose the healthcare provider method?

**[PARTICIPANT]:** The self-testing method could have been a challenge because I was to be given just one self-test kit and my partner would ask me where was mine and might decide to decline. And about the other method of disclosing my HIV status to my partner things might gotten violent and I was avoiding that.

**[INTERVIEWER]:** What are your thoughts about the method you chose?

**[PARTICIPANT]:** It also has its own challenges on the partner and the healthcare provider and not the client. It's the best way because they do not disclose the client in any way there's some privacy.

**[INTERVIEWER]:** How many partners did you mentioned?

**[PARTICIPANT]:** Three.

**[INTERVIEWER]:** Are there any more partners that you perhaps forgot to mention during the initial interview?

**[PARTICIPANT]:** No, it was only the 3

**[INTERVIEWER]:** Were they all contacted and tested for HIV?

**[PARTICIPANT]:** Two were contacted and took the test but one has not yet been contacted.

**[INTERVIEWER]:** How did you get such information?

**[PARTICIPANT]:** The counsellor asked me of how best we can engage the one partner that haven't reported, definitely I knew the two came and the one I was asked my suggestion of engaging him is the one who haven't come.

**[INTERVIEWER]:** How do you think your partners reacted after being contacted?

**[PARTICIPANT]:** I'm sure it shocked them and questioned who gave their number.

**[INTERVIEWER]:** Have you told anyone about your HIV status?

**[PARTICIPANT]:** Yes, five people know about my HIV status.

**[INTERVIEWER]:** What is your relationship with those five people?

**[PARTICIPANT]:** The first one is my parent, the second one is my cousin, the third one is just a friend, and the Fourth one is a church friend and lastly my pastor.

**[INTERVIEWER]:** Did you tell them more about your sexual preference or you just told them about your HIV status?

**[PARTICIPANT]:** It's hard to come out as gay and I just told them that I decided to get be tested and found out that I'm HIV positive.

**[INTERVIEWER]:** Why did you decide to disclose your HIV status to your mum and not your brother or sister?

**[PARTICIPANT]:** My mother is wiser and can advise me better, she was hurt but she just accepted it.

**[INTERVIEWER]:** Do you think that the people you told have not told anyone else about your HIV status?

**[PARTICIPANT]:** Yes they have not told anyone else because I haven't heard anyone talking about it.

**[INTERVIEWER]:** Among the five people you told, has the disclosure affected any of your relationship?

**[PARTICIPANT]:** No it hasn't.

**[PARTICIPANT]:** How do you think your disclosure about your HIV status has affected the people you've told?

**[INTERVIEWER]:** All of them were hurt and some even started thinking that my life is over but I knew differently because I was using the ARVs and everything is good.

**[INTERVIEWER]:** Have you experienced any safety issue with the PNS?

**[PARTICIPANT]:** No, I haven't. The method I chose was discreet and safe.

**[INTERVIEWER]:** What do you think we can do to improve the PNS?

**[PARTICIPANT]:** Nothing because the client name is not mention in the conversation it's just perfect the way it is.

**[INTERVIEWER]:** What if your partner knows that you are the only one he has been with sexually for the past six months, don't you think he can still tell that you are responsible for the call without the healthcare provider mentioning you?

**[PARTICIPANT]:** He will know.

**[INTERVIEWER]:** Then what do you think might happen?

**[PARTICIPANT]:** He will hate me, and get angry and fight me or even commit suicide.

**[INTERVIEWER]:** How was your experience with being introduced to PNS on the same day that you found out that you were positive?

**[PARTICIPANT]:** It was hard, because being gay is a huge secret but since I also wanted them to know their HIV status early enough I had to come out so that they can be helped too.

**[INTERVIEWER]:** Was it easy for you to discuss the partners you've been in the past 12 months?

**[PARTICIPANT]:** It was hard, because it's hard to remember because 12 months is a long period and people can have very many partners in that period.

**[INTERVIEWER]:** Do you think it is important to notify and contact partners of a GBT client? **[PARTICIPANT]:** Yes it is important, because the partner might have other partners too this will help prevent the spread of HI V.

**[INTERVIEWER]:** What challenges are there in PNS?

**[PARTICIPANT]:** Good communication between the partners and the healthcare service providers. Some partners will be rude and not respond accordingly, they might even threaten or hang up the phone on the healthcare service provider.

**[INTERVIEWER]:** What about on the healthcare service provider's side?

**[PARTICIPANT]:** On the healthcare service providers side he/she should also be understanding and patient with the rude partners. They should understand that it is not easy accepting a stranger calling you and telling you that you are at risk of being infected with HIV.

(*The [INTERVIEWER] asked the [PARTICIPANT] to play the role of the healthcare service provider and show how he would have contacted and notify one of the client's partners)*

**[PARTICIPANT]:** Hello

**[INTERVIEWER]:** Hello

**[PARTICIPANT]:** I'd start by asking you, about the number of partners you have.

**[INTERVIEWER]:** Why would I tell you about my partners when I don't even know who you are or where you got my phone number from?

**[PARTICIPANT]:** (*Chuckles*) that's hard now.

**[INTERVIEWER]:** I am your client now talk to me.

**[PARTICIPANT]:** Okay, hello.

**[INTERVIEWER]:** Hello.

**[PARTICIPANT]:** I am a healthcare service provider from [RESEARCH_INSTITUTION] am I speaking to R.

**[INTERVIEWER]:** Yes, R speaking.

**[PARTICIPANT]:** I got your number from somewhere and I'm a counsellor of the GBT and I want to know if you are part of the GBT group?

**[INTERVIEWER]:** I am not part of them.

**[PARTICIPANT]:** Okay, whether you are or not, I just want to ask you if you know your HIV status?

**[INTERVIEWER]:** No I don't know, it has been long since I took a HIV test.

**[PARTICIPANT]:** Would you like to know your HIV status?

**[INTERVIEWER]:** What do you want from me?

**[PARTICIPANT]:** I want nothing from you, I want you to be aware of your HIV status it's not advisable to stay for long without taking the HIV test.

**[INTERVIEWER]:** Okay I will do that.

**[PARTICIPANT]:** Is there by chance that you can come to [RESEARCH_INSTITUTION] to our clinic so that you can get more services like counselling?

**[INTERVIEWER]:** Yes I can.

**[PARTICIPANT]:** When can you make it?

**[INTERVIEWER]:** Today is Tuesday so I'll come on Thursday.

**[PARTICIPANT]:** What time?

**[INTERVIEWER]:** You tell me what time your clinic is open.

**[PARTICIPANT]:** it's open from 8 a.m. to 5 p.m.

**[INTERVIEWER]:** Okay ill come by at 10 a.m.

**[PARTICIPANT]:** Okay, when you arrive give me a call so we can met up.

**[INTERVIEWER]:** Okay I will.

*(End of the role play)*

**[INTERVIEWER]:** Great, You just talked about when was the last time I took the test. Why didn't you tell me that one of my partners that I have had a sexual encounter with recently was found to be HIV positive?

**[PARTICIPANT]:** I did that because I'm aware that you only have one partner and if I had told that you would have directly known who I am talking about.

**[INTERVIEWER]:** What if I told you that I had 5 partners?

**[PARTICIPANT]:** it's okay, doesn't matter how many you have as long as my client said that you are the only partner he has. I'll just tell you to come and let's talk.

**[INTERVIEWER]:** I want to tell you something that I haven't told anyone, the first time I had a lot challenges when it comes to contacting and notifying the partners of my client because I didn't know where to begin but after I had an interview with the first client I got some ideas from his suggestions and that's why I got an easy time contacting your partners. Though one of your partners didn't come he got tested where he was and gave me the feedback. Today you taught me that it's better not to start talking about partners and telling the client about them being at risk because he might have just one partner and that ill disclose who gave me their information.

**[PARTICIPANT]:** Yes that's it.

**[INTERVIEWER]:** Let's pretend that you are the counsellor, you have told me about my HIV status, told me about the ARVs and I'm conversant with that. Now I want you to talk to me about my partners and contacting them, how are you going to begin?

**[PARTICIPANT]:** I'll start by asking you if you got infected through an accident.

**[INTERVIEWER]:** No I didn't it's through sex.

**[PARTICIPANT]:** Okay and how many sexual partners do you have?

**[INTERVIEWER]:** To be honest I have so many partners whom I frequently have sex with are 18 but there others that were one night stands that I don't remember them.

**[PARTICIPANT]:** Do you want then to be contacted and get tested for HIV too or tell them about your HIV status?

No I don't, for me just knowing my HIV status is enough for me.

**[PARTICIPANT]:** I think it is good for them to be contacted and get tested too so that they can know their HIV status and the chances of spreading the HIV. We can prevent re-infecting each other, it's going to be dangerous to you who will be using the ARVs. So it's fair and safe for you and your partners to be aware of their HIV status.

*(End of the role play)*

**[INTERVIEWER]:** Thanks, so that how I can introduce PNS (*both chuckling*). How do you think we can build trust and confidentiality of the PNS?

**[PARTICIPANT]:** By assuring to the client that his/ her name will not be mentioned in the conversation that the healthcare service provider will have with the partners. By also giving examples on the cases that you have handled and that it's something you are used to doing. And that whatever is said between you two will be confidential.

**[INTERVIEWER]:** What is your opinion and recommendation of the PNS to the GBT?

**[PARTICIPANT]:** It is important and everything is okay the way it is.

**[INTERVIEWER].** Thank you very much for your contribution, Is there anything else you would like us to discuss about PNS and GBT?

**[PARTICIPANT]:** No, I have nothing else to discuss.

**[INTERVIEWER]:** thank you very much for coming and dedicating your time for sharing your views and experiences, I truly appreciate.
