# Supplementary material for: Peer-assisted HIV partner notification services to strengthen index partner testing for newly diagnosed men who have sex with men in coastal Kenya
Source: PLoS One. 2025 Oct 7;20(10):e0333707. doi: 10.1371/journal.pone.0333707 (PMC12503256; doi:10.1371/journal.pone.0333707)
Supplement: S3 Appendix — (ZIP) [file pone.0333707.s003.zip › Deidentified IDI Transcript_1434.docx]

**Participant characteristics:**

Age: 35-39

Sexuality: Bisexual

Education level: Primary

Days between enrollment and IDI: 67 days

Mobilization strategy: OST

Final PNS Strategy: HCP/PM

**Partners identified: 3**

**[INTERVIEWER]**: Welcome, how a you doing?

**[PARTICIPANT]**: Thanks, am doing fine.

**[INTERVIEWER]**: We would like to discuss with you about partner notifications services. we have little knowledge about PNS, and we are trying if we can improve awareness or we see if we can cooperate in future to make this service to reach everyone without any problems, is it okay?

**[PARTICIPANT]**: Yes

**[INTERVIEWER]**: first we encourage every person who test HIV positive to inform their partners to come for HIV testing. We have many ways of notifying the partners; The positive partner notifies his partners to come for HIV testing, the other way is the HIV positive partner(index)is assisted by the counselor in contacting the partners through phone calls where the index partner gives the counselor the telephone numbers of his sexual partners and the counselor calls them to come for HIV testing. The third way is the index partner takes self-test kits from the clinic to go and give to his sexual partners to test them and know their HIV status. The Fourth way is when we have the peer mobilisers to assist the index partner to distribute self-test to the area where the sexual partners of the index partner lives where it maybe the sexual partners may be one of the people who will be given the self-test kits for testing as they will be distributed randomly. So, we have little knowledge about PNS that's why we are requesting the index partners to mention all the sexual partners they have had sex for the last one year to inform them to go for HIV testing, if they test HIV positive they are started on ART and if they test negative they are started on Prep to prevent them from getting infected. Any questions from what I have explained you?

**[PARTICIPANT]**: No questions.

**[INTERVIEWER]**: so, we would like us to discuss more on how you knew your HIV status from the day you tested and realized you were HIV positive and what was your experience and how you are feeling now since you tested HIV positive, now that you were not infected and now you have been infected?

**[PARTICIPANT]**: For now, I can see at least there is a difference. I don't have the energy to stand for a long time because I sometimes feel my legs are weak. I sometimes shiver, most of the time like shocks.

**[INTERVIEWER]**: How did you feel on the first day you tested and realized you were HIV positive?

**[PARTICIPANT]**: I was very scared but I became used of it with time.

**[INTERVIEWER]**: What are the challenges you have experienced up to now?

**[PARTICIPANT]**: For now, I feel am very weak.

**[INTERVIEWER]**: I mean the challenges you have experienced since you knew you were HIV positive, how you are being treated at home now that you are infected or how do you experience life at your home?

**[PARTICIPANT]**: I have not been discriminated so far, but I feel am very weak.

**[INTERVIEWER]**: which are the things you fear most when you knew you have been infected with HIV?

**[PARTICIPANT]**: I don't want to infect my sexual partners with the virus [HIV], that's the biggest fear I have.

**[INTERVIEWER]**: what came in to your mind just immediately you realized you were HIV positive? What thoughts came to your head?

**[PARTICIPANT]**: I was weak and didn't have the morale to live.

**[INTERVIEWER]**: how?

**[PARTICIPANT]**: because I am now sick and I will live with the virus. My life will be very short from now. I won't live like normal people [HIV non-infected people].

**[INTERVIEWER]**: why did you have such thoughts? That you will not live a normal life?

**[PARTICIPANT]**: It depends on the body changes, that's what made me think that way. Sometimes it's easy for someone to notice that I don't concentrate, because I have so many negative thoughts about my life. It is as I have given up on life and I don't think of future projects as my thought is that I can die any time..

**[INTERVIEWER]**: what was the main reason which made you test for HIV?

**[PARTICIPANT]**: I had unprotected sex with a person of unknown HIV status, and that's what made me to go for HIV testing.

**[INTERVIEWER]**: where were you exactly tested?

**[PARTICIPANT]**: I was tested at [HOSPITAL_C].

**[INTERVIEWER]**: was it your first time to test?

**[PARTICIPANT]**: it was my first time, I had not tested before.

**[INTERVIEWER]**: how many sexual partners have you had since you knew your HIV status?

**[PARTICIPANT]**: for now, I don't have sexual partners.

**[INTERVIEWER]**: lets now talk about the oral self-test, where did you meet with the person who gave you the oral self-test, or what did you discuss?

**[PARTICIPANT]**: I wanted to know my HIV status, that's why I was given the oral self-test.

**[INTERVIEWER]**: that's why am asking, how did it start until you were given the oral self-self-kit

**[PARTICIPANT]**: I had come here [MSCH] to know my HIV status that's how I met with the mobiliser, who gave me the self-test.

**[INTERVIEWER]**: after being given the oral self-test what were you explained?

**[PARTICIPANT]**: To test my HIV status.

**[INTERVIEWER]**: and what again did you discuss.

**[PARTICIPANT]**: That is when I tested and realized that I am HIV positive. He [the mobiliser] told me that when you test positive, you are supposed to start ART.

**[INTERVIEWER]**: so, I just want to understand one thing, did you request for the self-test or the peer mobiliser explained to you about the self-test?

**[PARTICIPANT]**: I came to request myself for [HIV] testing.

**[INTERVIEWER]**: what did the mobiliser explain to you how the self-test works? Had you ever used the self-test kit before?

**[PARTICIPANT]**: No, I had never used it before.

**[INTERVIEWER]**: so, did he tell you how it's being used?

**[PARTICIPANT]**: he told me it's used to show your HIV status. He said you rub your gums in the mouth, and the other one [rapid test] you are pricked in the finger to confirm the result.

**[INTERVIEWER]**: how did he explain you about the finger prick?

**[PARTICIPANT]**: he said "if two red lines appear, then you are HIV positive, but if only one red line appears, you are HIV negative.

**[INTERVIEWER]**: Because we know that if a person is tested using the oral self-test and it tests HIV positive it's when he is confirmed with the finger prick.

**[PARTICIPANT]**: I was tested with both test kits, he [the counsellor] said when you are not comfortable with the oral self-test you will confirm with the finger prick testing because they may show different results.

**[INTERVIEWER]**: at the time you were testing were you alone or you were with the mobiliser who gave you the self-test?

**[PARTICIPANT]**: No, I was alone.

**[INTERVIEWER]**: so, you saw the results alone?

**[PARTICIPANT]**: Yes

**[INTERVIEWER]**: Did the mobiliser explain to you about acute HIV. Nowadays there are symptoms which if one experiences just after one to two weeks of infection, that person is HIV positive, were you explained about those symptoms by the peer mobiliser?

**[PARTICIPANT]**: no, he didn't explain to me.

**[INTERVIEWER]**: and maybe you might be knowing the symptoms?

**[PARTICIPANT]**: do you refer to the symptoms which one has recently gone through, or do you refer to symptoms, one was experiencing a long time ago?

**[INTERVIEWER]**: signs of a person who has just been infected.

**[PARTICIPANT]**: No, I have not experienced such symptoms, but I know the signs. I heard that one will have diarrhea, and cough. Thirdly, one will notice changes the way you were, by losing weight from 100kgs to 50 kgs.

**[INTERVIEWER]**: when we talk about AHI symptoms we talk of signs like headache, sore throat, diarrhea, fatigue, vomiting, fever and if you experience such symptoms this shows that you have recently gotten HIV virus, did you experience such symptoms?

**[PARTICIPANT]**: No, I experienced my urine changed color just after one week of testing.

**[INTERVIEWER]**: how did you understand the peer mobiliser at the time he was explaining to you about the self-test, before you had not tested.

**[PARTICIPANT]**: its long time so I have forgotten some of the information.

**[INTERVIEWER]**: what are the ways you experienced with your sexual partners?

**[PARTICIPANT]**: Most of the time you get condoms in sexual partners' house, but you never know if they are safe or they have holes or not, or there may be a lot of frictions and you may not know if you have gotten (acquired) the virus. This may affect you because sexual partners could do all means to infect you if they know they are infected.

**[INTERVIEWER]**: I just want to know from your experience, which circumstances are you passing through your sexual partners? Do you go to their houses?

**[PARTICIPANT]**: yes, I go to their [sex workers'] houses. After you seduce them they tell you instead of going to the guest house, give me the money and let's go to my house so that they may get more money.

**[INTERVIEWER]**: how many times per day or after how long.

**[PARTICIPANT]**: twice per month.

**[INTERVIEWER]**: for the time you are having sex with your sexual partners, are there times you could discuss about condom use? Couple testing?

**[PARTICIPANT]**: no, we don't discuss anything. With the type of sexual partners I have. We don't discuss such. They don't have the habit of following you, it's you who follows them. They are just sex workers.

**[INTERVIEWER]**: did the peer mobiliser discuss with you about fliers, any information he gave you to read about HIV, prep or Acute HIV infection.

**[PARTICIPANT]**: yes, he [the peer mobiliser] told me there are papers you can read.

**[INTERVIEWER]**: can you explain in detail?

**[PARTICIPANT]**: I still have them [information] in my house

**[INTERVIEWER]**: what were the papers talking about.

**[PARTICIPANT]**: About [the fact that] if you are infected you must take [HIV] medication.

**[INTERVIEWER]**: apart from talking about HIV medication, which other things did you discuss concerning the fliers.

**[PARTICIPANT]**: I have even forgotten as you just read them aloud. I didn't record them in my head because they were many but they mostly talked about the disease [HIV].

**[INTERVIEWER]**: how did you react by the time the mobiliser had approached you?

**[PARTICIPANT]**: I had faith in him [the mobiliser], as he told me when you are sexually active this will assist you to maintain good health.

**[INTERVIEWER]:** I just want to know if he approached you, how did you think of him?

**[PARTICIPANT]**: I just believed him as he [the mobiliser] treated me like a doctor. He said if one is HIV positive you must start medication.

**[INTERVIEWER]**: so, what did he tell you the drugs do to your body.

**[PARTICIPANT]**: he [the mobiliser] told me those drugs treat HIV [infection].

**[INTERVIEWER]**: what motivated you to know your HIV status?

**[PARTICIPANT]**: I wanted to know my HIV status.

**[INTERVIEWER]**: what are the things we are supposed to do to motivate people for using the self-test kits.

**[PARTICIPANT]**: people are afraid of going to hospitals to test for HIV, so if people can buy the kits in special places like chemists, and to be distributed in the remote areas it will enable people to access them easily.

**[INTERVIEWER]**: should we distribute them in the remote areas?

**[PARTICIPANT]**: yes, you should also offer self-test kits door to door.

**[INTERVIEWER]**: when you tested and realized you were HIV positive through the self -test, how did you prove your HIV positive?

**[PARTICIPANT]**: I knew my [test] results by seeing two red lines.

**[INTERVIEWER]**: how did you see the it by using the self-test?

**[PARTICIPANT]**: I was not satisfied with the self- test so I wanted another test to confirm. It was with the blood test that I believed that am HIV positive.

**[INTERVIEWER]**: After realizing you were HIV positive did you start ART medication immediately?

**[PARTICIPANT]**: no, I didn't start drugs immediately.

**[INTERVIEWER]**: as from today have you ever started ART medication?

**[PARTICIPANT]**: no, I have not started ART so far.

**[INTERVIEWER]**: what are your reasons for not starting ART?

**[PARTICIPANT]**: I am still waiting for the signs like coughing, diarrhea that's is when I will realize it [the virus] has entered the blood and am very serious sick.

**[INTERVIEWER]**: does it mean that you don't believe if you don't have HIV virus?

**[PARTICIPANT]:** I am 50/50, I have not seen any difference on my body which makes me believe that I have HIV.

**[INTERVIEWER]**: did the counselling you received from the counselor who tested you assist you in any way?

**[PARTICIPANT]**: yes, the counsellor assisted me.

**[INTERVIEWER]**: we would like to discuss more on your sexual partners and after you got tested and realized you are infected with HIV and explained that you can invite them to come for HIV testing?

**[PARTICIPANT]**: The counselor explained to me that when I give the telephone numbers of my sex partners, he can contact them [the sex partners] to come [for testing] because some of them [sex partners], when I tell them about [potential HIV exposure] they may refuse.

**[INTERVIEWER]**: why did you see the reason as to why you give out the telephone numbers?

**[PARTICIPANT]**: for them to know their HIV status so that they can stop infecting others in case they are HIV positive. And, to stop spread of the disease.

**[INTERVIEWER]:** why did you choose the strategy of giving out their telephone contacts?

**[PARTICIPANT]**: most of them [sex partners], if I would tell them about HIV testing, they will refuse and say they have never tested for HIV, but the counselor could counsel them appropriately and make them come for testing as the counselor is a professional.

**[INTERVIEWER]**: maybe do you know whether they were contacted?

**[PARTICIPANT]**: I don't know, because I gave out the number so I am not aware if they [sex partners] were contacted.

**[INTERVIEWER]**: since you gave out the numbers to the counselor, are there any changes to your sexual partners?

**[PARTICIPANT]**: I don't know anything since I gave out the contacts.

**[INTERVIEWER]**: since you gave out the contacts have you ever met with them?

**[PARTICIPANT]**: no, I have not met with them [sex parnters]. I don't know anything from them [sex partners] unless they tell me. The counselor told me that even if he communicates with them he will not tell them that I am the one who gave out the contacts.

**[INTERVIEWER]**: so, since you tested up to now, concerning sexual activities nothing has disturbed you with your sexual partners?

**[PARTICIPANT]**: nothing has disturbed me so far.

**[INTERVIEWER]**: are you still in relationship with these sexual partners?

**[PARTICIPANT]**: I separated all of them.

**[INTERVIEWER]:** suppose we have one sexual partner we have never contacted; how could you help us contact?

**[PARTICIPANT]:** I would take the responsibility of taking the [HIV] self-test kit to give her/him? and tell her/him? that HIV is mostly transmitted through sexual contact so before we continue with this relationship he/she has to take the self-test, just orally.

**[INTERVIEWER]**: now I want us to discuss about disclosure of your HIV status to your family members. Since you knew your HIV status, is there anybody you disclosed your HIV status to?

**[PARTICIPANT]**: no, I have not disclosed to any person.

**[INTERVIEWER]**: what reasons do you have that you have not disclosed your status to anybody.

**[PARTICIPANT]**: most of them will start avoiding you, others will tell you that you have bad behaviors and finally they may start discriminating you. They see you as a bad person. Although there are drugs, they will not treat you like before.

**[INTERVIEWER]**: does these apply to both family members and sexual partners?

**[PARTICIPANT]**: yes, this applies to my family members For sexual partners this does not apply so much because most of them are infected and they are sex workers. Most of the sexual partners may decide to tell you to have unprotected sex, even if you want to use condoms. They say it's an offer for you, but maybe they know their HIV status. I have sometimes been told such a way. I have one person with whom I use protection most of the days, but surprisingly on that day she says we do it without any protection. But I refused as I didn't want to have sex without protection.

**[INTERVIEWER]**: for the sexual partners are there any people you have disclosed your HIV status?

**[PARTICIPANT]**: no, but most of them they are sex workers so they know their HIV status and most of them they spread it [the HIV infection] intentionally.

**[INTERVIEWER]**: suppose we give you the responsibility to disclose to your sexual partners, how could you think they would respond?

**[PARTICIPANT]**: we could quarrel about that, they can ask me how I got to know. They could say that I am the one who infected them, and they [sex partners] could break our relationship completely.

**[INTERVIEWER]**: why do you see there is importance of telling your sexual partners to come for testing?

**[PARTICIPANT]**: for them [sex partners] to get to know their HIV status, whether they are infected or not. If they are infected to be on ART and if they are not infected to start prep.

**[INTERVIEWER]**: why do you think we had to talk about the sexual partners you have had for the last one year?

**[PARTICIPANT]**: so that we know how we could assist [with an HIV test] them. And for the past one year, it is a long period of which it is hard to remember all the sexual partners you have had, especially as most of them are sex workers.

**[INTERVIEWER]**: apart from those you mentioned during the first day, are there others you never mentioned which you feel today you can mention them?

**[PARTICIPANT]**: yes, there some you get them for business, only after that you delete their numbers as most of them are sex workers, which makes it difficult to remember them. The number can be even 200 sexual partners, but I remember only four which I mentioned during the first visit.

**[INTERVIEWER]**: there are challenges and experiences people face with partner notification, could you tell me the challenges you faced and the ways which made it easy to notify sexual partners?

**[PARTICIPANT]**: through giving out their telephone contacts.

**[INTERVIEWER]:** how did you see this PNS service.

**[PARTICIPANT]**: it's a nice service, the sexual partners should also know their HIV status.

**[INTERVIEWER]**: could you recommend this [PNS] service to others?

**[PARTICIPANT]:** people should go for testing with their sexual partners so that they know their sexual partners' HIV status.

**[INTERVIEWER]**: what do you think this PNS service will bring problems if it continues?

**[PARTICIPANT]**: no, it will not bring any problems because it will be reminding people who would have forgotten [their exposure to HIV], and makes them reduce their sexual risk behaviour.

**[INTERVIEWER]**: what can make it easier for MSM to bring their sexual partners for testing after realizing that they have tested HIV positive?

**[PARTICIPANT]**: through counselling all the MSMS, informing them about PNS; through road shows, and concerts shows.

**[INTERVIEWER]**: as we discussed before there are different methods of PNS, by looking at this paper we have this method that says the counselor will contact the sexual partners of the index partner to come for HIV testing, secondly the peer mobiliser will offer a self-test kit to the index partner to go give his sexual partners. Third the peer mobilisers will distribute the self -test kits to the place where the index partner's sexual partners live, the counselor will assist the index partners in inviting the sexual partners to come for testing. The peer mobiliser will also assist the index partner on inviting the sexual partners to come to the clinic for HIV testing. What do you think about these PNS methods?

**[PARTICIPANT]**: they are good methods

**[INTERVIEWER]**: sure?

**[PARTICIPANT]**: yes, especially the method where the index partner is given the oral self-test to go and give to his sexual partners to test themselves.

**[INTERVIEWER]**: apart from these ways we have mentioned here, do you see any other method for PNS?

**[PARTICIPANT]**: no, I don't know.

**[INTERVIEWER]**: how long did the counselor introduce the partner notification services to you on the first day? Did he tell you on the first day you tested or on another day?

**[PARTICIPANT]**: he told me [about PNS] on another day I came.

**[INTERVIEWER]**: how do you think it's supposed to be, one should be introduced the PNS service on the first day or to be done on another day?

**[PARTICIPANT]**: it should be done on the same day.

**[INTERVIEWER]**: why should it be done on the same day?

**[PARTICIPANT]**: so that he can start [ART] medication.

**[INTERVIEWER]:** can your advice your friends about the PNS services?

**[PARTICIPANT]**: yes, I can tell them [my friends] that they must get to know their HIV status and after knowing [their HIV status], they should invite their sexual partners to go for testing.

**[INTERVIEWER]**: are there any additions or subtractions to the discussion we have had today?

**[PARTICIPANT]**: people should be made aware of the [HIV] services.

**[INTERVIEWER]**: is there anything you feel we have left out and we need to talk about it today?

**[PARTICIPANT]**: nothing, am happy with the interview and the time we have had together.

**[INTERVIEWER]**: thanks so much for the time you have given me, if you have anything you are free to come and share or if there are any new information or answers from this study, and what you have shared will really help us understand and improve PNS services. Feel free to come any day you wish and today should not be the only day for you to come to our clinic, also you can tell your friends about the services we offer and we give self-test kits free of charge in our clinic and we give services to both key population and general population but we give priority to key population . Thanks, you so much.
