# Supplementary material for: Peer-assisted HIV partner notification services to strengthen index partner testing for newly diagnosed men who have sex with men in coastal Kenya
Source: PLoS One. 2025 Oct 7;20(10):e0333707. doi: 10.1371/journal.pone.0333707 (PMC12503256; doi:10.1371/journal.pone.0333707)
Supplement: S3 Appendix — (ZIP) [file pone.0333707.s003.zip › Deidentified IDI Transcript_1522.docx]

**Participant characteristics:**

Age: 25-29

Sexuality: Gay

Education level: Secondary

Days between enrollment and IDI: N/A days

Mobilization strategy: Open B

Final PNS Strategy: HCP/PM

**Partners identified: 2**

**[INTERVIEWER]:** welcome to today's interview on [DATE]. Thank you very much for coming for the interview and agreeing to participate in this interview. As I had mentioned earlier, we will tape record this interview so that we will be able to capture every opinion, thoughts and ideas when we write a report. How are you feeling?

**[PARTICIPANT]:** I'm feeling ok emotionally, mentally and health wise I'm ok.

**[INTERVIEWER]:** Ok tell me more about the mentally, physically emotionally health wise. Was there a time you felt different?

**[PARTICIPANT]:** I faced some challenges when I found out that I was HIV-positive. I was worried and emotionally down because of the major change in my life. It wasn't easy at all, knowing that I was used to test Negative and now got infected.

**[INTERVIEWER]:** Those were the things that were affecting you emotionally, mentally and health wise....

**[PARTICIPANT]:** Before I tested positive I was very sickly and still after knowing my new HIV status the illness was still present and both combined the illness and HIV test results were a heavy burden for me.

**[INTERVIEWER]:** What were you suffering from?

**[PARTICIPANT]:** Malaria, dizziness feeling weak lack of appetite and chronic diarrhoea

**[INTERVIEWER]:** When you were sick did you suspect anything?

**[PARTICIPANT]:** No, I did not suspect anything I just went to the hospital and I was admitted in the hospital.

**[INTERVIEWER]:** For how long were you in the hospital?

**[PARTICIPANT]:** I was there for 3 weeks

**[INTERVIEWER]:** What treatment did you received?

**[PARTICIPANT]:** They just gave me medicine and got me admitted they were just testing for malaria

**[INTERVIEWER]:** You said that right now you are feeling better than how you were when you first tested and found out that you were HIV-positive, what contributed to feeling better?

**[PARTICIPANT]:** I received a lot of support, encouragement and counselling that really helped me get better. I was given information on HIV/AIDs and how ARVs helps to improve someone's well-being and lead to a positive healthy life.

**[INTERVIEWER]:** What made you get tested for HIV?

**[PARTICIPANT]:** Previously, when I used to suffer from malaria I never really used to diarrhoea a lot but this time things were different. I suspected myself because of the prolonged sickness. I also lost a lot of weight in a very short time, loss of appetite also added to that and not getting better even with the treatment I received. This troubled me and forced me to come and get tested. Am also in research that require regular testing. Initially I used to get tested after a month but things changed to three months.

**[INTERVIEWER]:** Okay, so although all those symptoms and signs that you had pushed you to get tested but you also got tested and research participation...

**[PARTICIPANT]:** Yes, although the change from monthly to quarterly was not good at all.

**[INTERVIEWER]:** What do you exactly mean?

**[PARTICIPANT]:** Well, the duration is quite long compared to monthly one can easily forget appointment date, beside if you were a regular tester and you stay for long without testing then risk confidence goes up unlike monthly HIV testing always make someone to be keen and worried to engage on risky behaviours as they know they will get tested soon. Also, the 3 months duration make someone not to adhere well to PrEP and can easily be infected.

**[INTERVIEWER]:** Are you talking out of your own experience?

**[PARTICIPANT]:** Yes I am, that's what happened to me unfortunately and it might happen to many more others.

**[INTERVIEWER]:** Okay, thanks for highlighting that, now let's get back to partner notification services, how was PNS introduced to you?

**[PARTICIPANT]:** It's a really good service it really helped me out because I was so confused at that moment it gave me a bit of courage because I wasn't sure who infected me.

**[INTERVIEWER]:** Ok, I want to hear from you how was it introduced to you as in how did you start talking about the PNS?

**[PARTICIPANT]:** It was introduced to me after I found out that I was HIV-positive so as to help me find out who infected me and to have them to know their status and how they will benefit depending on their HIV test results.

**[INTERVIEWER]:** What can you say about the counselling you received on PNS?

**[PARTICIPANT]:** The counselling was good the importance was emphasized and I thought that was great thing to do.

**[INTERVIEWER]:** What is your opinion about the strategies of PNS?

**[PARTICIPANT]:** The strategies were safe and were good and we discussed different ways to get my partners at first, we discussed OST kit this was very helpful because it has a lot of privacy some people have the fear of being seen around gay clinics, the second strategy of giving contacts to health provider to call the partners and asking them to come over.

**[INTERVIEWER]:** Which strategy did you opted for?

**[PARTICIPANT]:** All those Strategies were nice they were favourable but I chose the [RESEARCH_INSTITUTION] provider to contact them.

**[INTERVIEWER]:** What made you choose that strategy?

**[PARTICIPANT]:** I think it was better and safest for me as it was anonymous.

**[INTERVIEWER]:** What are your opinions now about the strategy you choose?

**[PARTICIPANT]:** It made it easier to contact and get my partners.

**[INTERVIEWER]:** Do you still think it's the best method for you and it made things easier for you?

**[PARTICIPANT]:** Yes, I'm ok with the Healthcare service provider method

**[INTERVIEWER]:** Were all of your partners contacted?

**[PARTICIPANT]:** Yes they were all contacted.

**[INTERVIEWER]:** Now we are going to talk about your partners, do you remember the number of partners you mentioned?

**[PARTICIPANT]:** Yes, they were three.

**[INTERVIEWER]:** okay, let's start talking about the first one. How do you think he reacted when he was contacted?

**[PARTICIPANT]:** I'm sure he took his time thought things over and decided to get tested.

**[INTERVIEWER]:** Sure...

**[PARTICIPANT]:** Yes I'm 100% sure.

**[INTERVIEWER]:** What about your second partner, how do you think he reacted when he was contacted?

**[PARTICIPANT]:** Him too I know he didn't had any problems he is a cool guy and very understanding.

**[INTERVIEWER]:** What about the third partner, how do you think he reacted when he was contacted by the Healthcare service provider?

**[PARTICIPANT]:** The third one is a bit scared and very high risk so I know such a call was a red flag for him and could be he was hesitant to be tested.

**[INTERVIEWER]:** Did any of your partners found out that you were the one who gave out their number and encountered issues?

**[PARTICIPANT]:** No, everything went okay nobody knew that it was me, everything was and still fine.

**[INTERVIEWER]:** Has it affected any of your relationships with your partners?

**[PARTICIPANT]:** the third one doesn't want to talk to me anymore

**[INTERVIEWER]:** I want you to tell me more, why did you and your partner decide to end things?

**[PARTICIPANT]:** it's about financial issues.

**[INTERVIEWER]:** So it had nothing to do with the PNS?

**[PARTICIPANT]:** Yes, he was a bit money minded keeping on requesting money from me and yet he is a boda boda working in [CITY_A]. He asks for money every now then, he was just too much and I highly suspect he is the one who infected me. Those two reasons made me annoyed and decided to end things with him.

**[INTERVIEWER]:** Am taking you back to the number of partners, apart from the three partners you mentioned, is there other partners that perhaps you forgot to mention?

**[PARTICIPANT]:** I don't think so, I am sure that there are just three of them.

**[INTERVIEWER]:** Now I'm going to ask you questions about disclosure, have you told anyone about your HIV status since you found out that you are HIV positive?

**[PARTICIPANT]:** No, I have not told anyone

**[INTERVIEWER]:** So you have not told anyone out there. If you were given a chance to tell someone who and why would you tell?

**[PARTICIPANT]:** A friend of mine, really close to me.

**[INTERVIEWER]:** What has prevented you from telling that friend?

**[PARTICIPANT]:** He is far, I'll wait till he comes then I'll tell him.

**[INTERVIEWER]:** What would make you wanting to tell him and not anyone else?

**[PARTICIPANT]:** We don't know what tomorrow holds, I might develops complication and no one would understand, I trust he'll be the one to tell my parents and close people in life about my HIV status. Am comfortable with him only because there are myths and misconception as well as stigma, am comfortable telling him because he is also HIV positive and has some facts about HIV.

**[INTERVIEWER]:** How do you think telling him about your HIV status can affect your relationship with him?

**[PARTICIPANT]:** It can't because he is also infected with HIV he will understand.

**[INTERVIEWER]:** Just a by the way question, why haven't you told anyone yet?

**[PARTICIPANT]:** I'm from a religious family, if I disclose to them they might stop me from taking ARVs and take me to a religious leader to be prayed for. They would prefer the religious way.

**[INTERVIEWER]:** Is that the only reason?

**[PARTICIPANT]:** Yes that's the only reason, my mother will react so badly because she's very religious.

**[INTERVIEWER]:** Apart from family members, why haven't you decided to tell anyone else?

**[PARTICIPANT]:** I don't want to tell anyone, because I just can't trust anyone, I might get some stigma if my family gets to find out because I have an aunt who is also HIV positive she has been facing stigma from her own family.

**[INTERVIEWER]:** In the Kenyan guidelines they recommend discussing with a newly diagnosed person about their partners the past 12 months, in your opinion do you think it was easy to remember the people you've been with sexually in the past 12 months?

**[PARTICIPANT]:** It wasn't easy, because I have a lot of partners and 12 months is a long time considering that I'm am a sex worker I can't be sure of the exact number of partners.

**[INTERVIEWER]:** How easy was it for you to discuss your partners for the past 12 months?

**[PARTICIPANT]:** it was hard for me, because I was testing regularly until last month so I only mentioned the ones that I had sex with a month before learning my HIV status, the others it was hard to remember them and I had sex with them when I was still negative.

**[PARTICIPANT]:** What is your feeling about notifying partners?

**[PARTICIPANT]:** First, it will help prevent the spread of HIV, for instance someone is seeing the signs and symptoms and he/ she is not in the research and if that person is contacted and told that he/ she is at risk because one of his/ her recent partners has been found HIV positive, this will make that person curious and he/ she will get tested for HIV, this will help him/her prevent the spread by knowing their status.

**[INTERVIEWER]:** What are the benefits of PNS?

**[PARTICIPANT]:** It helps both the client and partners in knowing their HIV status and whether they are infected or not they will get help either way. The infected individual will start the ARVs dose early or in good time and will get counselling while the uninfected individual will be told of preventive measures so that they don't get infected.

**[INTERVIEWER]:** What are some of the preventive measures?

**[PARTICIPANT]:** Using PrEP most of the times.

**[INTERVIEWER]:** What are the challenges in the PNS?

**[PARTICIPANT]:** The first challenge is when contacting partners they might not come, risk of them wanting to know who gave out their phone numbers and getting violent.

**[INTERVIEWER]:** How do you think we can increase PNS uptake to MSM?

**[PARTICIPANT]:** I think using the health care service provider and being honest about the number of partners.

**[INTERVIEWER]:** When was PNS introduced to you?

**[PARTICIPANT]:** That same day that I found out that I was infected.

**[INTERVIEWER]:** What do you have to say about PNS being discussed on the same day one is found to be HIV positive?

**[PARTICIPANT]:** They should give it more time, for example a week or at least 3 days.

**[INTERVIEWER]:** Why not that very day?

**[PARTICIPANT]:** When using the healthcare service provider strategy risk might occur. If I'm from having sex with one of partners just yesterday night and today I tested positive and they are contacted today, that partner might figure it out that it's me especially if the partner has only one sexual partner. Theirs a high risk of the client being exposed.

**[INTERVIEWER]:** So you mean to say that the client should be introduced to PNS after 3 days and that the partner should be contacted after a week or so.

**[PARTICIPANT]:** Yes

**[INTERVIEWER]:** This is a new opinion, thank you very much for that. What is your general feeling about the message we communicate to partners of HIV exposure?

**[PARTICIPANT]:** It is on point, when you call someone just tell him/her to come and get tested they will not take it seriously, but telling them that they are at a risk of being infected, they will take it seriously and come or test elsewhere.

**[INTERVIEWER]:** Don't you think that this partner will be scared because a stranger just called to tell him that he should get tested because they exposed themselves having sex with someone who was diagnosed with HIV?

**[PARTICIPANT]:** No problem at all, no can just call for fun and deliver such a message, well...they might be reluctant at the beginning but believe me they will test either come here or go somewhere else.

**[INTERVIEWER]:** How can we build trust and confidentiality when it comes to PNS?

**[PARTICIPANT]:** Just keeping your promise by assuring them that you won't mention their names or any hint that could be an identifiers. You can also share the success stories of how it was done to others and it didn't cause any harm.

**[INTERVIEWER]:** What can you recommend about PNS to MSM?

**[PARTICIPANT]:** It should be introduced to other hospitals too, MSM CBOs so that a lot of people can be reached and get to know their status.

**[INTERVIEWER]:** Is there anything else you would like to add?

**[PARTICIPANT]:** No, I have nothing else to add.

**[INTERVIEWER]:** Thank you very much for coming and dedicating your time for sharing your views and experiences. We have come to the end of our discussion, thank you very much.
