# Supplementary material for: Peer-assisted HIV partner notification services to strengthen index partner testing for newly diagnosed men who have sex with men in coastal Kenya
Source: PLoS One. 2025 Oct 7;20(10):e0333707. doi: 10.1371/journal.pone.0333707 (PMC12503256; doi:10.1371/journal.pone.0333707)
Supplement: S3 Appendix — (ZIP) [file pone.0333707.s003.zip › Deidentified IDI Transcript_1549.docx]

**Participant characteristics:**

Age: 35-39

Sexuality: Bisexual

Education level: Other

Days between enrollment and IDI: 53 days

Mobilization strategy: OST

Final PNS Strategy: HCP/PM

**Partners identified: 4**

**[INTERVIEWER]**: welcome to our todays discussion

**[PARTICIPANT]**: Thank you

**[INTERVIEWER]**: today is [DATE], our discussion is taking place in [CITY_A] and your number is 1549 if am not wrong but we shall confirm that later

**[PARTICIPANT]**: ok

**[INTERVIEWER]**: just to remind you on what we will be discussing today. Our research is based on follow up on the infected partner

**[PARTICIPANT]**: true

**[INTERVIEWER]**: after getting tested and find out that you are infected, we would like to also know the partners that you have been with and also get them to know their status.

**[PARTICIPANT]**: ok

**[INTERVIEWER]**: the client who is infected we call him the index patient

**[PARTICIPANT]**: ok

**[INTERVIEWER]**: and also, this initiative of following up on partners is voluntarily and is not by force, so just put in mind that its not by force and if you agree to it then we will di it.

**[PARTICIPANT]**: ok

**[INTERVIEWER]**: it is done in health facilities by counsellors and other health care workers and during follow up we have two ways that we use in order to get the partners that have been infected, one is by talking to you to inform your partner to come for testing, the other way is through a health care worker to assist you in communicating to your partner to come for testing

**[PARTICIPANT]**: so that she can get assistance

**[INTERVIEWER]**: yes, so that she gets to know her status

**[PARTICIPANT]**: ok

**[INTERVIEWER]**: so, when we use the health care worker we uphold confidentiality and don't mention who directed us to her or gave us her number

**[PARTICIPANT]**: to avoid problems

**[INTERVIEWER]**: yes, so confidentiality is very important

**[PARTICIPANT]**: ok

**[INTERVIEWER]**: so this is not a new thing its has been going on in all government hospitals when you are tested and found out to be positive we would like to follow up on your partners so that we get them to be tested, we say its important for people to know their status

**[PARTICIPANT]**: ok

**[INTERVIEWER]**:so its something that has been happening on a regular basis because we still don't have clear understanding of the people who are at a risk of HIV infection. When I talk about the "people" we talk about the people who have sex with other men, bisexuals or transgenders

**[PARTICIPANT]**: transgender is someone who......

**[INTERVIEWER]**: a transgender is a man who considers himself a woman or aspires to be a woman, you will find that they even take medication in order to turn themselves into women

**[PARTICIPANT]**: women

**[INTERVIEWER]**: yes have you seen such men

**[PARTICIPANT]**: yes I have seen them

**[INTERVIEWER]**: so those are the transgenders

**[PARTICIPANT]**: ok

**[INTERVIEWER]**: so in that category we still don't have a clear understand how this can be done but among the normal category of people its something that is happening and we have a clear understanding. So the aim of this research is to get your opinion about on how PNA can work best amongst MSMs

**[PARTICIPANT]**: MSMs

**[INTERVIEWER]**: so we will be learning from each other ok

**[PARTICIPANT]**: its ok

**[INTERVIEWER]**: so how have you been since the day you were tested and knew your status?

**[PARTICIPANT]**: since the day you tested me I haven't seen any changes I see myself doing well and after you started me on medication I see a lot of improvement

**[INTERVIEWER]**: you have improved in what ways?

**[PARTICIPANT]**: by using my medication as prescribed and the stipulated time because according to my work schedule, I wake up very early in the morning and I cant take the medication in the morning I take them at 6 in the evening and since I started taking them I can see my health is improving

**[INTERVIEWER]**: have you had any challenges?

**[PARTICIPANT]**: what kind of challenges?

**[INTERVIEWER]**: any that you have experienced since you started using medication

**[PARTICIPANT]**: since I started using medication, I haven't had any challenges

**[INTERVIEWER]**: and what were the reasons that led you to come and get tested?

**[PARTICIPANT]**: (clears throat) the reason as to why I decided to come and know my status is it reached a time when I started experiencing fever at ten in the morning a time like this and also coughing. It reached a point I started diarrheaing , and so I decided to go to the chemist and get some medication but I didn't see any improvement that is when I decided to come to hospital to get help. So on reaching at the general hospital they told me I have to get tested so that when know what we are treating you for.

**[INTERVIEWER]**: ok

**[PARTICIPANT]**: so when I came here I got some counseling, got tested and accepted my status and also accepted to take the medication. After starting the medication, the issues of diarrhea and fever stopped.

**[INTERVIEWER]**: when you first came you used tested using the gadget that uses saliva? (oraquick)

**[PARTICIPANT]**: yes I used the one that you put in the mouth

**[INTERVIEWER]**: in the mouth?

**[PARTICIPANT]**: yes

**[INTERVIEWER]**: can you tell me who gave you the gadget? Who gave you that gadget?

**[PARTICIPANT]**: who gave me that gadget?

**[INTERVIEWER]**: yes?

**[PARTICIPANT]**: ok I have forgotten the name of the person that gave me the gadget

**[INTERVIEWER]**: it is ok

**[PARTICIPANT]**: but he started persuading me when we were at the gym and he also gave me the gadgets at the same place

**[INTERVIEWER]**: what we mainly wanted to capture is in our research is, we have peer educators also known as mobilizers whom we gave the oral gadgets in order to give to their peers so that test and get to know their status. So I wanted to know how he persuaded you until you took the oral gadget for testing. What conversations did you have until you decided to take the oral gadget?

**[PARTICIPANT]**: so while at the gym, I was un able to lift some weight and could only stare at others lifting weight until they asked me what the problem was that's when I told them and diarheaing and also have fever and I would like to know my status because I have done a lot so that is when this guy, what is his name by the way?

**[INTERVIEWER]**: I understand who you are talking about, you said you meet at the gym?

**[PARTICIPANT]**: yes

**[INTERVIEWER]**: remember it is not ethical to mention names during interviews

**[PARTICIPANT]**: so I met him at the gym and he told me that times are not as good as they were before and knowing your status is very good so that is when he told me tomorrow I will come with some gadgets that you can use to test and get to know your status and after you get to know your status that is when I can advise you to go to the general hospital

**[INTERVIEWER]**: so what ways do you feel are best to advise MSMs? So that the can come and know their status?

**[PARTICIPANT]**: you befriend them more so that you are able to explain to them in depth

**[INTERVIEWER]**: so you are saying we have to be close to them?

**[PARTICIPANT]**: yes we have to be close to them

**[INTERVIEWER]**: so when you tested with the oral gadget and got your results, what went through your mind? Or lets start by how used the gadget. What was the experience like when you used the oral gadget?

**[PARTICIPANT]**: so they put the gadget in my in part of the mouth and they tested and the results weren't good

**[INTERVIEWER]**: and after that what happened?

**[PARTICIPANT]**: the results weren't good

**[INTERVIEWER]**: after finding out that the results weren't good you came to the hospital for further assistance?

**[PARTICIPANT]**: yes I came the first day for the oral test then I came back the second day for the other tests.

**[INTERVIEWER]**: were you able to start medication at that same time?

**[PARTICIPANT]**:when I came for the oral test, I wasn't given any medication but on the second day after the other tests I was given septrine for one week and after I finished the septrine I came back and they told me to draw some blood I think it was for CD4 or something

**[INTERVIEWER]**: ooh there are those tests that they conduct first that is after they open the file for you to start medication

**[PARTICIPANT]**: yes

**[INTERVIEWER]**: the tests are called baseline so as to establish how our client is fairing on

**[PARTICIPANT]**: yes

**[INTERVIEWER]**: so that they know which medication to start you on.

**[PARTICIPANT]**: so they explained to me that they cant start me on medication until the tests are done because the medication available isn't the old type so its important to test you so that we establish the medication to give you that is when they conducted the tests on Thursday and told me to come take my results on Monday and that's when they started me on medication.

**[INTERVIEWER]**: ok so did you see the importance of starting medication early? Or what gave you the moral because you went and came back severally but wasn't discouraged?

**[PARTICIPANT]**: I wasn't discouraged because I said I would rather start my medication early before my body could get worse

**[INTERVIEWER]**: did you meet a counselor before starting on your medication?

**[PARTICIPANT]**: I met a counselor when I came and feeling well

**[INTERVIEWER]**: ok and did the conversation between you and the counselor have any benefits in your life? Or in your opinion the conversation you had with the counselor, were they beneficial?

**[PARTICIPANT]**: the counselor saw the status I was in and asked me what was happening? And I told him I had a fever and diarrhea and he suggested that I come to hospital. So during that conversation I saw sence and that why I decided to come to hospital and there is a Swahili saying that goes "when you feel so ill you must see a doctor" that is why I decided to come see a doctor

**[INTERVIEWER]**: ok I understand, so I would like to talk about the partners that you talked about the last time you came

**[PARTICIPANT]**: ok

**[INTERVIEWER]**: so can you remember how many partners you told the counselor?

**[PARTICIPANT]**: the first day?

**[INTERVIEWER]**: yes

**[PARTICIPANT]**: both male and female or in general?

**[INTERVIEWER]**: in general

**[PARTICIPANT]**: in general they were about 12

**[INTERVIEWER]**: and were all of them found or others couldn't be found

**[PARTICIPANT]**: I don't have phone contacts for all of them

**[INTERVIEWER]**: so when you were tested and found to be positive, what was your take the issue of partner notification? Was it addressed on time or the counselor should have waited a little?

**[PARTICIPANT]**: it was addressed on time

**[INTERVIEWER]**:what were the benefits on your side?

**[PARTICIPANT]**: benefits on my side?

**[INTERVIEWER]**: yes

**[PARTICIPANT]**: the benefits are I can sit down with others and tell them to come and get to know their status

**[INTERVIEWER]**: and are their any of your partners that you have shared your status with?

**[PARTICIPANT]**: I have told any

**[INTERVIEWER]**: so you chose the counselor to contact the partners?

**[PARTICIPANT]**: yes something like that, because I find it hard telling someone to go to hospital they might ask you why should I go to hospital? So that is a bit hard they might even say you are sleeping with me and you know you are sick

**[INTERVIEWER]**: now I understand fully so that is when you chose a counselor to do the follow up and counsel them

**[PARTICIPANT]**: yes to look for then and counsel them through the phone contacts I gave, the counselor can contact them and counsel them

**[INTERVIEWER]**: so at that time you thought that was the ideal method?

**[PARTICIPANT]**: I see that being the ideal method

**[INTERVIEWER]**: so up to now you feel that is the ideal method?

**[PARTICIPANT]**: yes that is the best method according to me because I don't have any other method.

**[INTERVIEWER]**: ok and amongst your partners is any that has been contacted and came and knew her status?

**[PARTICIPANT]**: I have no idea

**[INTERVIEWER]**: you have no idea?

**[PARTICIPANT]**: yes

**[INTERVIEWER]**: ok so no one has told you they were contacted to come and know their status?

**[PARTICIPANT]**: there are two people who saw when I came to the VCT to take my medication and asked me what I had come to do at the VCT

**[INTERVIEWER]**: and they were your partners?

**[PARTICIPANT]**: yes

**[INTERVIEWER]**: were they male or female?

**[PARTICIPANT]**: they were female and I said I had been called by a friend, I had done some work for him and he told me to come here and take my money that's why you saw me there and now am heading home but one of them had a very big question mark and when we got home she asked me twice and I told her if you don't believe I went their to take my money then lets arrange we go together and know our status. And she told me if you want to go you go so can you really force such a person?

**[INTERVIEWER]**: so she had a lot of questions?

**[PARTICIPANT]**: she had a lot of questions

**[INTERVIEWER]**: so according to this follow up there is a possibility that some of your partners came and some didn't

**[PARTICIPANT]**: yes

**[INTERVIEWER]**: so with in the last one month has their been any changes in your relationship with these partners?

**[PARTICIPANT]**: you know we used to either communicate via phone or just meet abruptly and I said I will continue hurting myself if I continue indulging in sex because if I indulge in sex and at the same time use this medication, I see like they wont work properly that's why I decided to choose one, eat properly, take my medication, work out and relax

**[INTERVIEWER]**: so you have abstained a little on sex?

**[PARTICIPANT]**: yes I have abstained a little on sex

**[INTERVIEWER]**: were you given the oral test kits to go and test with your partners?

**[PARTICIPANT]**: yes I was given one oral test kit and went and used it on one of the partners only to find out that she was using medication only that she was taking them from a different hospital. We tested together and then and she said she didn't know I was positive and I told her I also didn't know she was positive and I showed her my card and she showed me her card but she takes her medication from the [HOSPITAL_E]

**[INTERVIEWER]**: so one of your partners was already on medication but you didn't know?

**[PARTICIPANT]**: yes

**[INTERVIEWER]**: so when you went with the oral test and tested that is when you realized?

**[PARTICIPANT]**: yes that is when I realized and she told me don't worry am also like that but my question is, how many types of virus their?

**[INTERVIEWER]**: so are you asking me that question now or she asked you then?

**[PARTICIPANT]**: she asked me then and I told her I see the virus being just the virus nothing more and that there is no big or small virus (laughing)

**[INTERVIEWER]**: (laughing)

**[PARTICIPANT]**: and I dont know if there is a big virus or small virus all I know is its just a virus

**[INTERVIEWER]**: ok

**[PARTICIPANT]**: and she said there is a type of virus that makes you hair fall off and the other that makes ones lips look like they burned (laughing)

**[INTERVIEWER]**: (laughing)

**[PARTICIPANT]**: so I told her that all I know is its just a virus but she told me its said that there are A,B,C type of viruses and if you have the A virus and I have C virus then they cant mix and there is a possibility that the medication you are using and the medication am using is different. So we were discussing the same way we are discussing with you

**[INTERVIEWER]**: ok

**[PARTICIPANT]**: so we went ahead until we started laughing at one another and saying that if I have A then you have added me C

**[INTERVIEWER]**: (laughing) so if am to touch on it a little there are two types of viruses there is HIV 1 and HIV 2, you will get that some people have HIV 1 and some have both HIV1/2. I don't know if you can remember when you were tested we used a first test kit that was kind of a paper and the other was plastic

**[PARTICIPANT]**: I remember very well and the third one was the oral one

**[INTERVIEWER]**: yes and the plastic one is written somewhere 1 and 2

**[PARTICIPANT]**: H1 and 2

**[INTERVIEWER]**: yes and its written C somewhere if am not wrong so if you put the blood sample plus the buffer a line might appear on C and number one. If it shows that then it means you have HIV1, others show 3 lines on C,1 and 2 then it means that person has HIV1 and 2 so we say even if you are tested and found that you are positive it doesn't mean that is the end of everything and that you cant be infected again because after all you are HIV positive, there is still some danger because you might find someone with a different type of HIV and if you continue having sex recklessly you will be adding more viruses to your body. We say there is infection and re infection so if you add a different type of HIV your body continues to weaken

**[PARTICIPANT]**: and is the medication the same or different?

**[INTERVIEWER]**: HIV medication is different there is first line and second line treatment so they know how they issue the medication and that is why after every 6 months they check the viral load in your body and also when they are opening the file they take a blood sample to check the CD4 so that they know what medication to give you

**[PARTICIPANT]**: so that is where my problem is because you might be taking medication for HIV1 and you also have HIV2 and the HIV 2 might be killing you

**[INTERVIEWER]**: they always know the type of medication to give and if it will work

**[PARTICIPANT]**: ok I have understood you

**[INTERVIEWER]**: so is their any other partner that you have shared with apart from her?

**[PARTICIPANT]**: no its only her

**[INTERVIEWER]**: and is their anyone else you would like to know about your status but you don't know how to go about it?

**[PARTICIPANT]**: yes there is a lady the we fell in love with each other but the problem is she insists she wants us to come and test but I told her that I was tested at the general hospital so if we are to test we have to come to the general hospital but she doesn't want she wants us to go at [HOSPITAL_A]

**[INTERVIEWER]**: is it a hospital?

**[PARTICIPANT]**: yes but at [HOSPITAL_A] hospital we have to use money and she wont give out the money I will so why not just go to the general hospital? So we still argue a lot with her on testing. She loves me a lot but am HIV positive how do I make her understand me?

**[INTERVIEWER]**: so how far are you in terms of testing with her?

**[PARTICIPANT]**: she still insists we test because she works for women groups and she is often called in different places and her day off is on Sunday after church at 2

**[INTERVIEWER]**: then you will have to take the oral test kit. That's why current the kits are available so that you can test at home. If she is that busy and cant come to the hospital then the oral self test kit is good for her. You can come and ask for the oral test kits we give and you go test.

**[PARTICIPANT]**: so if I have the oral test kit and we go test at home, to me I feel testing at home isn't helpful, the best help is through the doctor because even if we test at home she still has to come to hospital for medication. It will be ok if she is not positive 30:05 but what if she turns positive? You know am not a doctor and I will get the best counseling from a doctor because he has a way of explaining to her so that she can accept her status and start medication but I might disclose to her the status and she ends up hanging her self

**[INTERVIEWER]**: but you can test together and she gets to know your status and her status then after that she can come to hospital and am sure if she finds out she is positive she will find time and come to hospital so you will have fulfilled your obligation. Though you would love a counselor to be present but if he is not available cant be able to reach where you are that's when we use the oral test kits. The most important thing is for someone to test and know their status first. She will definitely find time and come

**[PARTICIPANT]**: I have understood you

**[INTERVIEWER]**: so if you are still interested then after our discussion I can give you the oral test kit

**[PARTICIPANT]**: ok

**[INTERVIEWER]**: but its voluntarily

**[PARTICIPANT]**: I would love that

**[INTERVIEWER]**: so you are saying since your partners have been notified about testing their has been no great change?

**[PARTICIPANT]**: no their hasn't

**[INTERVIEWER]**: nothing like it is you who infected me

**[PARTICIPANT]**: no that hasn't happened

**[INTERVIEWER]**: so apart from you partners have their been any change at home, at work or in the neighborhood that you stay? Has anyone known about your status.

**[PARTICIPANT]**: you know when you come to such a place, there are neighbors, friends who you meet here but no one knows about my status

**[INTERVIEWER]**: so like I said PNS is a normal thing and if you are found to be positive we like to discuss the partners you have had in the last one year so that they are also tested and know about their status and if they are found to be positive then we tell them to start their medication early and if one is negative then we tell them on how to protect themselves. Now a days there is prep used by someone who is negative so that they protect themselves from getting HIV. So we are saying that prevention methods are many and we all choices on which one to use. There are some who say they will use condom all the time and there are others who say they will take medication as a method of prevention, and there is also that person that says he doesn't want condom or medication because they only have one partner. Talking to your partners isn't that easy and you told us you had 12 partners though you couldn't remember all their contacts but the ones that you talked about are they many?

**[PARTICIPANT]**: yes they were many

**[INTERVIEWER]**: and how did you open up to the counselor about your partners?

**[PARTICIPANT]**: I just felt like I should explain myself

**[INTERVIEWER]**: you explain yourself on what benefit? Did you see any importance of talking about the partners?

**[PARTICIPANT]**: I thought if I explained myself I would get proper support from the doctor

**[INTERVIEWER]**: apart from the ones that you have talked about are their others that you might have forgotten and you feel its important to talk about them today?

**[PARTICIPANT]**: I think they are the only ones

**[INTERVIEWER]**: are their any new partners?

**[PARTICIPANT]**: I haven't heard any new partners

**[INTERVIEWER]**: you haven't gotten any new partners?

**[PARTICIPANT]**: yes

**[INTERVIEWER]**: we are about to end our discussion. So in your opinion is their any other way we cane communicate to MSMs so that they can come out and their partners tested and get to know their status? What can we do as health care workers in order to for them to come out and bring their partners for testing?

**[PARTICIPANT]**: by making follow up and counseling them

**[INTERVIEWER]**: what would you like us to counsel them on? Or what should we tell them that will make them come?

**[PARTICIPANT]**: I don't know

**[INTERVIEWER]**: we said their isn't any right or wrong answer

**[PARTICIPANT]**: ok

**[INTERVIEWER]**: so what is your feeling on PNS is it a good initiative or not?

**[PARTICIPANT]**: notifying them is very important and a good initiative

**[INTERVIEWER]**: what will they benefit from notification? How will that help them?

**[PARTICIPANT]**: if we notify them they will get help and its very important

**[INTERVIEWER]**: how will that it be of help to them?

**[PARTICIPANT]**: as in how will that be of help to them?

**[INTERVIEWER]**: yes, when we call that, what will be our main objective?

**[PARTICIPANT]**: our main aim is to counsel them that sex between 2 men is...... I don't even know what to say

**[INTERVIEWER]**: don't worry you haven't done any mistake but I would just want to bring you to perspective, for example we call your partners, our main objective will be for them to be tested and to know their status. So the most important thing is they will have know their HIV status because they might be in danger of infection because of this one person who is infected. So when you come here your partner will benefit from testing and knowing their status

**[PARTICIPANT]**: true

**[INTERVIEWER]**: and after she knows her status the counselor would suggest she starts medication though we are different there are that will agree and those that wont agree and would want to think about and there are those that will totally refuse and take a long time but we would have achieved our objective and that she has been tested and she knows her status and the counselor would have done his counseling and told her on the important things to do and if she will not use medication then she protects herself from various things and like we always say people are different some people take along time to accept because they come they are talked to them they disappear for months then come back again until they accept. We always say the most important thing is acceptance, once someone accepts themselves then its easy but before someone accepts themselves then its not easy. Their are those who talk to the counselor and accept everything but will never come back again. That person never accepted herself because she was given conditions but when she accepts herself she will use the medication as prescribed. So the importance of PNS is the partners are tested and get to know their status so that they know what to do after that

**[PARTICIPANT]**: ok

**[INTERVIEWER]**: and are their any challenges that in using PNS on MSMs? Are their any challenges in following up on their partners?

**[PARTICIPANT]**: I don't know

**[INTERVIEWER]**: you don't know?

**[PARTICIPANT]**: yes maybe you explain to me

**[INTERVIEWER]**: maybe I explain to you?

**[PARTICIPANT]**: yes you expound on it

**[INTERVIEWER]**: like I said people are different and maybe on your side you haven't had any challenges in regards to PNs following up on your partners, some will tell you that their relationship has changed, and maybe he suspected one of the partners. For example I only have one partner and have been called to go to hospital for testing and that am I danger of being infected and I know I only have one partner of course I will know its that one partner

**[PARTICIPANT]**: yes you will know

**[INTERVIEWER]**: yes because I will be certain its that one person and suspecting you will be easy and that will give rise to a lot of things. Let me not talk about men a lot, maybe am the one who has put him under a risk of infection and I am violent I a might go and start telling people that you have infected me

**[PARTICIPANT]**: there are people who are like that

**[INTERVIEWER]**: that alone will harm you psychologically because she would have told people about your status

**[PARTICIPANT]**: she would have ruined your mind

**[INTERVIEWER]**: so somethings may happen to some people and to some it might not

**[PARTICIPANT]**: ok

**[INTERVIEWER]**: so like we discussed earlier there are different ways of notifying partners and I will remind you on the same we will start with the one that was infected first we call him index patient and then there is the study participant, the peer mobiliser and the counselor. Is that ok

**[PARTICIPANT]**: yes

**[INTERVIEWER]**: so the first way is the counselor to communicate with the partner of the infected client they she will be invited to come for testing. The counselor will call the partner of the infected client to come and know their status. The second method is the peer mobilizer will give you an oral test kit to test yourself and the third method is the peer mobilizer will go to your neighborhood you told him your partner could be found when he goes at a certain time he will find him and you can also arrange with the mobilizer if the partners shows up you will inform them. So the peer mobilizer will go there with the oral test kits so when he goes their he will carry a lot of oral test kits and now a days people are tested anywhere so he will come like he has just come to offer testing services and he will say he hasn't come with the kits that use blood but the oral testing kits and he will explain how the kits are used and people should go home and test themselves

**[PARTICIPANT]**: it will be confidential

**[INTERVIEWER]**: and after that if there is anything come to the hospital for confirmation but his main aim would be the one person that you have informed them that is there but he would go direct to that person, he will give the other people and make sure that that one person has also gotten a test kit and by that he would have achieved his goal

**[PARTICIPANT]**: I have understood you very well

**[INTERVIEWER]**: but he cant go direct because that person will suspect him and ask why have you just come to me?

**[PARTICIPANT]**: or what is the problem

**[INTERVIEWER]**: yes but if does it generally he wont suspect what is happening

**[PARTICIPANT]**: I have understood you

**[INTERVIEWER]**: the fourth way is by the counselor asking the counselor to invite her partner, this can happen when you and your partner come and then the counselor to assist you in disclosing to your partner you get tested like it's the first time so that you get tested together and get to know each others status without having any quarrels

**[PARTICIPANT]**: I think that is the best way

**[INTERVIEWER]**: the other way is through the peer mobilizer can help the client to invite the partner if possible the mobilizer accompanies you to go and talk to your partner and I think that method is good for you. So the methods are many and you can choose which one suits you best. Is that ok?

**[PARTICIPANT]**: yes

**[INTERVIEWER]**: and the last method is the client will give the partners the oral testing kit that is we give you the kit and you go give it to your partner so there are different methods and there is a possibility that you will use different methods with different partners. Its not a must to use one method on all the partners because the partners are different and you know how they are. There is that one whom you know if you give her the gadget she wont have a problem and another one who you know you have to accompany them. You can use different methods its not a must to use one method on all of them

**[PARTICIPANT]**: on all of them

**[INTERVIEWER]**: there is the one that you will have to inform peer mobilizers to come and test them in the neighborhood so that they know how to handle it. So there are different ways and they can work differently in different partners. Is that ok?

**[PARTICIPANT]**: yes

**[INTERVIEWER]**: so according to you which is the best method you can use

**[PARTICIPANT]**: which method did you mention and I told you it's the best way?

**[INTERVIEWER]**: the one that involves peer mobilizers going to your neighborhood?

**[PARTICIPANT]**: no

**[INTERVIEWER]**: the one that involves us giving you the test kit?

**[PARTICIPANT]**: yes

**[INTERVIEWER]**: ok that's fine. Is their any other method that you feel might be ok with you?

**[PARTICIPANT]**: even the method that involves going to the neighborhood isn't bad

**[INTERVIEWER]**: that is also good?

**[PARTICIPANT]**: yes

**[INTERVIEWER]**: so what time do you feel should be given in order for partners to be notified? Do you feel that we should talk about it their and then or should be given time to come back again?

**[PARTICIPANT]**: should be given time to come back

**[INTERVIEWER]**:why should they be given time?

**[PARTICIPANT]**: first they have to be contacted and then also give them time see how they will react to the situation. Because you cannot just go tell them lets go to the hospital tomorrow, you have to read their mind first.

**[INTERVIEWER]**: but this is someone you know?

**[PARTICIPANT]**: yes its someone you know but we are different you will have to first read his mind, bring him close because its not an easy thing and not everyone can accept easily. Someone might listen to you, take medication but when it comes to swallowing the medication then she doesn't take them and when you ask them "have you taken the medication?" she tells you she has taken them and in essence she hasn't. you have to bring them close and counsel them so its not something that should be done hurriedly its something that should be done step by step

**[INTERVIEWER]**: its ok I have understood you. Initially you had chosen that the counselor contacts your partners through the phone

**[PARTICIPANT]**: yes

**[INTERVIEWER]**: were it you who was contacting your partner what would you have told them to convince them to come to hospital? What words would you want to be used to entice her to come to hospital

**[PARTICIPANT]**: you can call her and....... (laughing)

**[INTERVIEWER]**: (giggling) we always say their has to be trust and that they should listen to you

**[PARTICIPANT]**: yes

**[INTERVIEWER]**: anyway we have reached the end of our discussion maybe is their anything that you would want to add or opinions that you would want to suggest about PNS on MSMs, Bisexuals or transgenders like we talked about?

**[PARTICIPANT]**: I would love if they are contacted so that they get medication and its also important for them to know their status

**[INTERVIEWER]**: ok so we a have reached the end of our discussion thank you for you time and thank you for coming today and today marks the end of our research

**[PARTICIPANT]**: ok

**[INTERVIEWER]**: but if there is anything we will contact you but it doesn't mean that our services have stopped, no our services will still go on ok

**[PARTICIPANT]**: yes

**[INTERVIEWER]**: you are most welcomed

**[PARTICIPANT]**: thank you
