# Supplementary material for: Peer-assisted HIV partner notification services to strengthen index partner testing for newly diagnosed men who have sex with men in coastal Kenya
Source: PLoS One. 2025 Oct 7;20(10):e0333707. doi: 10.1371/journal.pone.0333707 (PMC12503256; doi:10.1371/journal.pone.0333707)
Supplement: S3 Appendix — (ZIP) [file pone.0333707.s003.zip › Deidentified IDI Transcript_1556.docx]

**Participant characteristics:**

Age: 25-29

Sexuality: Bisexual

Education level: Secondary

Days between enrollment and IDI: 49 days

Mobilization strategy: PNS

Final PNS Strategy: N/A

**Partners identified: 1**

**[INTERVIEWER]**: so welcome to our discussion like I said we will invite you after one month and today we meet again on [DATE] and our discussion is taking place at [HOSPITAL_D] and if you remember the code that we gave you was 1556

**[PARTICIPANT]**: 1556

**[INTERVIEWER]**: yes that is your code. So like I said we would like to know more about partner notification better known as PNS that is after one has been tested and found to be positive

**[PARTICIPANT]**: ok

**[INTERVIEWER]**: we are saying PNS or partner notification is an initiative concept of following up partners after a person is tested and found to be positive. The person tested and found to be positive we call him an index client and PNS is voluntary. It is done by Health care workers, counselors or anyone you find at the health facility and we have different ways of conducting PNS, one way is through the health care explaining to you about PNS and you go tell your partner to come and get tested or by the counselor helping you notify your partners to come for testing and this is done by you giving the counselor phone contacts in order to notify them but he will not give any information about you

**[PARTICIPANT]**: wait I have a question

**[INTERVIEWER]**: ok ask me

**[PARTICIPANT]**: you have said PNS and one of the ways of following up is by giving phone contacts of the partners without them knowing that I am involved?

**[INTERVIEWER]**: yes so we are saying PNS has been on going but we still don't know how it can be done effectively on MSMs or bisexuals. Bisexuals are persons who have sex with both male and female and there are transgenders too

**[PARTICIPANT]**: yes

**[INTERVIEWER]**: so we are saying we don't have a clear understanding on how PNS can be effected on them and that's the main aim of this research and that we want to hear it from the horses mouth

**[PARTICIPANT]**: yes

**[INTERVIEWER]**: the person who is directly involved can tell us how best it can be done so that's the main aim of this research

**[PARTICIPANT]**: so that's the main aim?

**[INTERVIEWER]**: yes so how have you been since the last time you were tested?

**[PARTICIPANT]**: to say the truth I feel my body has changed much, when you look at me you can see the difference and I feel am doing fine. The best thing that happened is I knew my status but had I not known I wouldn't have gotten help

**[INTERVIEWER]**: have you gotten any challenges since you started your medication?

**[PARTICIPANT]**: I haven't had many challenges

**[INTERVIEWER]**: maybe what are some of the challenges that you might have faced?

**[PARTICIPANT]**: one you don't get sleep after taking the medication, two they make me dream a lot because I never used to dream so much but now a days I dream so much

**[INTERVIEWER]**: so what kind of dreams do you experience?

**[PARTICIPANT]**: surprisingly I don't have bad dreams, I dream of success or am somewhere not expected. There is a day I dreamt having my own car but when I woke up I didn't have anything

**[INTERVIEWER]**: (laughing)

**[PARTICIPANT]**: that is what surprised me and I knew its because of the medication and they also make you very alert

**[INTERVIEWER]**: so they make you feel very alert

**[PARTICIPANT]**: yes you become very alert you know am a driver so they make me very alert and by the way I set my time for taking the medication 10 at night because at that time I have to be home but I don't get sleep that is what surprises me because lets say I have chewed khat I will not get my sleep but in the morning I will feel sleepy so its like tis medication takes away my sleep coz I don't sleep at night and In the morning I also don't feel sleepy. It makes me sleep for only three hours so what exactly is that I would love you to answer that question

**[INTERVIEWER]**: people are different and they get different experiences while taking this medication. Some say they don't feel anything while taking these drugs

**[PARTICIPANT]**: and I also eat a lot if their was food here I would eat very much and I wouldn't lie to you I chew khat not every day but I can say only over the weekends and when I chew khat I never used to eat but now I eat so much, I might be chewing khat with my friends and just spit it out and eat

**[INTERVIEWER]**: wow

**[PARTICIPANT]**: yes am serious

**[INTERVIEWER]**: so is that something positive to you?

**[PARTICIPANT]**: yes it is because I don't even know how many times I eat in a day, in the morning I eat half a loaf of bread and tea, then I went to my mothers place she had cooked chips and I ate them after that I ate 2 chapatis and beans and after I leave this place I will be feel hungry so you tell me is that normal?

**[INTERVIEWER]**: (laughing)

**[PARTICIPANT]**: and I don't have money

**[INTERVIEWER]**: (laughing)

**[PARTICIPANT]**: cant you see am starting to have a pot belly

**[INTERVIEWER]**: (laughing) lets get back to our agenda

**[PARTICIPANT]**: but you will answer me that question later

**[INTERVIEWER]**: no problem, so what prompted you to test

**[PARTICIPANT]**: that's a very good question. Well it depends with the health care worker that you come across. He will not just come and tell me get tested. It took me a while to know that he was testing. He caught my attention and explained the importance of testing and also that he took his time to roam around in order to test people.

**[INTERVIEWER]**: what exactly did he tell you that made you go and check on your status?

**[PARTICIPANT]**: he told me that even if am found to be positive that is not the end of life and that if I know my status then it will be good, and I thank him so much because even after I found out I was positive It never affected me in anyway because of the counseling and courage he gave me, and am ok now you cant even know if I am infected

**[INTERVIEWER]**: you cant know

**[PARTICIPANT]**: in fact let me tell you I don't have stress at all

**[INTERVIEWER]**: that is very good and it means that you have accepted your status

**[PARTICIPANT]**: I don't have any stress

**[INTERVIEWER]**: we say that is positive leaving

**[PARTICIPANT]**: I will be coming after every three months maybe I undergo other forms of stress but I assume that I don't have HIV. In fact I haven't been hospitalized even with malaria, I don't have stress at all and there is a question I would like to ask you my sister

**[INTERVIEWER]**: no problem

**[PARTICIPANT]**: I would like to ask you one question. This medication apart from preventing or reducing the strength of HIV does it also change your thinking?

**[INTERVIEWER]**: how?

**[PARTICIPANT]**: as in they really reduce stress in my mind

**[INTERVIEWER]**: I don't think it has any relation.....

**[PARTICIPANT]**: and why (...)

**[INTERVIEWER]**: like I said people are different some people when they turn positive will undergo a lot of stress

**[PARTICIPANT]**: some even die

**[INTERVIEWER]**: yes and its not like this disease is what killed him but because of stress so we have reached a point where we do want to hear that someone has died because of HIV maybe something else because why should you die because of HIV and there are ways you can manage yourself? If you start medication early you will be ok just like you said earlier. Someone cant even know that you have been infected with HIV

**[PARTICIPANT]**: it is very hard

**[INTERVIEWER]**: very hard?

**[PARTICIPANT]**: And in fact let me tell you if you don't have that stress that you have HIV, you can even be afraid of having sex. Imagine telling someone that am HIV positive will he believe you?

**[INTERVIEWER]**: because they expect one to be very sick

**[PARTICIPANT]**: yes

**[INTERVIEWER]**: so we are trying to prevent that it doesn't reach at that point that's why we tell people to start medication early so that your health doesn't deteriorate because if it reaches that point then there is a possibility that one will know. You might be brought to hospital on a stretcher and the one who has brought you will definitely know what is going on

**[PARTICIPANT]**: its obvious they will know

**[INTERVIEWER]**: obviously they will know

**[PARTICIPANT]**: even if am taken to hospital because of malaria, because of my health the doctor wouldn't even think of testing me for HIV he will just test me for malaria. He will just assume which is very bad and that is what kills many people even on the road. Assuming is very bad that that vehicle is far away and you can overtake that's when you kill people. Assuming is a very bad thing

**[INTERVIEWER]**: did you use to test every now and then or when was the last time you tested?

**[PARTICIPANT]**: I wasn't afraid of testing I used to test after every 3 months but before I met the counselors in the field I had stayed for 6 months before testing

**[INTERVIEWER]**: but you used to test every now and then

**[PARTICIPANT]**: in fact let me tell you I had the courage to test because I was used to testing

**[INTERVIEWER]**: so how will we encourage MSMs to come for testing

**[PARTICIPANT]**: the main challenge is my phone if I had a permanent phone then all will be well

**[INTERVIEWER]**: its ok we are not talking about lovers we are talking about methods we can use to encourage MSMs to come for testing

**[PARTICIPANT]**: you are asking what are some of the ways we can use to encourage MSMs to come for testing?

**[INTERVIEWER]**: yes

**[PARTICIPANT]**: explain to them the importance of testing and what are the benefits and the counselors are supposed to encourage them on the importance of testing and knowing your status. They should tell them the disadvantages and advantages of testing

**[INTERVIEWER]**: apart from testing is their any other way that can help encourage MSMs to come for testing?

**[PARTICIPANT]**: kindly ask the question again

**[INTERVIEWER]**: ok apart from testing is their any other way that can help encourage MSMs to come for testing? Is their any other thing that can give them moral to come for testing?

**[PARTICIPANT]**: ok not very men have transport to come to this place and most don't know even if their if provision for transport to this place and as the client I will never know unless you tell me. You have the answers

**[INTERVIEWER]**: so maybe if I may clarify on the issue of transport, we are giving transport because it is a study so we are required to pay anyone who participates in this study but if its not a study then no transport is given you just come voluntarily to get the services so the transport is issued because of this study

**[PARTICIPANT]**: exactly most people say they wish they would have that transport to come to this place but because they are far they say they only have 200 shillings if I use it to go there what will I eat?

**[INTERVIEWER]**: and that is it

**[PARTICIPANT]**: yes that is it, another thing you have to have money to eat and if you take that medication without eating you feel like vomiting and I was never told the effects of this medication when I started but now know the side effects. One don't take the medication when you are hungry... yes I have tried that I wanted to know the side effects and if you take one tablet and put it in an open place it cracks like a flower. When you get a new thing you should first get to know the effects

**[INTERVIEWER]**: yes so that you get to know what you are using

**[PARTICIPANT]**: yes

**[INTERVIEWER]**: ok so have you told anyone about your status?

**[PARTICIPANT]**: I haven't told anyone

**[INTERVIEWER]**: is their anyone you would wish to tell but you don't know how to tell them?

**[PARTICIPANT]**: no one

**[INTERVIEWER]**: ok we are talking about follow up on your sexual partners so have their been any changes with the sexual partners that you mentioned last time?

**[PARTICIPANT]**: the biggest changes are brought through my phone because most of the time its not on so you never know maybe they have looked for me or not and since the last time I was here my phone was stolen and I lost all contacts and the line that I renewed has very little contacts like 20 contacts so after I get a phone everything will be ok

**[INTERVIEWER]**: so their hasn't been much communication because your phone had problems?

**[PARTICIPANT]**: yes

**[INTERVIEWER]**: so like we said earlier partner notification is normal and we are saying that the follow up is on partners who you have been with for the last one year that is 12 months and at first you talked about different partners so what made you open up and talk about them?

**[PARTICIPANT]**: I haven't understood that question

**[INTERVIEWER]**: what made you open up and talk about your partners?

**[PARTICIPANT]**: as in what made me talk about them?

**[INTERVIEWER]**: the day you were tested and found to be positive we talked about your partners

**[PARTICIPANT]**: yes

**[INTERVIEWER]**: and you gave me information about them?

**[PARTICIPANT]**: yes

**[INTERVIEWER]**: what made you talk about them?

**[PARTICIPANT]**: ok I also wanted them to know their status because its not good knowing that you were with someone and you are affected not knowing that you might have infected her. I will not be ok with out them knowing their status and that's why I would come to you so that you communicate with them secretly

**[INTERVIEWER]**: so the most important thing is them to be tested and to know their status

**[PARTICIPANT]**: in fact if I get one it will be ok. So you will device a way that they can reach here

**[INTERVIEWER]**: the most important thing is for them to be tested and know their status so that they can get help

**[PARTICIPANT]**: yes and if they are not infected then well and good

**[INTERVIEWER]**: then they will be able to protect themselves

**[PARTICIPANT]**: I also need to protect myself

**[INTERVIEWER]**: yes remember there is reinfection

**[PARTICIPANT]**: and reinfection is very bad coz you don't know the type of virus you are being infected with

**[INTERVIEWER]**: very true

**[PARTICIPANT]**: the reinfection is very bad

**[INTERVIEWER]**: you have a very good point most people after being found positive think they can infect other people and nothing will happen to them

**[PARTICIPANT]**: let me tell you I passed very well in biology

**[INTERVIEWER]**: (laughing)

**[PARTICIPANT]**: and business studies also mathematics

**[INTERVIEWER]**: ok

**[PARTICIPANT]**: I can just say that I lacked school fees but I wanted to study accounts and would have loved to work in a bank

**[INTERVIEWER]**: last time you told us about 2 partners that you were with in the last one year and also contacts on how to follow them up. So is it because you partners were few and you could remember them or there were other reasons?

**[PARTICIPANT]**: I felt happy talking about them because they are people the we respect each other and also we have no miss understanding

**[INTERVIEWER]**: so apart from the 2 that we talked about are their others that you feel we should talk about?

**[PARTICIPANT]**: none

**[INTERVIEWER]**: do you feel PNS is important to MSMs and do you feel it can help them know their status? Or do you have any opinion about MSMs?

**[PARTICIPANT]**: kindly ask the question again

**[INTERVIEWER]**: we talked about PNS is their any other way that we can use to notify your partners?

**[PARTICIPANT]**: the best way is contact through phone

**[INTERVIEWER]**: ok and what is your feeling about PNS?

**[PARTICIPANT]**: It is very important because they would also get assistance

**[INTERVIEWER]**: like I said we don't have a clear understanding how we can tackle PNS amongst MSMs, bisexuals or Transgenders that's the main objective of this research. How do you feel PNS should be tackled amongst MSMs?

**[PARTICIPANT]**: come again

**[INTERVIEWER]**: how will PNS work amongst MSMs, Bisexuals and Transgenders?

**[PARTICIPANT]**: it will reduce infections because like I have passed through counseling that means I can not have sex without a condom because I know my status and I know there is reinfection and its worse than infection and following them up also reduces infection for example everyone knows their status do you know HIV infection in Kenya will end? Because everyone would have known their status and that will end HIV infection

**[INTERVIEWER]**: I am happy with your opinion and do feel that there are any challenges that we might face

**[PARTICIPANT]**: yes there are challenges

**[INTERVIEWER]**: ok

**[PARTICIPANT]**: the field officers or even you it will depend on what you tell me to entice me because not all men who have sex with men can be identified just by looking at them many do it but you cant even say that they do it but through your sweet words I will give you all the attention and listen to you. Etiquette and general appearance is very important, how you look like and how you talk to me is very important.

**[INTERVIEWER]**: we said there are different ways of making PNS successful or different ways of following up on your partners I want to remind you those methods first we have the index patient, second the study participant, third is the peer mobilizer, and fourth is the counselor which is me. So the first method is me the counselor contacting your partner and invite her to come for testing that is after you give me her contacts, second the peer mobilizer will give the study participant a test kit just the same way you were given and the third way is by talking to the community mobilizer like you said your phone has challenges you can talk to the community mobilizer and tell them that the guy normally hangs around a certain neighborhood and a certain time or you can even arrange with the peer mobilizer that you will be at a certain place at a certain time so that he can come at that time and even if he doesn't know him personally but since you would have talked he will just come at that place so he will come with many of the oral testing kits and then explain to he group on the importance of testing just the same way people get tested out there. He will explain that the test kits can be used individually and uses oral fluids and then he explains to them how they are used and the issue the kits to every one that is there. So who ever he will be targeting will be there and will get the kit

**[PARTICIPANT]**: I have one question so this test kit you swab in your mouth and it gives you results and there is no need to use blood anymore? Does saliva have infection?

**[INTERVIEWER]**: the amount of virus in saliva is very minimal its not easy to be infected and for someone to be infected through saliva they ought to have swallowed 5 litres of saliva

**[PARTICIPANT]**: come again

**[INTERVIEWER]**: for someone to be infected through saliva they ought to have swallowed 5 litres of saliva of which that is impossible. So do the oral test kits test saliva? They don't test saliva and if they tested saliva you would have been told to spit on the reagent but no one tells you to do that but you swab the on the gums because we say there are traces of the virus in the gums and that is what we test and not saliva

**[PARTICIPANT]**: you have told me a very important thing and let me ask you for example I have a girlfriend and we are romancing and I have cut on my mouth and am bleeding without knowing am bleeding and maybe she has brushed her teeth so well to come and kiss me and maybe she has ulcerations that we we can infect one another

**[INTERVIEWER]**: yes

**[PARTICIPANT]**: and that is one of the importance of coming here

**[INTERVIEWER]**: yes to learn so the fourth way is through the counselor informing you to invite your partner to come for testing and the fifth way is through the peer mobilizer to assist you in inviting your partner to come for testing and another way is through the client giving his partner the oral self testing kit that is me giving you the kit and you to go and give it to your partner so we have talked of different ways so do you remember the first one that we talked about?

**[PARTICIPANT]**: use the counselor to get to my partner. Inform him where my partner stays and then he goes to give them the oral self test kits so she gets to know her status

**[INTERVIEWER]**: and among the methods we have talked about which one do you think is best?

**[PARTICIPANT]**: the best method is giving her mobile number to the counselor to make follow up because I might tell you he lives in [LOCATION_X] and you carry your kits everyday but you wont find him there. So why don't I just give you her number so that you can make follow up?

**[INTERVIEWER]**: ok so what time should someone be given before follow up is made? Is it the same day or we should give you some bit of time?

**[PARTICIPANT]**: I haven't understood you

**[INTERVIEWER]**: when we talk about partner follow up, what is the time should be taken before follow up is made?

**[PARTICIPANT]**: for example you have tested me today then we start talking about my partners? No that will not be ok. I think the best thing will be to start telling me the importance of medication I mean you approach it in a way that the clients even starts looking for you. For example I fall in love with you today I cant seduce you today and go and sleep with you today you will have a lot of thoughts and it will disturb you so much and might start thinking like either am infected and want to infect you but if I seduce you and give you time and I only text you goodnight and good morning then you will start looking for me true or false?

**[INTERVIEWER]**: (laughing) ok I have understood you

**[PARTICIPANT]**: (laughing)

**[INTERVIEWER]**: so if we talk bout the partners that we talked about first you suggested that the best way to contact them is by the counselor having their contacts so that he can call them

**[PARTICIPANT]**: yes I will look for their numbers and give it to the counselor

**[INTERVIEWER]**: so if I may ask you, if you were the counselor what words will you tell the partner so that he can come for testing?

**[PARTICIPANT]**: for example you have given me that job the first thing is you don't call them text them stating that their will be a free medical camp at the general hospital and put it in a general way so that it looks like its an offer then she will say this that this is my lucky day to get free medical attention and through that you would have got them to come and test. And she will come and meet other people too and she might even state that she was contacted to come for the free medical camp

**[INTERVIEWER]**: that is an excellent idea and thank you for that you have excellent ideas

**[PARTICIPANT]**: because for example you have a friend but I don't want your friend because I want you. You know I cant come and take your number directly from you obviously you will refuse because you are with your friend and you may think that your friend will see you as being cheap but deep down you want to give me the number but you are afraid because of your friend so you know what you can do? You will pretend you don't have airtime and I will ask your friend to give me her phone to call you and that's how I will take your number

**[INTERVIEWER]**: (laughing) you have very funny examples

**[PARTICIPANT]**: but if I give you my partners number and you call her, it will ring in her mind that I am the one who gave you her number you know why? It will ring in him that we are talking about MSMs and he ask himself has my boyfriend been at the hospital? So you need to work in a way that he wont think of me first make it in a way that he will feel it involves everyone else in the country and mention to him that once he gets here his transport will be catered for without a problem. And he will also say that if my partner gets to hear about this he will also get help. When he turns positive he will also start using same tricks am using now (laughing)

**[INTERVIEWER]**: (laughing) ok

**[PARTICIPANT]**: have you gotten my concept?

**[INTERVIEWER]**: so we are coming to the end of our discussion I don't know if you have anything you would like us to talk about in regards to partner notification?

**[PARTICIPANT]**: ok you are asking if I have other opinions

**[INTERVIEWER]**: yes

**[PARTICIPANT]**: ok I cant tell you that follow up through phone call isn't appropriately but the method I have given you will work effectively

**[INTERVIEWER]**: ok

**[PARTICIPANT]**: and while contacting them make sure you don't use a line that will register a name on top it should come with the name of the organization like [RESEARCH_INSTITUTION]

**[INTERVIEWER]**:ok (laughing)

**[PARTICIPANT]**: because if it just appears without a name they might say these are just conmen but if the name [RESEARCH_INSTITUTION] appears then they will know it's a big organization known country wide

**[INTERVIEWER]**: thank you so much for your opinions

**[PARTICIPANT]**: is their any job opportunity the tuk tuk job we are doing is so stressful

**[INTERVIEWER]**: ok we will explain to you later what we do so that you maybe assisted

**[PARTICIPANT]**: if you want a driver am here I know how to drive vehicles and am readily available

**[INTERVIEWER]**: so thank you for your time we appreciate so much and we have come to the end of our discussion so thank you so much and this also marks the end of our research incase there is anything else we will inform you we have your contacts and it doesn't mean this is the end of you getting services no services are still their

**[PARTICIPANT]**: ok you know there are many ways of killing a rat and if your ways of killing a rat doesn't work you can consult me for ideas

**[INTERVIEWER]**: (laughing) ok thank you

**[PARTICIPANT]**: so we are the rats (laughing)

**[INTERVIEWER]**: ok thank you
