# Supplementary material for: Peer-assisted HIV partner notification services to strengthen index partner testing for newly diagnosed men who have sex with men in coastal Kenya
Source: PLoS One. 2025 Oct 7;20(10):e0333707. doi: 10.1371/journal.pone.0333707 (PMC12503256; doi:10.1371/journal.pone.0333707)
Supplement: S3 Appendix — (ZIP) [file pone.0333707.s003.zip › Deidentified IDI Transcript_1566.docx]

**Participant characteristics:**

Age: 25-29

Sexuality: Bisexual

Education level: Secondary

Days between enrollment and IDI: 61 days

Mobilization strategy: OST

Final PNS Strategy: N/A

**Partners identified: 3**

**[INTERVIEWER]**: welcome to our discussion today is?.......

**[PARTICIPANT]**: [DATE]

**[INTERVIEWER]**: we had to do this discussion today because I heard that you wanted to travel and you wont be available next week

**[PARTICIPANT]**: yes there are a lot of issues I want to tackle

**[INTERVIEWER]**: so today is [DATE] and the interview is taking place at [HOSPITAL_D]. So like we discussed earlier we would like to know more about PNS which is a partner notification service after someone has been tested for HIV and found to be positive. This is to help prevent the spread of HIV and also to help one start medication early. So the person infected we refer to him as the index client and PNS is voluntary and if you feel you don't want to go on with PNS then you just stop so its not mandatory but its important so its upon you to make that decision.

**[PARTICIPANT]**: ok

**[INTERVIEWER]**: so there are different ways that PNS can be done one is through the help of a counselor to help you inform your partner or by you informing your partner so its still upon you to decide. If the counselor is to help you then you give the contacts of your partner and we call him but we hide information about you so she wont know its you who gave the contacts. So PNS is common and its happening every where you go for testing you have to be involved in PNS be it MSMs or any other type of sex. We still don't have a clear understanding how PNS can be done to these people that at a risk of HIV infection that is the MSMs, Bisexuals and Transgender so that's why we are conducting this research so as to know how we can go about PNS amongst this population

**[PARTICIPANT]**: ok

**[INTERVIEWER]**: so how have you been since the last time you knew your status?

**[PARTICIPANT]**: (short silence) I have seen any changes

**[INTERVIEWER]**: you haven't seen any changes?

**[PARTICIPANT]**: I haven't been sick

**[INTERVIEWER]**: kindly talk a bit louder so that we can capture it on the recorder

**[PARTICIPANT]**: I haven't been sick but the only thing that disturbs me is making the decision as in what disturbs me a lot is I think a lot

**[INTERVIEWER]**: you think a lot?

**[PARTICIPANT]**: yes and I haven't thought of anyone that I could share this with anyone

**[INTERVIEWER]**: when you say you haven't made a decision, what exactly haven't you decided?

**[PARTICIPANT]**: I tested positive and had to come here for medication so that's where am a bit challenged that is coming to take medication

**[INTERVIEWER]**: so you discussed and were told to start medication?

**[PARTICIPANT]**: yes

**[INTERVIEWER]**: did they open a file for you? And the tests done?

**[PARTICIPANT]**: I used to stay at [LOCATION_C] place but I was to shift to [LOCATION_D] so he told me to go to [LOCATION_D] [CLINIC_C] and I didn't go.

**[INTERVIEWER]**: so you haven't gone to [CLINIC_C]?

**[PARTICIPANT]**: no I haven’t

**[INTERVIEWER]**: so you haven't started your medication?

**[PARTICIPANT]**: I haven't started

**[INTERVIEWER]**: so what is making you not start your medication?

**[PARTICIPANT]**: (short silence)

**[INTERVIEWER]**: or what goes through your mind when you think of starting medication?

**[PARTICIPANT]**: the guy told me once you start the medication you have to take them daily so its something I haven't decided

**[INTERVIEWER]**: first HIV doesn't have a cure, researchers are still looking for a cure but up to now no cure has been found so the only option is taking medication and this medication has to be taken daily for the rest of your life and this medication is very important because when you get infected the virus multiplies in your body such that infecting someone else is very easy and you health will deteriorate drastically because you haven't started medication. People are different there are those who are tested and start medication immediately but some take a bit of time. But its good to know that when we take time to start medication the virus continues to multiply so as much as we would want to take time to start medication but it is important to start medication early for your own sake

**[PARTICIPANT]**: ok

**[INTERVIEWER]**: so we will talk about that later

**[PARTICIPANT]**: ok

**[INTERVIEWER]**: so when you were tested and found to be positive what made you test at that time?

**[PARTICIPANT]**: I just made a decision

**[INTERVIEWER]**: so you took a lot of courage that day?

**[PARTICIPANT]**: yes

**[INTERVIEWER]**: so it means that you don't test or take time to test?

**[PARTICIPANT]**: I have tested severally even by these counselors who move door to door like last year or last year but one

**[INTERVIEWER]**: ok so then you came and tested with the oral test kit?

**[PARTICIPANT]**: yes this year

**[INTERVIEWER]**: when you were given the oral test kit did you meet one of our peer mobilizers? An if you did what did he tell you?

**[PARTICIPANT]**: it was the first time for me to come across the oral test kit and I felt good because it was a way of me knowing my status when I was alone and it was a secret

**[INTERVIEWER]**: so you were excited to know how it will work?

**[PARTICIPANT]**: yes

**[INTERVIEWER]**: so that when you decided to take it and use it?

**[PARTICIPANT]**: yes

**[INTERVIEWER]**: did the mobilizer tell you anything that made you want to know your status?

**[PARTICIPANT]**: he told me about the oral kit and how it works and that I can use it at home without coming to hospital and if I have ever tested using it and I told him no I haven't so that when I decided to take it

**[INTERVIEWER]**: and how do you think we can encourage MSMs to test for HIV? Because many don't like to be tested. You had stayed for 2 years without testing isn't it? What can we do to entice them to come for testing?

**[PARTICIPANT]**: by talking to them

**[INTERVIEWER]**: what should we tell them?

**[PARTICIPANT]**: importance of testing

**[INTERVIEWER]**: ok

**[PARTICIPANT]**: like anyone who has sex should know their status

**[INTERVIEWER]**: ok I have understood you so you have told me you haven't started medication isn't it?

**[PARTICIPANT]**: yes

**[INTERVIEWER]**: you met with your counselor

**[PARTICIPANT]**: yes

**[INTERVIEWER]**: the words he told you, do you feel they helped you in any way? What can you say about the counseling you got after knowing your status?

**[PARTICIPANT]**: I got good counseling

**[INTERVIEWER]**: ok the counseling was good but you haven't started medication? You are making us worry so much. Is their anything more that we should do?

**[PARTICIPANT]**: no you guys are ok

**[INTERVIEWER]**: so its up to you now?

**[PARTICIPANT]**: yes just give me time (giggling)

**[INTERVIEWER]**: so we will talk about the partners that you talked about to the counselor and because I wasn't their would you mind sharing how many partners you talked about?

**[PARTICIPANT]**: I talked about 4 partners

**[INTERVIEWER]**: about 4?

**[PARTICIPANT]**: yes

**[INTERVIEWER]**: among the four how many were male and how many were female?

**[PARTICIPANT]**: 2 male and 3 female I think they were 5 and not 4

**[INTERVIEWER]**: so I would like us to talk about the partners you talked about the last time. So do you feel that PNS should be done immediately or should wait a little bit

**[PARTICIPANT]**: as in to call me and ask me about this issue?

**[INTERVIEWER]**: yes to talk about your partners. Did you talk about it the same day or you took time

**[PARTICIPANT]**: we talked about it the same day

**[INTERVIEWER]**: the same day?

**[PARTICIPANT]**: yes

**[INTERVIEWER]**: what is your feeling about that? You have known your status the same day and the same day you are talking about your partners do you feel like it was the right time to talk about your partners? Or do you feel you should have been given al little bit of time and then come back and talk about your partners?

**[PARTICIPANT]**: it is good and also it is not good but because I indulged in sexual intercourse the one that I did it with definitely would come to the hospital (laughing) and that's why he asked me how many people I indulge in sexual intercourse with

**[INTERVIEWER]**: so why do you say its bad on the other hand?

**[PARTICIPANT]**: bad because he said he will try to find a way of telling them without informing me so I think that is ok

**[INTERVIEWER]**: so that is ok? So without your partner knowing that you informed the hospital that will be ok?

**[PARTICIPANT]**: yes

**[INTERVIEWER]**: so did he tell you about any ways of informing your partners? Did you talk about any ways apart from him calling?

**[PARTICIPANT]**: we talked about him calling and I left contacts but he also told me if I can convince them to come and take the test which I didn't like

**[INTERVIEWER]**: so you opted for him to look for them?

**[PARTICIPANT]**: yes

**[INTERVIEWER]**: and you gave information on how he could get them?

**[PARTICIPANT]**: yes

**[INTERVIEWER]**: and were you able to know if your partners were notified to come for testing? Might you be having any information about that?

**[PARTICIPANT]**: I told him about 1 and I haven't met the others since the last time we met

**[INTERVIEWER]**: and would you know if the 1 that you met was contacted?

**[PARTICIPANT]**: yes

**[INTERVIEWER]**: so you know that he was contacted?

**[PARTICIPANT]**: no I don't I thought you asked me if would like to know if he was contacted

**[INTERVIEWER]**: no I wanted to know if you know that he was contacted?

**[PARTICIPANT]**: no I don’t

**[INTERVIEWER]**: so in short he hasn't told you anything

**[PARTICIPANT]**: he hasn't told me anything

**[INTERVIEWER]**: so you have told me all your partners were contacted by the counselor? You gave him the contacts so that they can be contacted?

**[PARTICIPANT]**: yes

**[INTERVIEWER]**: and as we speak you don't have information whether they were contacted or not

**[PARTICIPANT]**: yes but I have only met 1

**[INTERVIEWER]**: but you don't know if he was contacted or not?

**[PARTICIPANT]**: yes he didn't tell me if he was contacted

**[INTERVIEWER]**: so the one that you have met with what is your relationship like? How is your relationship? Has it changed in any way?

**[PARTICIPANT]**: (short silence) I haven't seen any changes

**[INTERVIEWER]**: so there are no changes in your relationship?

**[PARTICIPANT]**: no

**[INTERVIEWER]**: so apart from those that you talked to the counselor about, is their anyone that you didn't mention that you would like us to mention today?

**[PARTICIPANT]**: no their isn't

**[INTERVIEWER]**: so we will talk about disclosure and if there is anyone that you informed about your status but you stated that you haven't told anyone about your status

**[PARTICIPANT]**: not yet

**[INTERVIEWER]**: its about a month since you last tested isn't it?

**[PARTICIPANT]**:yes

**[INTERVIEWER]**: so who do you think you can disclose your status to?

**[PARTICIPANT]**: my parents

**[INTERVIEWER]**: both your father and mother?

**[PARTICIPANT]**: no mother

**[INTERVIEWER]**: why would you tell your mother?

**[PARTICIPANT]**: because I stay close to my mother and my father is a bit far

**[INTERVIEWER]**: ok you live together in [CITY_A]?

**[PARTICIPANT]**: yes but I live independently

**[INTERVIEWER]**: and how do you think she will take it when you disclose your status to her?

**[PARTICIPANT]**: I cant know how she will react

**[INTERVIEWER]**: do have any closeness together and she understands you so how do you think she is going to take it?

**[PARTICIPANT]**: she will be mad at me

**[INTERVIEWER]**: she will be mad of you because you are infected or (sneezing) kindly elaborate farther

**[PARTICIPANT]**: she will be mad at me because I was reckless though am not that way but that is what she will think

**[INTERVIEWER]**: so up to now people perceive HIV in a different way out there that you have to be promiscuous to get HIV is that the case?

**[PARTICIPANT]**: its not like that because there are many ways to get it

**[INTERVIEWER]**: yes there are different ways of getting it

**[PARTICIPANT]**: but according to her she will see it that way that is how I know her

**[INTERVIEWER]**: so that is what has made you not tell anyone?

**[PARTICIPANT]**: yes I haven't told anyone but am thinking on how to tell her and I still don't know how to tell her. So we meet every Sunday but today I haven't met her she either visits me or I visit her because she stays in [NEIGHBORHOOD_D] so she can either come or I go and we talk but I haven't met her

**[INTERVIEWER]**: lets assume you are the parent and your child has been infected how would you want your child to tell you or prepare you?

**[PARTICIPANT]**: he should just tell me

**[INTERVIEWER]**: How in what way? You are the parent and your child has been tested and is found positive as a parent how would you want him to tell you? You told me you would want him to tell you?

**[PARTICIPANT]**: yes

**[INTERVIEWER]**: so what is the best time for him to tell you? I don't know if you understand me

**[PARTICIPANT]**: I understand you and this is something that just happened and I will definitely start medication but maybe I jus inform you as the counselor to talk to her

**[INTERVIEWER]**: so you need to take the initiative of bringing her here so that we can talk to her but the best thing is for you to start medication that is more important than someone knowing your status

**[PARTICIPANT]**: ok

**[INTERVIEWER]**: so the most important think is you to start medication, informing your mother will be done later but before we tell her you should make your choice about starting medication so that you can help yourself because many people have HIV and they still leave a normal life. When someone looks at you will they know that you are positive? But the more you stay without starting medication the sicker you become until your mother carries you to hospital and by then you wouldn't be in a position to make any decision and everyone will know

**[PARTICIPANT]**: that's true

**[INTERVIEWER]**: you didn't want everyone to know but she has known either way but because you are still healthy and you can tell whoever you want this is the best time to make choices but lets not wait until you cant help it

**[PARTICIPANT]**: ok

**[INTERVIEWER]**: so we will still go back to you disclosing tour partners we would like to know if their have been any changes or challenges after them being informed. But you have told me you haven't met them

**[PARTICIPANT]**: yes I only met one

**[INTERVIEWER]**: its only one and you haven't seen any changes?

**[PARTICIPANT]**: yes I don't know if he was called because I see him stronger now a days (laughing)

**[INTERVIEWER]**: but you never know

**[PARTICIPANT]**: you told me if I wanted to know if they were called

**[INTERVIEWER]**: my question was do you know if they were called?

**[PARTICIPANT]**: I don't know

**[INTERVIEWER]**: you know others will tell you they were called or if tell you they came and were tested

**[PARTICIPANT]**: no they haven't told me

**[INTERVIEWER]**: so the same way the counselor kept his confidentiality is the same way he kept his confidentiality about your partners so you wouldn't know who has come and who hasn't

**[PARTICIPANT]**: yes

**[INTERVIEWER]**: so if I go back a bit we are saying PNS has had a lot of challenges and specifically when someone has been tested and found to be positive and like the counselor did he would like for you to talk about the partners you were with in the last one year (sneezing) so how as it when you talked to the counselor how you explained to him about your partners

**[PARTICIPANT]**: (giggling)

**[INTERVIEWER]**: were you able to talk about the five partners? And what went through your mind when you were telling him?

**[PARTICIPANT]**: just told him how we did it

**[INTERVIEWER]**: how did you do it

**[PARTICIPANT]**: I don't know who did it

**[INTERVIEWER]**: you don't know who infected you?

**[PARTICIPANT]**: yes or who have I infected

**[INTERVIEWER]**: ok also who have you infected

**[PARTICIPANT]**: yes

**[INTERVIEWER]**: and how were you able to share with the counselor about your partners?

**[PARTICIPANT]**: told him how it happened and how often we met to do it

**[INTERVIEWER]**: so did you see any importance of them coming for testing?

**[PARTICIPANT]**: yes

**[INTERVIEWER]**: which one?

**[PARTICIPANT]**: so that they know their status

**[INTERVIEWER]**: ok there are times it's a bit difficult to talk about partners and was it easy for you to identify those partners? Because you have been with them and you know how to get them? Or are their others that you couldn't remember?

**[PARTICIPANT]**: there are others but they are my "EXE" partners

**[INTERVIEWER]**: they are your EXE partners?

**[PARTICIPANT]**: yes I can only remember the recent ones

**[INTERVIEWER]**: ooh they are recent partners?

**[PARTICIPANT]**: yes

**[INTERVIEWER]**: so the EXEs are they within the one year period or they are not?

**[PARTICIPANT]**: no they are not

**[INTERVIEWER]**: ooh you dated long ago?

**[PARTICIPANT]**: yes but I still remember them

**[INTERVIEWER]**: so you only gave contacts of your recent partners and no one else?

**[PARTICIPANT]**: yes and I don't want any other

**[INTERVIEWER]**: why and life has to go on? That shouldn't make you not live your life when you take the step of starting medication you will just see life moving on but its not that easy because we are different because some take time to accept but that shouldn't make you change focus in life

**[PARTICIPANT]**: ok

(short silence)

**[INTERVIEWER]**: so we will talk about PNS, so the counselor called your partners?

**[PARTICIPANT]**: yes

**[INTERVIEWER]**: like we said earlier PNS is something that is on going and its done to anyone who is infected with HIV

**[PARTICIPANT]**: yes

**[INTERVIEWER]**: so what is your take on PNS? Informing your partners, what can you say about that?

**[PARTICIPANT]**: it is ok only that no one should mention me

**[INTERVIEWER]**: so you would recommend it to be done to other people?

**[PARTICIPANT]**: yes only that the counselor calls to inform the partners and some people may call and ask where I got the number but you have to work in a smart way

**[INTERVIEWER]**: so as for now we still don't have and idea of how to do it amongst MSMs so what is your opinion if it was to be done amongst MSMs, Transgender or Bisexuals? When we start giving the services amongst that group?

**[PARTICIPANT]**: there is no problem

**[INTERVIEWER]**: their wont be any issues?

**[PARTICIPANT]**: their will be some issues

**[INTERVIEWER]**: ok what are some of the issues?

**[PARTICIPANT]**: same issues people understanding the situation as in you are asking me if we are to use the method (PNS) to inform them

**[INTERVIEWER]**: yes

**[PARTICIPANT]**: the problem will be them asking where the counselor got the number from as in that will be the main challenge same to the male and female partner

**[INTERVIEWER]**: so the challenges will be the same amongst straight and MSMs?

**[PARTICIPANT]**: yes

**[INTERVIEWER]**: the issues of where the counselor got the number from

**[PARTICIPANT]**: yes same challenges

**[INTERVIEWER]**: and are their any benefits of PNS?

**[PARTICIPANT]**: yes one when you give out the partners contacts without being mentioned

**[INTERVIEWER]**: ok

**[PARTICIPANT]**: and second the partner would have gotten help

**[INTERVIEWER]**: ok they would have gotten help?

**[PARTICIPANT]**: yes

**[INTERVIEWER]**: ok so how would they have been helped?

**[PARTICIPANT]**: by getting to know their status

**[INTERVIEWER]**: ok knowing their status

**[PARTICIPANT]**: yes

**[INTERVIEWER]**: so how will that have helped him?

**[PARTICIPANT]**: after that many other things will follow

**[INTERVIEWER]**: so what are these things that will follow?

**[PARTICIPANT]**: medication

**[INTERVIEWER]**: yes they will know their status and get medication

**[PARTICIPANT]**: yes

**[INTERVIEWER]**: there is a possibility that one will be tested and found to be ok?

**[PARTICIPANT]**: yes

**[INTERVIEWER]**: and how will they be helped

**[PARTICIPANT]**: they will be happy

**[INTERVIEWER]**: They will be happy so apart from that how will they be helped?

**[PARTICIPANT]**: should be told to use protection and get proper counseling

**[INTERVIEWER]**: so like we said earlier there are different ways of notifying our partners. There is the index client then there is the study participant, the peer mobilizer and then the counselor and I will talk about the different ways of notifying the partners so the first way which involves the counselor I can get in contact with your partner and invite him to come and get tested but without giving any information about you. The second way is through the peer mobilizer giving you the self test kit same as the one you used he can give you to go give your partner and the third method is through the peer mobilizer going to your partners neighborhood and give out the self testing kits but with the aim of giving it to your partner. You would have informed him of where he stays and then he will go there but he should go directly and give the kit to him because he will feel suspected so what I will do I will give the kits to many people including him. They will be informed that after testing they should come to hospital and know their status

**[PARTICIPANT]**: ok

**[INTERVIEWER]**: and the fourth way is by the counselor assisting the client to invite the partner for testing so we can invite your partner to come for testing so that she can know her status and the fifth way is the peer mobilizer can assist you to invite your client for testing

**[PARTICIPANT]**: ok

**[INTERVIEWER]**: he can accompany you to talk to your partner so that he can know his status and the sixth way is through you giving the client the self testing kit so that they can test themselves

**[PARTICIPANT]**: ok

**[INTERVIEWER]**: so we have talked about six ways which one do you think can work best?

**[PARTICIPANT]**: the one that involves peer mobilizers going to the neighborhood (laughing) I just point to the person

**[INTERVIEWER]**: so we go to the neighborhood at a certain time in order to get them?

**[PARTICIPANT]**: yes

**[INTERVIEWER]**: we go their and give out the kits?

**[PARTICIPANT]**: yes

**[INTERVIEWER]**: why do you feel that will work best?

**[PARTICIPANT]**: because they will think that it's a service being offered so they wouldn't know that the peer mobilizers have been sent. He wont suspect anything

**[INTERVIEWER]**: so this method will work best amongst your partners?

**[PARTICIPANT]**: yes

**[INTERVIEWER]**: so how do you feel going to the counselor and explaining to him that and see if he contacted some of your partners? So that if there are some he didn't get then follow up can be done

**[PARTICIPANT]**: yes

**[INTERVIEWER]**: so what is the best time to talk about PNS after someone has been infected? Is it the same time or they should come at a different time?

**[PARTICIPANT]**: it depends on the person

**[INTERVIEWER]**: the counselor or the client?

**[PARTICIPANT]**: the client

**[INTERVIEWER]**: the client

**[PARTICIPANT]**: yes

**[INTERVIEWER]**: I haven't understood you

**[PARTICIPANT]**: as in the client might be ready for PNS after testing

**[INTERVIEWER]**: so people are different there are those that would want to come later and there are those that would want it immediately

**[PARTICIPANT]**: yes

**[INTERVIEWER]**: ok there is this method that you had picked earlier that involved calling them via phone

**[PARTICIPANT]**: yes but he hadn't told me about these other methods

**[INTERVIEWER]**: ok he hadn't told you about these other methods?

**[PARTICIPANT]**: yes

**[INTERVIEWER]**: for example you were the counselor contacting the partners how would you explain to them or what words would you use?

**[PARTICIPANT]**: I don't know what I would say (laughing) that will be something new

**[INTERVIEWER]**: ok so we are coming to the end of our discussion is their anything that you would like to talk about in regards to PNS and MSMs, Bisexuals, and transgenders? Is their anything that you would like to add or talk about?

**[PARTICIPANT]**: I don't have any problems with them but it is important they get tested

**[INTERVIEWER]**: so if you don't have any additional opinions or suggestion, we have come to the end of out discussion and thank you for your time and for coming and taking time to participate in this discussion. Thank you so much and welcome

**[PARTICIPANT]**: thank you
