# Supplementary material for: Peer-assisted HIV partner notification services to strengthen index partner testing for newly diagnosed men who have sex with men in coastal Kenya
Source: PLoS One. 2025 Oct 7;20(10):e0333707. doi: 10.1371/journal.pone.0333707 (PMC12503256; doi:10.1371/journal.pone.0333707)
Supplement: S3 Appendix — (ZIP) [file pone.0333707.s003.zip › Deidentified IDI Transcript_1571.docx]

**Participant characteristics:**

Age: 25-29

Sexuality: Bisexual

Education level: Secondary

Days between enrollment and IDI: 23 days

Mobilization strategy: OST

Final PNS Strategy: HCP/PM

**Partners identified: 1**

**Introduction**

**[INTERVIEWER]:** so we will talk about the services or how someone can bring their sexual partners for testing after knowing there HIV status .There are different ways which we can help your sexual partner have this test, you ask your partner to come and get tested here together or you can ask the counsellor to call them and they come and get tested or our peer educators can come to your place and provide oral self test kits or we can give you the self test kit to take it to your sexual partner then come to the clinic for confirmation. So, we were trying to see if this partner notification services can help people whose partners have been infected with HIV can either be started on ARVs if infected too or prep if negative which can reduce their chances of being infected, have you understood?

**[PARTICIPANT]: Yes**

**[INTERVIEWER]**: so, you will explain to me in brief how you felt when you tested positive...

**[PARTICIPANT]**: To be honest I felt very sad and lost all hopes, I thought my life had come to an end

**[INTERVIEWER]**: So, when you were testing did you have any fears?

**[PARTICIPANT]**: Yes, I feared that my family would rejected and stigmatize me and I won't have friends anymore.

**[INTERVIEWER]**: What do you think is the main reason that made you test?

**[PARTICIPANT]**: The reason that made me test is that I wanted to travel abroad, and medical tests were a must.

**[INTERVIEWER]**: So, was that the first time you were testing?

**[PARTICIPANT]**: Yes, it was my first time

**[INTERVIEWER]**: Okay, so when you tested and found out your HIV positive. Was it the first time that you knew that you were positive?

**[PARTICIPANT]**: It was the first time, the first time

**[INTERVIEWER]:** Apart from travelling are there any other reasons that made you take that test

**[PARTICIPANT]:** no there is no other reason

**[INTERVIEWER]**: how did the peer mobiliser or peer educator approach or explain it to you?

**[PARTICIPANT]**: he explained to me the importance of knowing my HIV status and that it was a must for me to visit a health facility as there was this self test kit that someone can perform while at home which I thought it was a good idea.

**[INTERVIEWER]:** When the mobiliser was explaining to you about the oral self test, did he also talk about your risk behaviours and acute HIV infection and oral self-test?

**[PARTICIPANT]**: Yes, he told me about oral self-test.

**[INTERVIEWER]**: To what extent did you understand about HIV oral self-test kit?

**[PARTICIPANT]**: Yes, what I understood was I can test using my saliva while alone without anyone.

**[INTERVIEWER]:** when he was giving you the oral self test did he talk to you about early HIV infection AHI; did he give you any fliers or refer you to any online materials that had more information on oral self-tests

**[PARTICIPANT]**: No,

**[INTERVIEWER]:** When he come to you what was your experience with the mobiliser?

**[PARTICIPANT]**: it was fine

**[INTERVIEWER]**: how did you take it when he approached you?

**[PARTICIPANT]**: I took it as normal as I knew he cared about my health and that of the people I love

**[INTERVIEWER]**: how do you think we can motivate people who are have sex with fellow men(MSM) so that they can know their HIV status?

**[PARTICIPANT]**: I think through counselling and mobilization.

**[INTERVIEWER]**: when you tested for the first time what were your HIV results?

**[PARTICIPANT]**: My results were positive

**[INTERVIEWER]**: what was the experience when you found out that you are HIV positive?

**[PARTICIPANT]**: Then or now?

**[PARTICIPANT]:** my experience was that someone can test on there on using self test which I think is more accurate than the test we were using before which involved being pricked and getting of blood, which people fear, this oral self-test only involves swabbing which I find it very good.

**[INTERVIEWER]:** after learning your status did you immediately start treatment?

**[PARTICIPANT]**: No, I didn't

**[INTERVIEWER]**: Why didn't you?

**[PARTICIPANT]**: I still hadn't accepted my HIV status.

**[INTERVIEWER]**: so, what made you to finally start your treatment?

**[PARTICIPANT]**: my health started deteriorating, I didn't have any appetite so I decide to start medication.

**[INTERVIEWER]**: how did you feel when you started your treatment?

**[PARTICIPANT]**. I started feeling better, stronger and my appetite came back and I was told to take my medications on time because it's what keeps me alive.

**[INTERVIEWER]**: were you given any advice from the counsellor when you were starting the medications

**[PARTICIPANT]**: Yes, I was told to accept my status and follow the clinician's advice

**[INTERVIEWER]**: do you think this advice has helped you in way?

**[PARTICIPANT]**: Yes, it has.

**[INTERVIEWER]**: Now we will talk about PNS, how was this service introduced to you?

**[PARTICIPANT]**: this service was introduced to me when I wanted to disclose my status to my partners.

**[INTERVIEWER]:** what advice did you get on how to let your partner to know their status

**[PARTICIPANT]**: The advice I got was on usage of self tests

**[INTERVIEWER]:** Any other strategy apart from use of self tests

**[PARTICIPANT]**: Yes, through peer mobilisers

**[INTERVIEWER]**: apart from peer mobilisers

**[PARTICIPANT]**: Giving them the self-test kit.

**[INTERVIEWER]**: so how do you feel about the strategies on partner notification

**[PARTICIPANT]**: I think they are okay

**[INTERVIEWER]**: Which option did you choose to use to inform your sexual partners to go and know their HIV status?

**[PARTICIPANT]**: I chose using the mobiliser to face him and offer him the self

**[INTERVIEWER]:** how do you feel about this strategy now

**[PARTICIPANT]**: it's okay

**[INTERVIEWER]:** did all your partners get notified about knowing their HIV status?

**[PARTICIPANT]**: yes, they were

**[INTERVIEWER]:** how would react if your notified about knowing your HIV status, would it be negatively or positively?

**[PARTICIPANT]**: I would react positively

**[INTERVIEWER]:** Did you understand what positively means?

**[PARTICIPANT]:** Yes

**[INTERVIEWER]:** since your partner was notified up to now how has your relationship been?

**[PARTICIPANT]**: its fifty fifty

**[INTERVIEWER]:** How do you think a relationship would be if a partner has tested HIV positive

**[PARTICIPANT]**: it depends how long the relationship has been, and if they love each other

**[INTERVIEWER]:** Has PNS affected you or your relationships in anyway?

**[PARTICIPANT]**: No, it has not affected me in anyway.

**[INTERVIEWER]**: Has your relationships been the same since you notified your partners about your HIV status

**[PARTICIPANT]**: Yes, they are the same I haven't noticed any difference

**[INTERVIEWER]**: your partners were notified by peer mobilisers?

**[PARTICIPANT]**: yes, by peer mobilisers

**[INTERVIEWER]:** Are there any of your partners who haven't been notified to know their HIV status?

**[PARTICIPANT]**: no, they all have been notified.

**[INTERVIEWER]:** Have you disclosed your HIV status to anyone else apart from your partners

**[PARTICIPANT]**: Yes, my family, my mother, my siblings and health care workers.

**[INTERVIEWER]**: how much did you share with them?

**[PARTICIPANT]**: I told them when I knew I was infected and how it has changed my life

**[INTERVIEWER]**: since you disclosed has it affected you in anyway?

**[PARTICIPANT]**: No

**[INTERVIEWER]:** how has it affected the people you have disclosed to?

**[PARTICIPANT]**: no.

**[INTERVIEWER]**: we will now talk about safety of the partner notification services

**[PARTICIPANT]**: okay

**[INTERVIEWER]**: when your partners were told about your status did you experience any safety issues?

**[PARTICIPANT]:** No there have been no quarrels because I used mobilisers to tell them.

**[INTERVIEWER]**: since you disclosed your status, have you missed clients because of your status?

**[PARTICIPANT]:** No

**[INTERVIEWER]**: Are there any other issues that came out with your partners during the disclosure process?

**[PARTICIPANT]:** No, no issues came out.

**[INTERVIEWER]**: since you disclosed up to now have you had any misunderstandings with your family concerning your HIV status.

**[PARTICIPANT]:** No, I haven't

**[INTERVIEWER]**: Are there any other issues that came up when your partner was informed of your HIV status

**[PARTICIPANT]**: No there were no issues

**[INTERVIEWER]**: please let's proceed to the next topic, estimating the number of partners you have had for the past one year, how did we discuss about your sexual partners during our first interview? How many were they? you had reported they were two, are they still those two or you have remembered others which you never reported during the first visit.

**[PARTICIPANT]:** they were two.

**[INTERVIEWER]**: Between these two partners, do any of them infected you with HIV virus?

**[PARTICIPANT]:** No, they didn't

**[INTERVIEWER]**: do you think there is any importance of them knowing your HIV status?

**[PARTICIPANT]:** Yes, because sometimes we have sex without using protection

**[INTERVIEWER]**: do you think these are all the sexual partners you had for the last one year or have you forgotten some of them?

**[PARTICIPANT]:** its hard remembering all of them.

**[INTERVIEWER]**: why?

**[PARTICIPANT]:** because some of them live far away, while some we met only once

**[INTERVIEWER]**: do you have any other partners apart from the ones you told me on our initial interview

**[PARTICIPANT]:** no

**[INTERVIEWER]**: Do you think it's possible to tell me an estimate of the number of sexual partners you've had for the past one year?

**[PARTICIPANT]:** No

**[INTERVIEWER]**: Why do you think it's not possible?

**[PARTICIPANT]:** sometimes you just forget, or you fear for your life

**[INTERVIEWER]**: okay can you tell us some of the ways in which you would invite your sexual partners to come for testing?

**[PARTICIPANT]:** Yes, peer mobilisers, providing self-test kits

**[INTERVIEWER]**: okay, are you aware of counsellors calling the partners and inviting them for testing.

**[PARTICIPANT]:** yes

**[INTERVIEWER]**: what challenges do you think you would face while telling your partners about your HIV status and asking them to go for testing?

**[PARTICIPANT]:** Disclosing is the hardest part

**[INTERVIEWER]**: what is the easiest way of telling your partner that you are HIV infected

**[PARTICIPANT]:** through giving them the self-test

**[INTERVIEWER]**: is there any other way

**[PARTICIPANT]:** yes, through counsellors, peer mobilizers

**[INTERVIEWER]**: there is a difference between counsellors and peer mobilisers

**[PARTICIPANT]**: yes, through counsellors

**[INTERVIEWER]**: do you have any opinions about PNS?

**[PARTICIPANT]**: I think it's a good strategy, but confidentiality should be maintained and you should be more supportive.

**[INTERVIEWER]**: What other challenges do you foresee while people are trying to inform their partners about their HIV status.

**[PARTICIPANT]**: I think quarrels, it may lead to people ending their relationships as they may blame each other

**[INTERVIEWER]**: what do you think is the easiest way of helping someone's partner know their HIV status?

**[PARTICIPANT]**: I think it is by providing self-test kits

**[INTERVIEWER]**: do you foresee any benefits of PNS

**[PARTICIPANT]**: Yes, people who test positive are helped to start medication, support and the one who are still negative are advised on behaviour change and prep

**[INTERVIEWER]**: we will now discuss the practical part of self PNS implementation in support of HIV self pns

**[PARTICIPANT]**: okay

**[INTERVIEWER]**: there are some pictures here which I will show you, which will include the person infected, mobiliser and counsellor. The peer mobiliser will talk to the partner of the index, whom he will invite for a test at the facility

**[PARTICIPANT]: yes**

**[INTERVIEWER]**: between these methods which on do you think is the best one?

**[PARTICIPANT]**: I think using the peer mobiliser providing the self-test is the most effective

**[INTERVIEWER]**: why do you prefer this

**[PARTICIPANT]**: because there will confidentiality between the partners

**[INTERVIEWER]**: do you think there is other strategy apart from these ones that someone can use?

**[PARTICIPANT]**: No, I don't so

**[INTERVIEWER]**: How long did you take before informing your partner to come for testing?

**[PARTICIPANT]**: It took me one month.

**[INTERVIEWER]**: were you immediately told about partner notification after you tested positive?

**[PARTICIPANT]:** yes, the counsellor wanted to know if I had partners and if was ready to inform them about my status and how he would reach

**[INTERVIEWER]**: how long do you think someone who has tested positive should be given before they can provide their partners for testing?

**[PARTICIPANT]**: I think they should give some time.

**[INTERVIEWER]**: like how long?

**[PARTICIPANT]**: two weeks

**[INTERVIEWER]**: why should they be given two weeks?

**[PARTICIPANT]**: I think the counsellor should concentrate on my health because its more important.

**[INTERVIEWER]**: could you come up with other communication strategies to help the health provider reach your partners

**[PARTICIPANT]**: I think through peer educators and advertisement

**[INTERVIEWER]**: do you have any recommendations on how the counsellor can easily reach to your partners to come for HIV testing?

**[PARTICIPANT]**: I would recommend that people should know their status and their partners because it's not good if you know that you are infected and then you won't inform your partner to go for testing.

**[INTERVIEWER]**: is there anything else you would like us to discuss about PNS...

**[PARTICIPANT]**: I think both peer mobilisers and counsellors should maintain high level of confidentiality because you know what kind of a reaction you would get from someone's partner who test HIV positive.

**[INTERVIEWER]**: Do you have anything to add about this study ...?

**[PARTICIPANT]**: This study should continue, you really help people's healthy and economically so that they can be self-reliable.

**[INTERVIEWER]**: do you think this strategy can help in reducing HIV transmissions

**[PARTICIPANT]**: Yes, it does as it helps partners and especially families live together well after they have become aware of their HIV status instead of waiting until it's too late.

**[INTERVIEWER]**: since you knew your status up to now do you have any advice for regarding PNS

**[PARTICIPANT]**: the best thing to do is accepting your status and following the doctor's instructions and know the best ways that someone can live with HIV and you can live well.

**[INTERVIEWER]**: since you were diagnosed up to now, do you see any changes?

**[PARTICIPANT]**: yes, before I was very sick, I had lost a lot of weight, right now I feel good, my appetite is back, I sleep well, and my life is back to normal.

**[INTERVIEWER]**: what was the main reason that made you start medication?

**[PARTICIPANT]**: I was becoming sick frequently, loss of appetite and general fatigue

**[INTERVIEWER]**: have you seen any other health changes in your life since you started taking medications?

**[PARTICIPANT]:** yes, I feel way better, am more energetic I can work and provide for myself, I feel fine

**[INTERVIEWER]:** thank you very much for giving us your time, today we were talking about PNS, it is a study, we are trying to see if someone especially MSM, if you test HIV positive can you bring your partners for testing and if these partners can decide to start medication if positive or prep if they are HIV negative .do you have any question?

**[PARTICIPANT]**: No.

**[INTERVIEWER]:** thank you very much for your time, if you don't have any other question, thank you for coming today

**[PARTICIPANT]:** thankyou

**[INTERVIEWER]:** your welcome.
